# uORF-mediated translational control of CAK and Cdk activation in response to low nutrients in yeast

**DOI:** 10.1038/s44319-026-00916-z

**Published:** 2026-09-05

**Authors:** Heidi M Blank, Eun-Gyu No, Ainsley E Nelson, Abigail Payne, Sofia Lykidis, Michael Polymenis

**Affiliations:** Department of Biochemistry and Biophysics, Texas A&M University, College Station, TX USA

**Keywords:** Cell Cycle, Translation & Protein Quality

## Abstract

Cyclin-dependent kinases (Cdks) require activating T-loop phosphorylation, a modification considered constitutive. Here, we examine the regulation of the Cdk-activating kinase, Cak1, in budding yeast. We measure Cak1 levels and the activating T169 phosphorylation of Cdc28 (the budding yeast Cdk) in different nutrients. The abundance of Cak1 and T169 phosphorylation is reduced in cells that proliferate very slowly or enter quiescence. A small upstream open reading frame (uORF) in CAK1 represses Cak1 synthesis, especially in poor growth conditions. Eliminating the uORF increases Cak1 levels but does not alter proliferation kinetics under most laboratory contexts. Instead, it reduces the viability of quiescent cells. In cells lacking several type 2 C protein phosphatases, which remove the T169 phosphorylation, initiation of cell division is accelerated in the absence of the uORF in CAK1. Our results suggest an unexpected layer of control, impinging on the activating phosphorylation of the Cdk. The uORF-mediated repression of Cak1 synthesis directly couples protein synthesis to the activity of the core cell cycle machinery.

## Synopsis

Levels of the Cdk-activating kinase Cak1 and the active Cdk Cdc28 are reduced in yeast in poor growth conditions, because Cak1 translation is repressed by an uORF, coupling the cell’s protein-synthesis capacity to the activation of master regulators of division.

- An uORF within the CAK1 transcript represses Cak1 synthesis in poor growth conditions. Eliminating this uORF increases Cak1 abundance and decreases the viability and fitness of cells entering or exiting quiescence.
- Removing the CAK1 uORF in cells lacking type 2 C protein phosphatases results in elevated T169 phosphorylation and accelerated initiation of cell division.

## Abstract (web summary)

Levels of the Cdk-activating kinase Cak1 and the active Cdk Cdc28 are reduced in yeast in poor growth conditions, because Cak1 translation is repressed by an uORF, coupling the cell’s protein-synthesis capacity to the activation of master regulators of division.

## Graphical abstract

**Figure:**
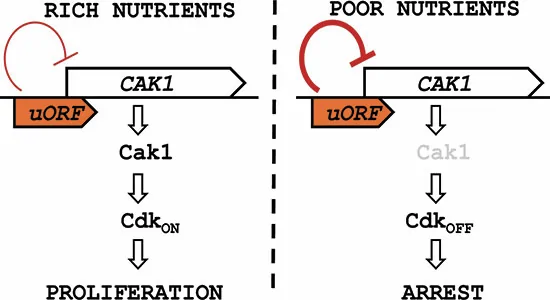

## Introduction

From late in the G1 phase of the cell cycle until anaphase, a rising Cdk activity drives cell cycle progression (Polymenis, 2022; Morgan, 2007). Active Cdks are serine/threonine protein kinases. The Cdks are inactive as monomers and require binding to a regulatory cyclin subunit to form an active Cdk-cyclin heterodimer. When a cyclin binds to the Cdk, the conformation of the Cdk’s T-loop domain and a small nearby helix change, allowing protein substrates and ATP to access the active site of the kinase (Pavletich, 1999). In addition to cyclin binding, a conserved threonine residue in the T-loop must also be phosphorylated for Cdk activation. (Russo et al, 1996). T-loop phosphorylation is essential for Cdk function. Surprisingly, however, a mutation mimicking constant T-loop phosphorylation at the same site is also lethal unless the Cdk has more mutations elsewhere in the protein (Cross and Levine, 1998). Cells regulate the activity of Cdk complexes in many ways, as one would expect for the “master” regulator of cell division. For example, interactions with other proteins activate or inhibit the kinase, while periodic phosphorylations at its N-terminus inhibit it (Polymenis, 2022; Morgan, 2007). Yet, cells do not periodically phosphorylate and dephosphorylate the T-loop to adjust their Cdk activity in the cell cycle (Kaldis, 1999; Cross and Levine, 1998). The seemingly constitutive nature of the activating T-loop phosphorylation has raised long-standing questions about its physiological role in cell proliferation (Blank et al, 2025).

Cdk-activating kinases (CAKs) are the enzymes that phosphorylate Cdks at the T-loop (Kaldis, 1999; Blank et al, 2025). CAKs themselves are part of the large Cdk family. In metazoans, Cdks that do not have direct cell cycle roles but instead transcriptional ones serve as CAKs (Fisher and Morgan, 1994; Fisher, 2005; Fisher et al, 1995; Shiekhattar et al, 1995). In budding yeast, Cak1 is the only CAK (Kaldis et al, 1996; Thuret et al, 1996; Espinoza et al, 1996). Although Cak1 targets other proteins, for example, in meiosis (Whinston et al, 2013; Wagner et al, 1997), its single essential function is to phosphorylate the T169 residue of Cdc28, the main Cdk required for mitotic cell divisions in this organism. There is no evidence that the abundance, activity, or localization of Cak1 is regulated in proliferating cells, nor do post-translational modifications alter it (Kaldis et al, 1998a). Likewise, the levels and activity of metazoan CAKs do not change during somatic (Tassan et al, 1994; Bartkova et al, 1996) or early embryonic cell divisions (Poon et al, 1994). The above observations add to the puzzle of why an essential modification and the ‘writer’ enzymes of that modification on the Cdk appear unregulated.

Contrary to expectations of constitutive control of Cak1 and T-169 phosphorylation, here we show that Cak1 abundance and the activating T-loop phosphorylation of Cdc28 are regulated as cells transition from nutrient-rich to nutrient-poor environments or enter quiescence. A small upstream open reading frame (uORF) represses *CAK1* mRNA translation. Cells lacking the uORF lose viability when starved and enter quiescence and are less fit in slowly proliferating steady-state chemostat cultures. Furthermore, the effects of the uORF-mediated control of Cak1 synthesis become very prominent even in cycling cells when the counteracting phosphatases that remove the Cdk T169 phosphorylation are absent. Our results suggest a previously unappreciated layer of Cdk regulation in coupling cell growth with cell division in poor conditions. Lowering T-loop phosphorylation may safeguard against mitotic cycling in unfavorable contexts.

## Results

### Cak1 levels and the activating T169-Cdc28 phosphorylation vary in different conditions

**Figure 1:**
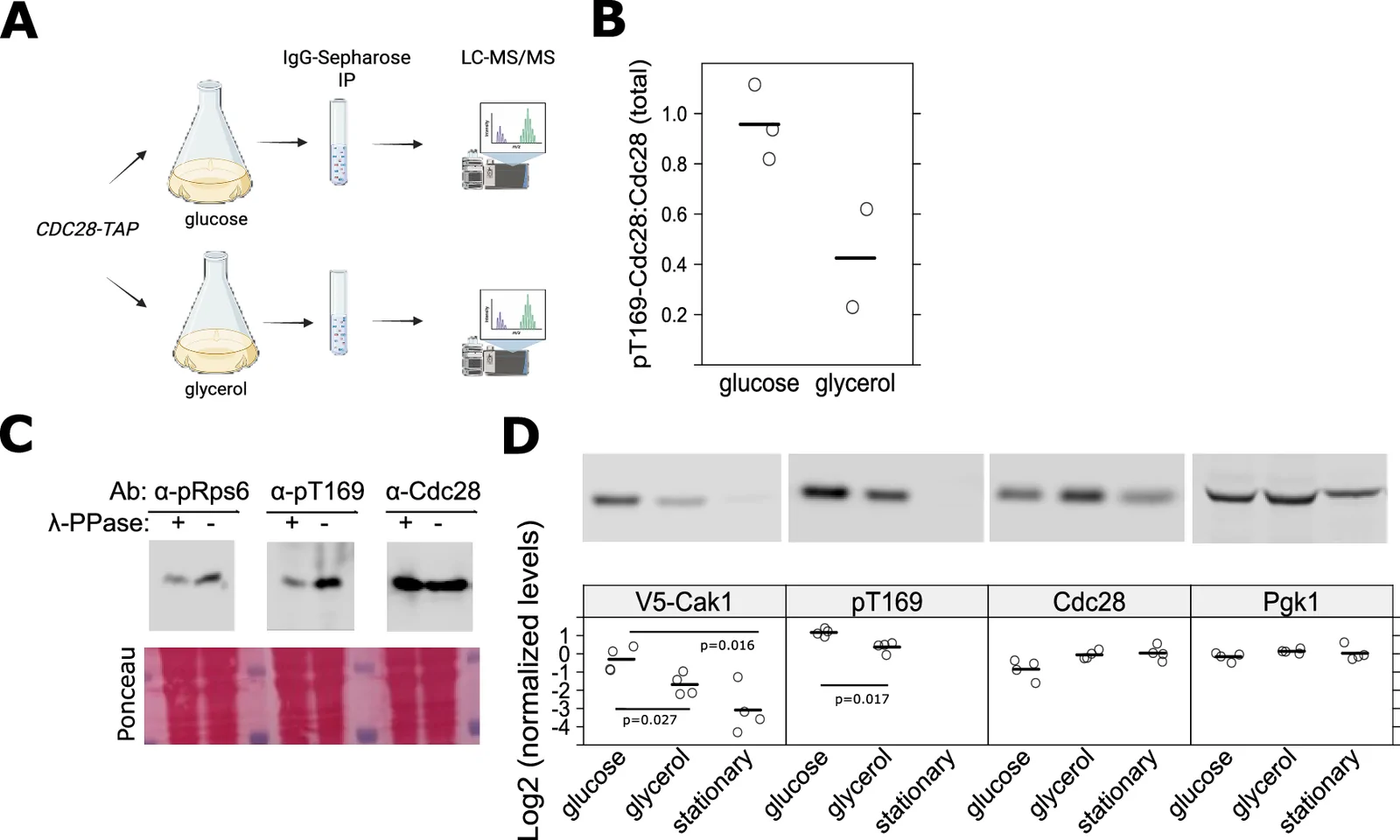
Cdc28 T169 phosphorylation and Cak1 protein levels vary with different growth conditions in batch cultures. (**A**) Schematic of the isolation of Cdc28-TAP by immunoprecipitation and processing for mass spectrometry to compare the extent of T169 phosphorylation in cells growing in media with glucose or glycerol as the carbon source. (**B**) Individual data points represent the relative abundance of recovered Cdc28 peptides with the T169 phosphorylation normalized to the total abundance of all Cdc28 peptides (*y* axis). Values for cells growing in glucose were normalized to one, with glycerol ratios shown relative to this baseline (*x* axis). Data are presented as a strip plot, where each open circle represents an independent biological replicate. The horizontal black line within each group indicates the mean value. Raw mass spectrometry data are provided in Dataset EV1. (**C**) An antibody against T169-Cdc28 phosphorylation. Protein extracts from exponentially proliferating cells (X2180-5A background) in rich undefined medium (YPD) were split in half and either treated (+) or not (−) with lambda phosphatase. The samples were then run on an SDS–PAGE gel and transferred to a nitrocellulose blot. The blot was stained with Ponceau to indicate loading (bottom) and then processed with the antibodies indicated on top. The α-pT169 antibody was raised in rabbits, using the peptide RAFGVPLRAY(pT)HEIVT as an antigen, corresponding to Cdc28 (see “Methods”). (**D**) Representative immunoblots of V5-Cak1, pT169-Cdc28, Cdc28, and Pgk1 are shown at the top. The strip plot graphs below were drawn as in (**B**) and display the quantification of each target from four independent experiments. Band intensities were quantified using ImageJ software. For each experiment, the signal for each quantified target was divided by the average intensity for that target in the series across all conditions for that specific strain on the same gel, and these ratios were then Log2 transformed. These Log2-transformed values are plotted on the *y* axis; the *x* axis indicates the respective growth conditions. The extracts were prepared from cells growing exponentially in batch cultures with glucose or glycerol as the carbon source or until they reached the stationary phase after being cultured for at least 3 days in media with glucose as the carbon source. In all cases, the cells expressed *CAK1* from its endogenous chromosomal location but from an N-terminal V5 epitope-tagged *CAK1* allele. The indicated *P* values were determined using robust bootstrap ANOVA (t1waybt function) followed by post hoc tests (mcppb20 function) from the WRS2 R package. Source data are available online for this figure.

We decided to test if Cak1 abundance and the activating phosphorylation of Cdc28 at T169 vary in different media because past studies overwhelmingly focused on cells continuously dividing in standard rich laboratory media. In addition, the only instance in the literature for any control over Cak1 levels in *S. cerevisiae* vegetative cells was when they were in the stationary phase, at which point the protein’s abundance dropped significantly (Kaldis et al, 1998a). We isolated Cdc28-TAP by immunoprecipitation from cells cultured in media with glucose, a preferred carbon source, or glycerol (Fig. 1A). The isolated protein was then examined by mass spectrometry to see if there were any changes in the fraction of the Cdc28 protein phosphorylated at T169. Indeed, in the glycerol-grown cells, the fraction of T169-phosphorylated Cdc28 was reduced by about half (Fig. 1B). To detect T169 phosphorylation in follow-up experiments, we used a custom anti-pT169 antibody (see Methods) to provide independent validation of the mass spectrometry data and because it allows for faster, simpler, cheaper, and more sensitive detection. The custom antibody detected a protein of the expected size for Cdc28 (Fig. 1C, compare the middle and right blots). The signal was also sensitive to phosphatase treatment (Fig. 1C, middle blot, compare the left and right lanes) to the same extent as the signal from a previously validated phospho-specific antibody against another protein, Rps6 (Fig. 1C, left blot, compare the left and right lanes). While incomplete dephosphorylation is the most likely explanation for the residual signal, it is also possible that some residual affinity of these antibodies for their non-phosphorylated target may remain.

To monitor Cak1 levels, we used strains carrying an allele encoding Cak1 with a V5 epitope tag at its N-terminus (see “Methods”). We could then detect from the same samples the abundance of Cak1 (with an anti-V5 antibody), Cdc28, and Pgk1 (with commercial antibodies), and phospho-T169-Cdc28 (with our custom antibody). For each experiment, the signal for each quantified target was internally normalized by dividing its intensity by the average intensity for that target across all conditions on the *same* gel, and then Log2 transformed (Fig. 1D legend). This internal mean normalization, the “expressed ratio” method (Spellman et al, 1998), displays protein abundances in the different conditions we tested. Unlike other normalizations (e.g., to a loading control), this approach calibrates each protein’s signal relative to its average across the experimental series on a single gel. It accounts for gel-to-gel and exposure differences among independent replicates. The Log2 transformation shows protein levels with equal weight around a central baseline (zero), yielding a symmetrical, biologically intuitive view of protein level fluctuations across conditions.

Our results suggest that the levels of Cak1 were the highest in exponentially proliferating cells in glucose-containing media, lower in glycerol-containing ones, and, as expected based on (Kaldis et al, 1998a), even lower in stationary phase cells (Fig. 1D, left panel). In agreement with our mass spectrometry data (Fig. 1B), T169 phosphorylation was lower in cells proliferating with glycerol as the carbon source, compared to cells proliferating with glucose, but undetectable in stationary phase cells (Fig. 1D, second panel from the left). In contrast, total Cdc28 (and Pgk1; a glycolytic enzyme used as a reference) levels did not change in all these conditions. We conclude that the levels of the activating Cdk phosphorylation (but not the levels of the Cdk itself) and the abundance of Cak1, the writer activating kinase, respond to growth conditions.

### *CAK1* expression is regulated translationally, in part through a uORF

**Figure EV1:**
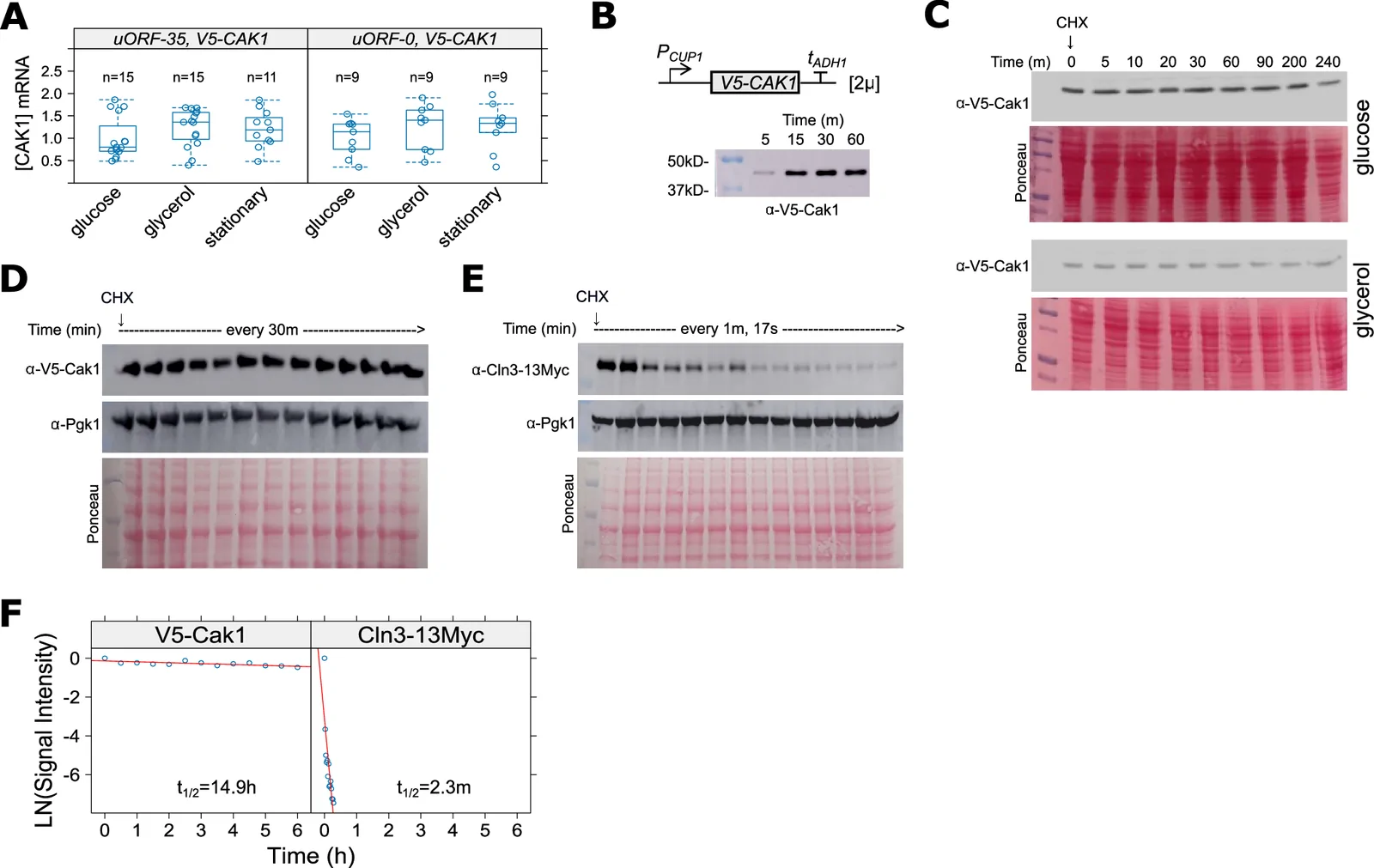
*CAK1* expression is not regulated by transcription or proteolysis as a function of growth. (**A**) Relative steady-state levels of *CAK1* mRNA from multiple independent experiments (*y* axis) across the indicated growth conditions (*x* axis). Strains and growth conditions are identical to those in Fig. 1C. Boxplots illustrate the distribution of normalized levels across biological replicates; the horizontal line represents the median, box boundaries indicate the interquartile range (IQR), and whiskers extend to the minimum/maximum values within 1.5 times the IQR. Individual open circles represent independent experimental data points. mRNA levels were measured by ddPCR and normalized to the mean of exponentially growing *uORF-35 V5-CAK1* cells (first box). No significant differences were found between strains or conditions (*P* > 0.05, Kruskal–Wallis test). Every datapoint is from a separate experiment. (**B**) (Top) Schematic of the plasmid used for copper-inducible synthesis of V5-Cak1. (Bottom) Immunoblot of cells (BY4742 background) carrying the plasmid, showing V5-Cak1 protein levels (detected via an anti-V5 antibody) at the indicated times after induction with CuSO_4_. (**C**) V5-Cak1 protein stability in glucose and glycerol. Exponentially proliferating cells (BY4742 background) carrying the pCUP1-V5-CAK1 plasmid (described in **B**) were induced for 20 m with 0.5 mM CuSO_4_ and then treated with cycloheximide (CHX; 100 μg/mL) to block translation. V5-Cak1 levels were monitored by immunoblotting (top panels) in glucose (upper set) and glycerol (lower set) media. Total protein loading is shown via Ponceau S staining (bottom panels). (**D**) A similar cycloheximide-chase experiment as in (**C**) was performed in YPD using strain MSP258, expressing V5-Cak1 from its endogenous locus. Immunoblots show levels of V5-Cak1 (top) and Pgk1 (middle) as a loading control. Total protein is shown by Ponceau S staining (bottom). (**E**) A similar experiment to (**D**) was performed using strain HB490, which expresses Cln3-13Myc from its endogenous locus. (**F**) The natural logarithm (LN) of signal intensities (*y* axis), normalized to Pgk1, is plotted against time (*x* axis). Red lines represent linear regression fits. V5-Cak1 (left) remains stable over the 6-h course, whereas Cln3-13Myc (right) is rapidly degraded. Estimated half-lives, t_1/2_, were derived from the slopes of the regression fits. Source data are available online for this figure.

To understand the regulation of Cak1 levels, we first quantified *CAK1* mRNA abundance in the same growth conditions described in Fig. 1D. We used digital droplet PCR (ddPCR) because it is a quantitative, highly sensitive method well-suited for detecting even small differences in mRNA abundance (Quan et al, 2018). We found that steady-state levels of *CAK1* mRNA were not significantly different among exponentially proliferating cells in glucose or glycerol-containing media, or in stationary phase cells (Fig. EV1A, left). Hence, transcriptional control mechanisms are unlikely to explain the observed differences in Cak1 protein abundance.

Since many cell cycle proteins are subject to regulated degradation, we next measured the apparent half-life of Cak1 in glucose- and glycerol-containing media. To study Cak1 protein stability, we first constructed a high-copy (2 μ) plasmid enabling copper-inducible (*CUP1* promoter) expression of *V5-CAK1* (Fig. EV1B, top). As expected, V5-Cak1 synthesis was rapidly induced upon the addition of CuSO_4_ (0.5 mM) to cells harboring this plasmid (Fig. EV1B, bottom). In separate experiments, we examined otherwise wild-type cells (strain BY4742) carrying this plasmid, growing with either glucose or glycerol as the carbon source. After a 20 m induction to drive V5-Cak1 synthesis, we added cycloheximide (CHX) to block new protein synthesis. After normalizing the Cak1 signal (Fig. EV1C, top) against the total protein signal at each time point (from the Ponceau-stained blots; Fig. EV1C, bottom), there was little degradation of Cak1 in either the glucose- or glycerol-containing media. We extended these observations using strains expressing at endogenous levels V5-Cak1 (Fig. EV1D) or Cln3-13Myc (Fig. EV1E), to query the stability of these proteins after addition of CHX (100 μg/mL). Cln3 is a G1 cyclin known to be extremely unstable, with a half-life of a few minutes (Yaglom et al, 1995), and served as the control. We obtained a 2.3 m estimated half-life for Cln3-13Myc, but little degradation for V5-Cak1, with an estimated half-life of 14.9 h (Fig. EV1F). These results argue against Cak1 destabilization as the primary mechanism explaining the observed differences in Cak1 protein abundance in glucose versus the poorer glycerol media. We note that the general notion that Cak1 is a very stable protein agrees with prior studies (Christiano et al, 2014; Kaldis et al, 1998a), and with Cak1’s listed half-life (16.9 h) on the Saccharomyces Genome Database (SGD).

**Figure 2:**
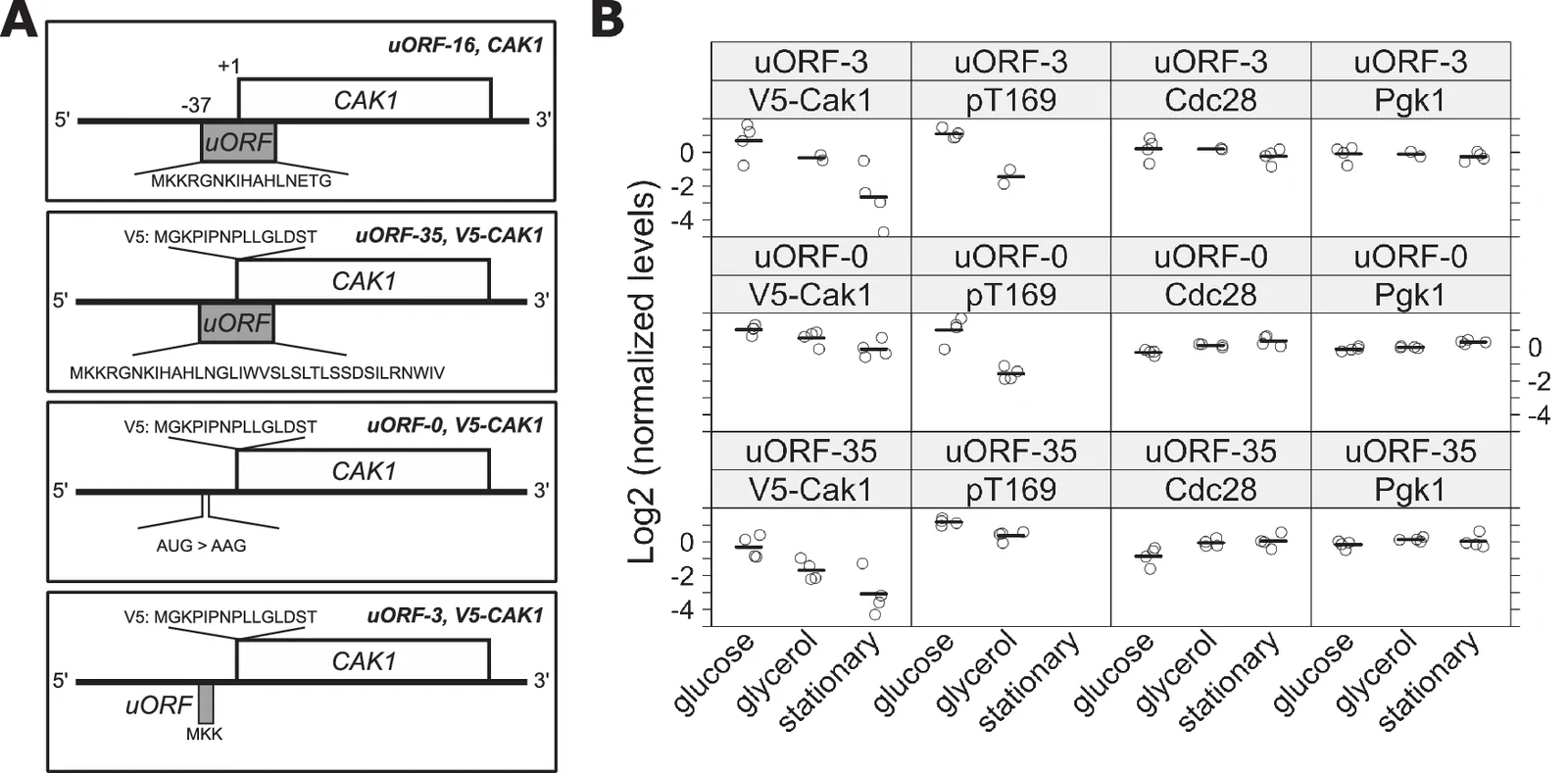
A uORF represses Cak1 synthesis, yet T169 phosphorylation is robust to changes in Cak1 levels. (**A**) A schematic of the *CAK1* upstream genomic region. The wild-type uORF at position -37 encodes a 16-amino acid peptide. The engineered uORF changes are shown below, each with an N-terminal V5 epitope tag fused in-frame with the *CAK1* ORF. (**B**) Strip plots showing the quantification of V5-Cak1, pT169-Cdc28, Cdc28, and Pgk1 in the indicated strains, drawn as in Fig. 1. Data for the uORF-35 background are reproduced from Fig. 1D for comparison. All experiments and analyses were performed as described in Fig. 1D. All the strains were in the prototrophic X2180-5A background. There were four biological replicates in all experiments except in glycerol-grown uORF-3 cells, which were measured in two separate experiments. Source data are available online for this figure.

We then focused on potential mechanisms of translational control to explain the differing abundance of Cak1 under various growth conditions. We noticed that *CAK1* has a uORF, encoding a 16-residue peptide, which initiates 37 nucleotides upstream of the *CAK1* start codon and terminates downstream (marked as uORF-16 in Fig. 2A, top). The uORF could explain the differences in the levels of Cak1 we described (Fig. 1D). Ribosomes initiating at the uORF would bypass the start codon of *CAK1*, repressing Cak1 protein synthesis. The uORF-mediated repression of Cak1 synthesis would be disproportionately greater in poor growth conditions, when the cell’s ribosome content is lower, according to the classic kinetic model of translation (Lodish, 1974). To test the uORF’s role, we generated strains carrying a mutation at its AUG start codon to AAG (marked as uORF-0 in Fig. 2A, second from bottom). Introducing the V5 epitope to monitor Cak1 abundance also extended the peptide produced by the uORF to 35 residues (marked as uORF-35 in Fig. 2A, second from top). Lastly, to test whether it is the presence of a uORF in general, or specifically the uORF-encoded peptide that impacts Cak1 synthesis, we introduced a stop codon that terminates the uORF synthesis after only three residues (marked as uORF-3 in Fig. 2A, bottom).

In the uORF mutants, we monitored the Cak1, T169-phosphorylation, Cdc28, and Pgk1 levels by immunoblotting (Fig. 2B). When *uORF-0* cells proliferated with glycerol as the carbon source or were in the stationary phase, the levels of Cak1 were derepressed, albeit not completely, compared to *uORF-35* cells (compare the middle left panel to the bottom left panel). In contrast, in *uORF-3* cells, Cak1 levels remain repressed as in *uORF-35* cells (compare the top left panel to the bottom left panel). We conclude that the presence of the uORF represses Cak1 synthesis in a growth-dependent manner. The uORF-encoded peptide is unlikely to be responsible for this regulation. Instead, the repression likely arises from ribosomal re-initiation failure, whereby ribosomes that successfully translate the uORF-3 are less likely to re-initiate translation at the downstream *CAK1* start codon. We note that the uORF-mediated repression was not the result of changes in the steady-state levels of the *CAK1* transcript (Fig. EV1A, right). Surprisingly, although Cak1 levels were derepressed in stationary phase uORF-0 cells, the levels of T169 phosphorylation were not, and remained undetectable (Fig. 2B, second from the left column of panels—compare the middle to the bottom panel). The levels of Cdc28 and Pgk1 remained largely unaffected in all conditions and mutants (Fig. 2B, 3rd and 4th columns of panels from the left). These results suggest that the uORF regulates Cak1 levels in different growth conditions, but the levels of the activating phosphorylation on the Cdk are robust to those changes.

**Figure 3:**
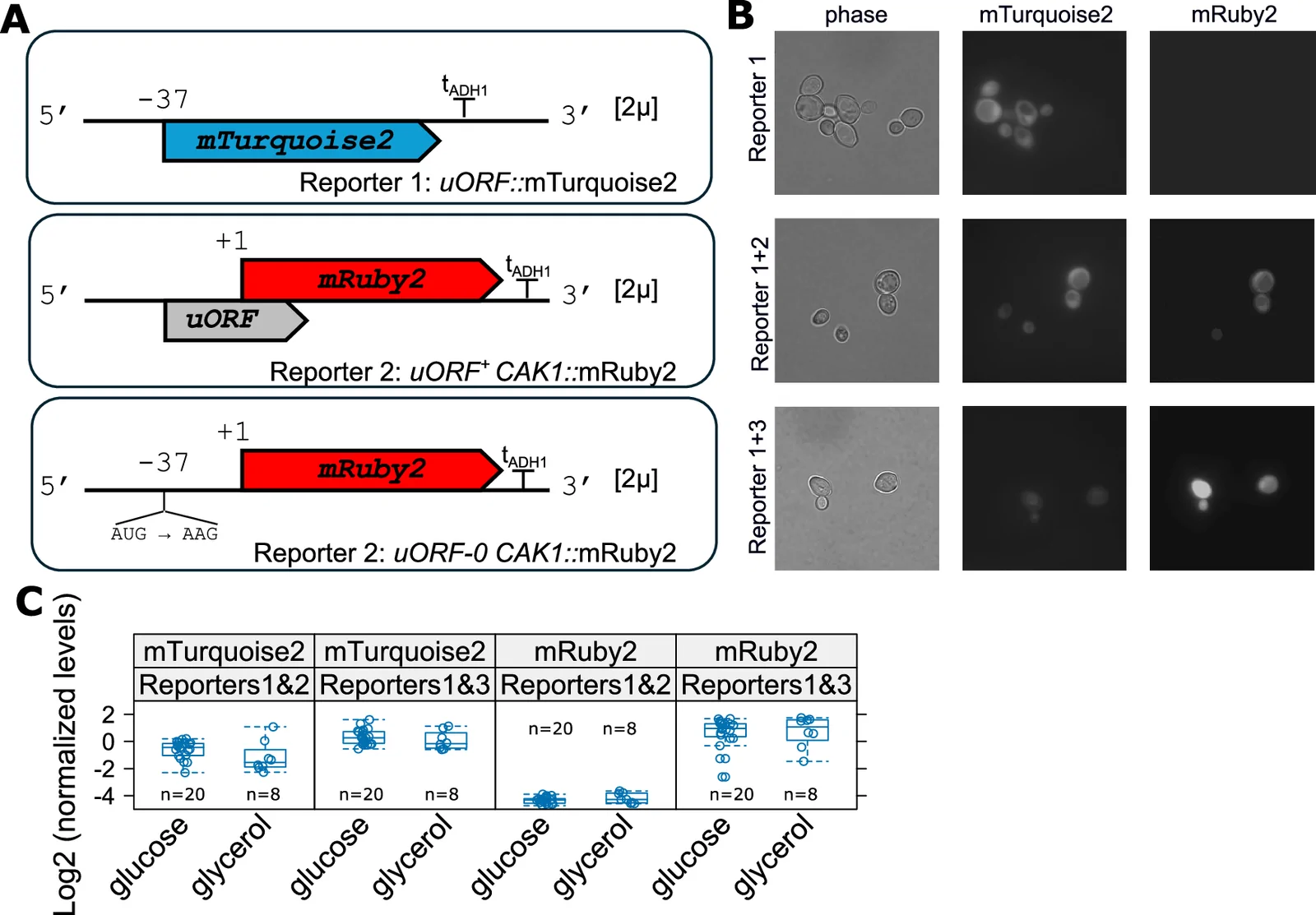
The *CAK1* uORF is expressed and represses downstream translation. (**A**) Schematic of high-copy reporter plasmids. Reporter 1 (top) replaces the uORF coding sequence with mTurquoise2 (uORF expression). Reporter 2 (middle) replaces the *CAK1* open reading frame with mRuby2 and contains the uORF. Reporter 3 (bottom) is the same as Reporter 2 but with a mutated uORF start codon (corresponding to the uORF-0 allele in Fig. 2A). (**B**) Representative images of cells carrying the indicated reporters. Top row: Cells with Reporter 1 only (uORF-mTurquoise2, blue) show blue fluorescence, but not red. Middle/Bottom rows: Cells co-expressing Reporter 1 with either Reporter 2 (containing the uORF) or Reporter 3 (uORF-0). Note the marked increase in mRuby2 (red, *CAK1* expression) in the Reporter 3 (uORF-0) co-transformants compared to Reporter 2, demonstrating uORF-mediated repression. The cells were imaged live. The exposure was 500 ms for the blue channel and 200 ms for the red channel. (**C**) Boxplots of single-cell mTurquoise2 (uORF) and mRuby2 (*CAK1*) fluorescence intensity from strain BY4743 co-transformed with Reporter 1 and either Reporter 2 (with uORF) or Reporter 3 (uORF mutated). Cultures were grown in glucose or glycerol, as indicated. Fluorescence per cell was quantified with flow cytometry. Boxplots illustrate the distribution of normalized levels across biological replicates. The horizontal line within each box represents the median, and the box boundaries indicate the interquartile range (IQR) (25th to 75th percentiles). Whiskers (dashed lines) extend to the minimum and maximum values within 1.5xIQR of the box hinges. Individual open circles represent the underlying data points, jittered horizontally to reduce overlap and visualize the sample density. Every datapoint is from a separate experiment, scoring thousands of cells in each case. Source data are available online for this figure.

To provide independent evidence of uORF’s expression and its impact on Cak1 levels, we generated plasmids encoding fluorescent proteins in their place (Fig. 3A). In cells carrying these plasmids, mTurquoise2-associated fluorescence reported on uORF expression, while mRuby2-associated fluorescence reported on Cak1 (Fig. 3B). These plasmids were maintained at a high copy number in cells, but the sequences driving expression of the fluorescent reporters were from the *CAK1* locus (see Methods). Our results show that mTurquoise2-associated fluorescence was readily detectable in all conditions we tested (Fig. 3B,C), arguing that the start codon of the uORF is recognized by scanning ribosomes. Furthermore, the significant increase (~tenfold) in mRuby2-associated fluorescence when the uORF start codon was mutated (Fig. 3C, compare the last two panels to the right) strongly supports the notion that the uORF represses Cak1 synthesis. We note that the magnitude of the repression appears greater with these reporters compared to our results from immunoblotting (Fig. 2B, twofold to threefold), and the regulatory difference between glucose and glycerol was blunted. This is likely an artifact of using high-copy plasmids in this specific assay.

**Figure 4:**
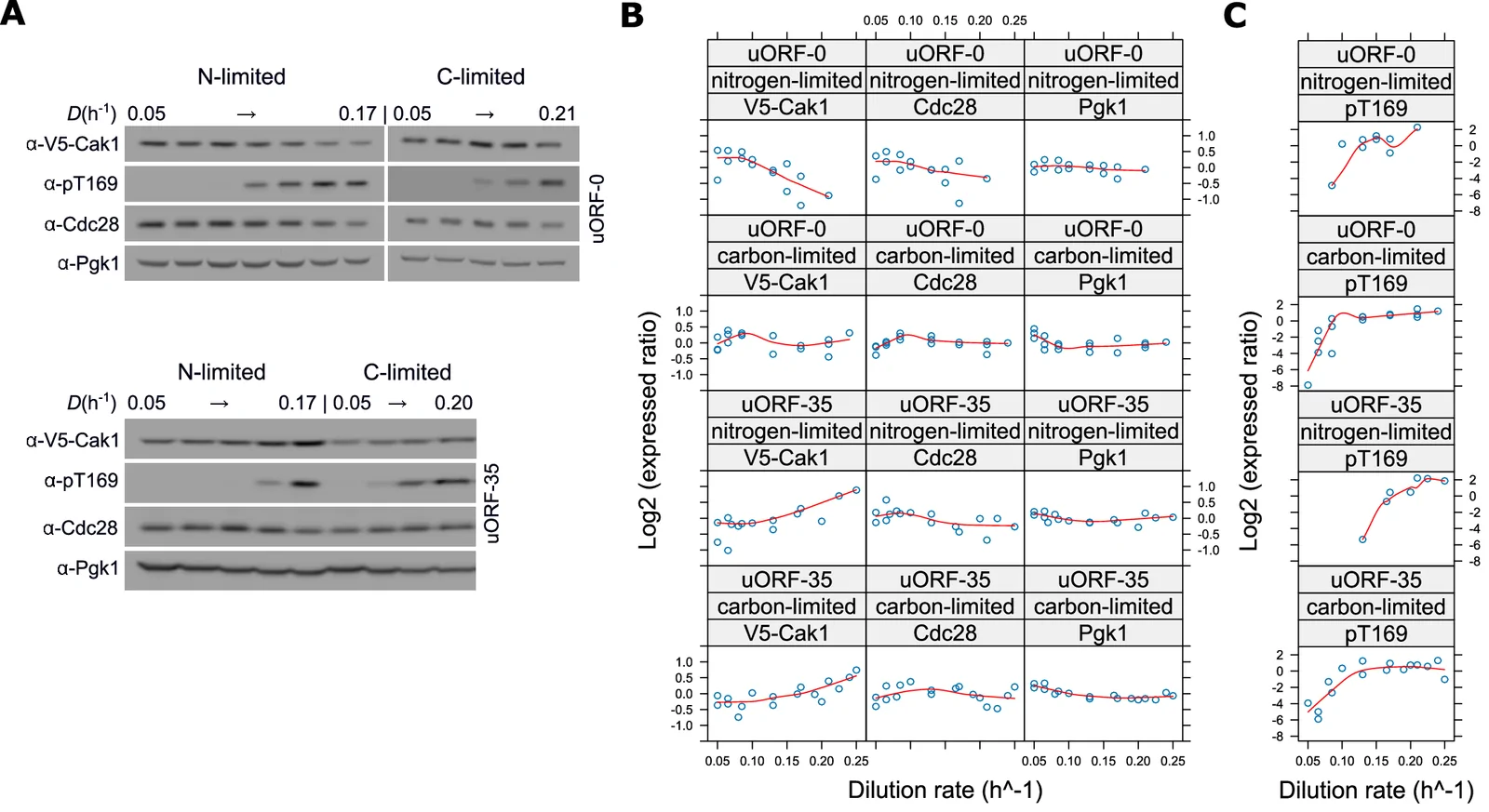
Cak1 levels and T169 phosphorylation respond to growth rate changes with a different dynamic range. (**A**) Representative immunoblots of V5-Cak1, pT169-Cdc28, Cdc28, and Pgk1, with antibodies indicated, from chemostat cultures limited for nitrogen (N; ammonium sulfate) or carbon (C; glucose), for the strains shown. Samples were collected at the indicated dilution (*D*) rates (in h^−1^). (**B**, **C**) The graphs display the quantification of each indicated target from independent chemostat experiments under the limitations shown for each strain. Band intensities were quantified, and their Log2-transformed expressed ratios were calculated as in Fig. 1, for each series across the different dilution rates, and plotted on the *y* axis; the *x* axis indicates the respective dilution rates. Loess curves are shown in red. All the strains were in the prototrophic X2180-5A background. Source data are available online for this figure.

The experiments we described thus far were in cells proliferating with glucose or glycerol as the carbon source in batch cultures, where the doubling time varies approximately twofold (90 m vs. 160 m), and one cannot distinguish nutrient-specific effects from growth rate-dependent ones. In contrast, in chemostats, the growth rate is externally set by the rate of medium addition (*D*, dilution rate). It can be adjusted over a much greater range, independently of the limiting nutrient in the medium. For these reasons, we examined prototrophic *uORF-35* and *uORF-0* cells in chemostats under carbon or nitrogen limitation (Fig. 4). Over a broad range of dilution rates, corresponding to a doubling time of 3.5 h to 13.9 h, Cak1 levels decreased linearly as the growth rate was lowered in *uORF-35* cells, in both carbon- and nitrogen-limited cultures (Fig. 4A, bottom and  4B, bottom two panels in the left column). Hence, Cak1 levels respond to changes in growth rate, but not in a nutrient-specific manner. This regulation was not evident in *uORF-0* cells, where Cak1 levels were not elevated in higher dilution rates (Fig. 4A, top and  4B, top two panels in the left column). If anything, in *uORF-0* cells under nitrogen limitation, Cak1 levels might be reduced at higher dilution rates. There were also no growth rate or nutrient-specific changes in the abundance of Cdc28 and Pgk1 in these strains and conditions (Fig. 4B, second and third columns of panels). These results argue that the repressive function of the uORF in the *CAK1* transcript mediates the growth-dependent changes in Cak1 abundance.

In contrast to the steady decline of Cak1 levels as the dilution rate was lowered, the levels of T169 phosphorylation displayed a different pattern: relatively constant until the cultures had a doubling time of 6-7 h (*D* = 0.10 h^−1^), but dropping precipitously in cells proliferating at slower rates (Fig. 4C). The growth-dependent reduction in T169 phosphorylation was slightly more acute in nitrogen-limited cells, occurring at doubling times of >5 h (e.g., compare the two bottom panels in Fig. 4C). In *uORF-0* cells, the drop in T169 phosphorylation was still evident in low dilution rates, albeit it appears to have been pushed to even lower ones (e.g., in Fig. 4C, compare the second from the top to the bottom panels).

### Phenotypes of cells lacking the *CAK1* uORF

**Figure EV2:**
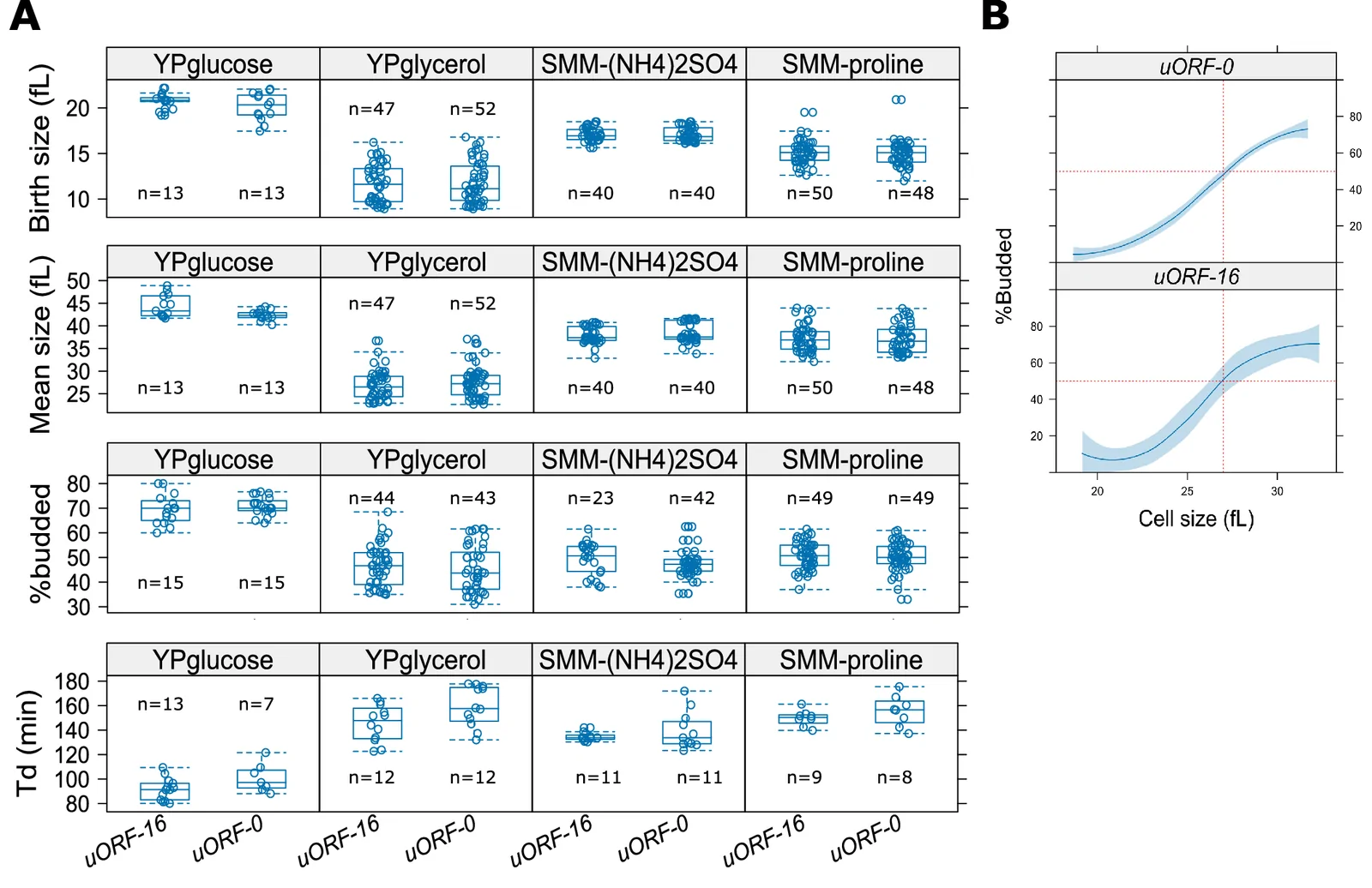
Eliminating the uORF of *CAK1* does not affect cell cycle kinetics in batch cultures. (**A**) Cell cycle parameters for the *uORF-16 CAK1* (MSP256) and *uORF-0 CAK1* (MSP257) strains are shown on the *x* axis, grown in the indicated media. The boxplots, drawn as described in Fig. EV1, show (from top to bottom): birth size, mean cell size, %budding, and doubling time (Td), on the *y* axis. Values were obtained from independent experiments. No significant differences were found between the strains (*P* > 0.05, Wilcoxon rank-sum test). Every datapoint is from a separate experiment. (**B**) Cell cycle progression of elutriated synchronous early G1 cells in YP-glycerol. The percentage of budded cells is plotted against cell size (fL) for the same strains as in (**A**). The plots show Loess curves with 95% confidence intervals. Red lines indicate the critical size for budding (the size at which 50% of cells have budded).Source data are available online for this figure.

**Figure EV3:**
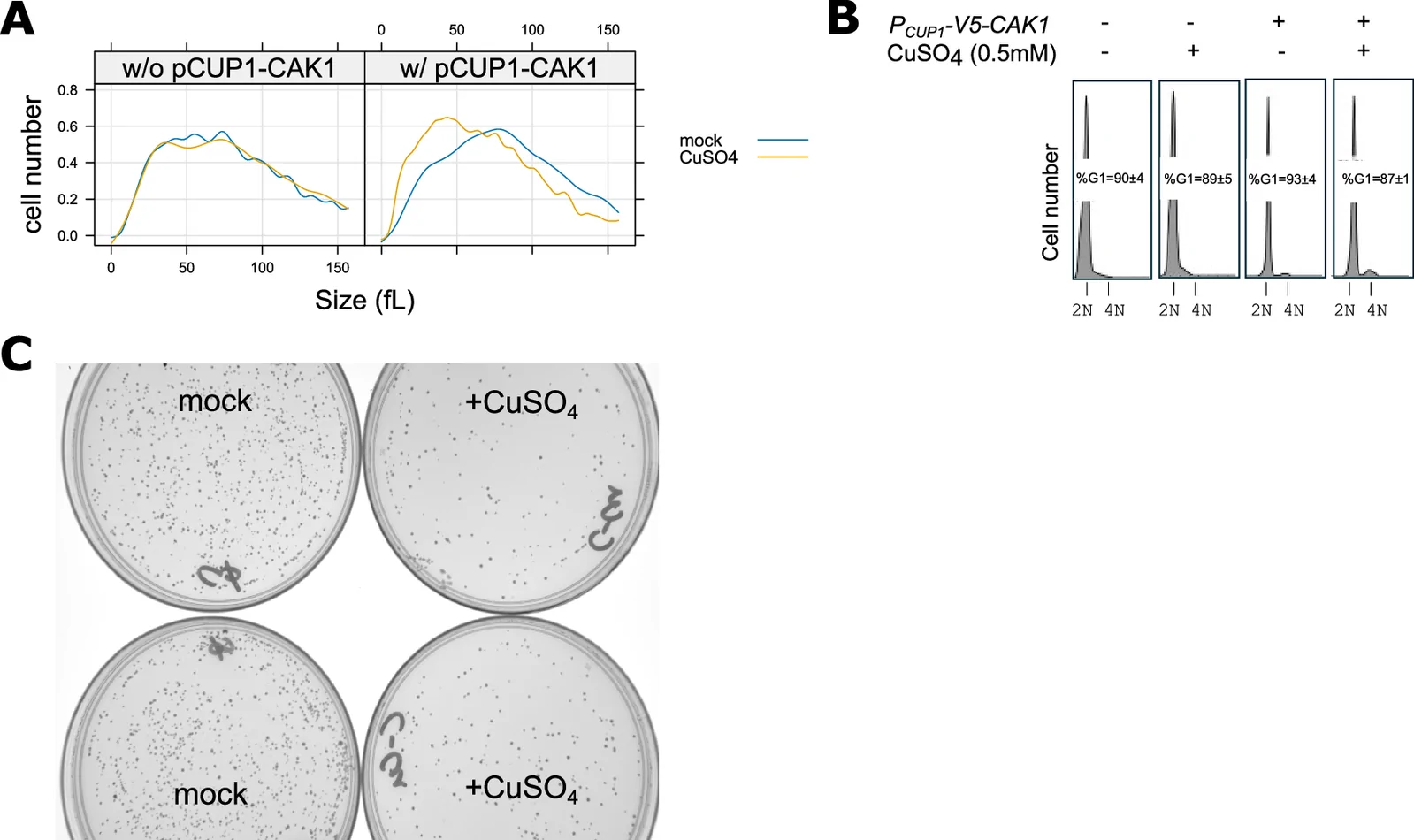
Overexpression of *CAK1* in the stationary phase reduces cell size and viability. (**A**) Cell size distribution of stationary phase cells (BY4743) with (right panel) or without (left panel) the pCUP1-V5-CAK1 plasmid. Histograms show the fraction of cell counts (*y* axis) by size (*x* axis) from 6 d cultures. Density lines represent cultures induced with 0.5 mM CuSO_4_ to express V5-Cak1 (yellow) versus mock-treated controls (blue). (**B**) DNA content analysis of the same samples shown in (**A**), measured by flow cytometry. Histograms show cell counts (*y* axis) vs. fluorescence per cell (*x* axis). The average percentage (± SD) of cells in the G1 phase from three independent cultures is indicated. (**C**) Representative cell viability plates for cultures carrying the pCUP1-V5-CAK1 plasmid, as shown in (**A**, right panel). Cultures were either mock-treated (left plates) or induced with CuSO_4_ (right plates) before plating on media lacking CuSO_4_. Viability dropped from 67% in mock-treated cultures to 19% in CuSO₄-treated cultures (χ^2^ = 8123.4, df=1, *P* < 2.2e-16).Source data are available online for this figure.

The data we described above suggest that a sizable change in T169 phosphorylation in *uORF-0* cells manifests only in very slowly proliferating cells in chemostats (Fig. 4C), despite an increase in Cak1 levels. Consequently, we wondered if the twofold to threefold derepression of Cak1 synthesis in *uORF-0* cells (Fig. 2B) would lead to any phenotypes associated with cell proliferation. We first examined cells proliferating in batch cultures with different carbon or nitrogen sources (Fig. EV2), comparing wild-type cells expressing the 16-residue-long uORF (*uORF-16*) with those that did not (*uORF-0*). There was no significant change in the doubling time (Td) between the two strains, or in budding and cell size (Fig. EV2A), parameters often associated with altered cell cycle kinetics (Polymenis, 2022). Next, we used centrifugal elutriation to obtain highly synchronous early G1 cells from the wild-type (*uORF-16*) and *uORF-0* strains growing with glycerol as the carbon source, and monitored them as they progressed through the cell cycle (Fig. EV2B). In late G1, if budding yeast cells have grown enough in size, they commit to a new round of cell division at a point called START. Passage through START is followed by initiation of DNA replication and appearance of a bud on the cell surface (Hartwell and Unger, 1977; Johnston et al, 1977), which will eventually become the new daughter cell after cytokinesis. A parameter used to gauge START completion is the size at which half of the cells are budded, known as the ‘critical size’ (Polymenis, 2022). We found that *uORF-16* and *uORF-0* cells had the same critical size (Fig. EV2B), arguing that the kinetics of the G1/S transition are unaffected in cells lacking the *CAK1* uORF in otherwise wild-type cells. Hence, although in media with glycerol as the carbon source, the *uORF-0* cells have higher levels of Cak1 and T169 phosphorylation (Fig. 2B), cell cycle progression was not measurably affected (Fig. EV2). To gain insight into phenotypes associated with Cak1 overexpression in general, we used the copper-inducible plasmid driving ectopic *CAK1* expression that we described earlier (Fig. EV1B). In stationary phase cells, where Cak1 levels are typically very low (Fig. 1D), we noticed that cells induced to overexpress *CAK1* were smaller (Fig. EV3A), albeit properly arrested in G1, based on their DNA content (Fig. EV3B). Moreover, *CAK1* overexpression resulted in a three to fourfold decrease in viability in stationary-phase cells (Fig. EV3C). We note that while some commonly used inducible systems (e.g., *GAL*-based) are sensitive to nutrients and the growth status of the cell, induction of *CUP1*-based expression is possible even in cells in the stationary phase (Hottiger et al, 1994).

**Figure 5:**
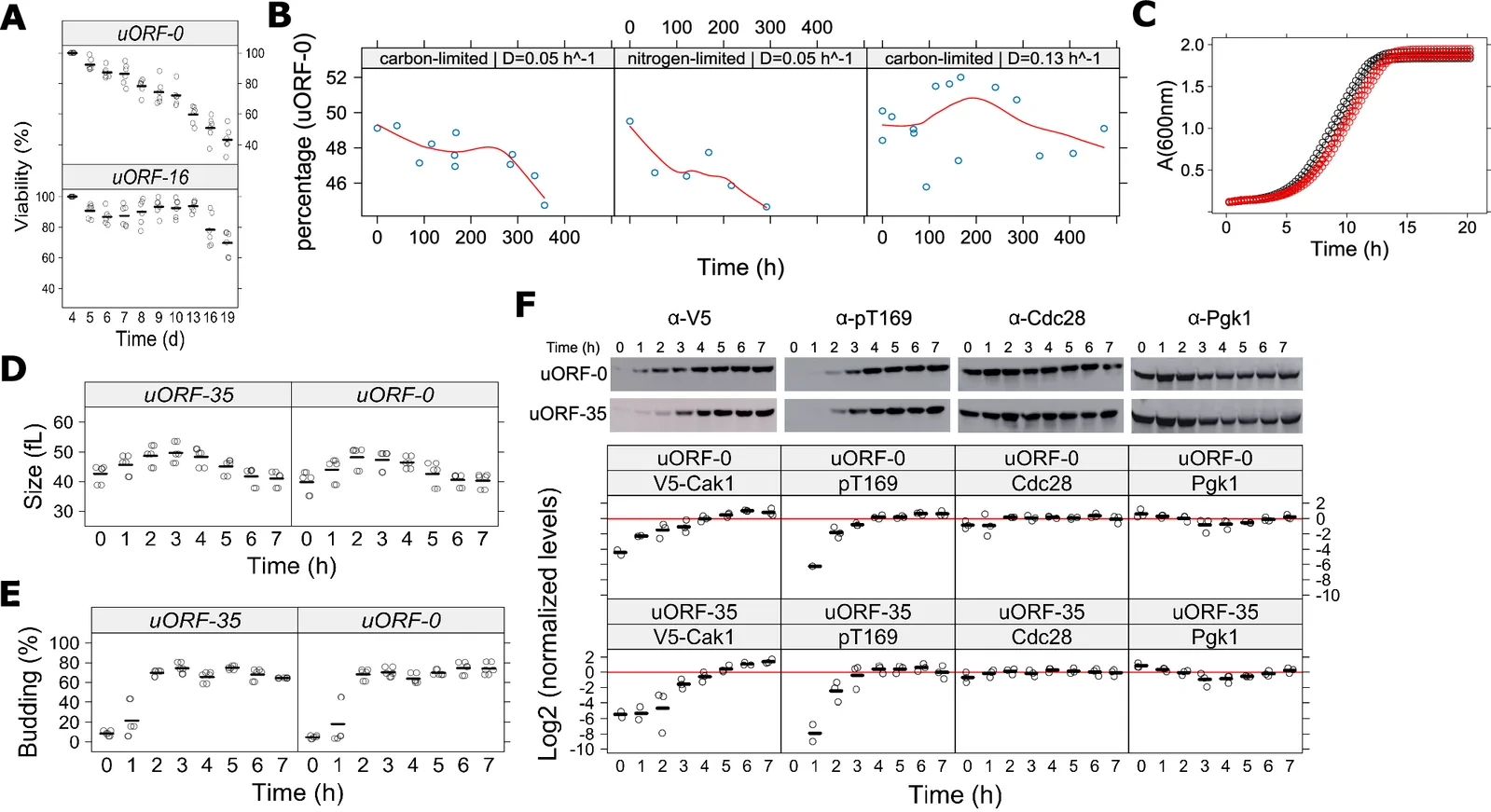
Cells lacking the *CAK1* uORF are outcompeted by wild-type cells in slow-proliferating chemostat cultures, exhibit reduced viability during quiescence, and have a longer lag phase as they exit quiescence. (**A**) Strip plots showing the viability (*y* axis) of quiescent cells over several days (*x* axis), scored as described in the Methods. Cells without the *CAK1* uORF (*uORF-0*, strain MSP257) had lower viability compared to cells with the uORF (*uORF-16*, strain MSP256). Each datapoint represents an independent culture. The reduced viability of uORF-0 cells was statistically significant (*P* = 7.41E-5), based on a mixed-effects model, as described in Methods. The strip plots were drawn as in Fig. 1. (**B**) Plots displaying the percentage of *uORF-0* cells (*y* axis, strain MSP257) against wild-type *uORF-16* cells (strain BY4742) in mixed chemostat cultures over several days (*x* axis) across the indicated dilution rates (*D*) and nutrient limitations. Loess curves are shown in red. (**C**) Growth curves showing the optical density (A_600_, *y* axis) of *uORF-35* (strain MSP258, shown in black) and *uORF-0* cells (strain MSP259, shown in red) over time (*x* axis) following release after 1 week in stationary phase, from three independent experiments in each case. (**D**) Strip plots of cell size (in fL, *y* axis) measured over 7 h (*x* axis) after release from quiescence, using the same strains as in (**C**). (**E**) Budding index representing the percentage of cells with visible buds (*y* axis) over 7 h (*x* axis), using the same strains as in (**C**). (**F**) Immunoblots against V5-Cak1, pT169, Cdc28, and Pgk1 over 7 h (*x* axis) following release after 1 week in the stationary phase of *uORF-35* and *uORF-0* cells (strains MSP258 and MSP259, respectively). For each experiment, the signal for each quantified target was divided by the average intensity for that target in the series across all timepoints on the same gel, and these ratios were then Log2 transformed. These Log2-transformed values are plotted on the *y* axis; the *x* axis indicates the respective growth conditions. The horizontal red line was drawn at the “0” *y* axis value, to facilitate comparisons. Individual data points represent independent replicates, and horizontal bars indicate the mean in each case. Source data are available online for this figure.

Following the above results, arguing that *CAK1* overexpression may decrease the viability of quiescent cells, we next examined the viability of quiescent cells lacking the *CAK1* uORF. Based on their ability to divide and form colonies on solid media, we found that *uORF-0* cells lose viability at an accelerated rate (Fig. 5A, *P* = 7.41E-5, based on mixed-effects regression) compared to their *uORF-16* counterparts. We also measured the competitive fitness of *uORF-0* cells over time in mixed populations (*uORF-0* and *uORF-16* started at equal proportions) in chemostat cultures, as there was a pronounced drop in T169 phosphorylation levels at low dilution rates (see Fig. 4C), concomitant with lower Cak1 levels (Fig. 4A,B). Since *uORF-0* cells carry the *KanMX* resistance marker, their proportion was scored based on their ability to grow on G418-containing solid media. Note that the fitness of cells carrying this marker is unaffected in similar chemostat settings (Baganz et al, 1997). Our results suggest that while at a higher dilution rate (*D* = 0.13 h^−1^; Fig. 5B, middle panel) the fitness of *uORF-0* cells was not lower than that of *uORF-16* cells, it progressively declined at a lower dilution rate (*D* = 0.05 h^−1^), in cultures limited for carbon (Fig. 5B, left panel) or nitrogen (Fig. 5B, right panel).

Lastly, we also monitored *uORF-0* cells as they exited quiescence and reëntered the mitotic cell cycle. After 1 week in the stationary phase, we placed cells in fresh, rich media (YPD) and measured cell numbers, size, budding, and levels of T169 phosphorylation at regular intervals. We found that *uORF-0* cells exhibited a 1–1.5 h delay reaching exponential cell proliferation kinetics (Fig. 5C). Once the culture reached the exponential phase of growth, however, the specific rate of growth was similar to *uORF-35* cells (*P *= 0.286, ANCOVA testing for homogeneity of regression slopes). During the lag phase, the cell size of *uORF-0* cells was slightly smaller than that of uORF-35 cells (Fig. 5D), but they budded with the same kinetics as *uORF-35* cells (Fig. 5E), suggesting that the delay in reaching the exponential phase of growth was likely the result of delayed exit from mitosis during the initial reëntry into mitotic cycling. We also monitored the abundance of V5-Cak1, pT169, Cdc28, and Pgk1 during these experiments by immunoblotting (Fig. 5F). V5-Cak1 levels rose faster in *uORF-0* cells (Fig. 5F, left panels), validating the inhibitory role of the uORF. In line with this rapid Cak1 accumulation, T169 phosphorylation trended higher 1–2 h after refeeding (Fig. 5F, second panels). While our statistical power was limited, Cdk activation was clearly not delayed. Together, these findings indicate that the slower quiescence exit of *uORF-0* cells is driven by a delayed exit from mitosis, rather than impaired Cdk activation.

We conclude that while cells lacking the *CAK1* uORF are indistinguishable from their wild-type counterparts in most proliferative settings under standard laboratory conditions, there are penalties in viability and fitness transitioning between quiescence and mitotic proliferation, and in cultures proliferating very slowly.

### Protein phosphatases counteract changes in cell cycle kinetics in cells lacking the *CAK1* uORF

**Figure EV4:**
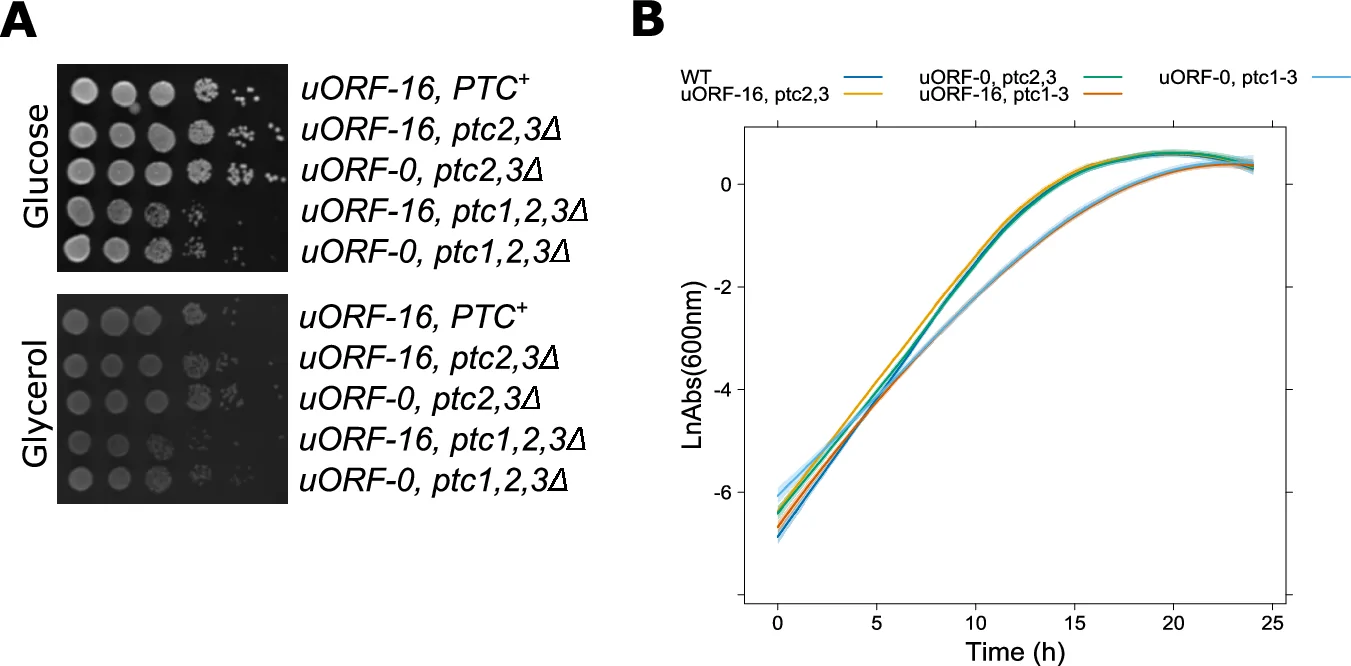
Proliferation of type 2C protein phosphatase mutants with or without the uORF in *CAK1*. (**A**) Growth spot assays to assess the relative growth fitness of the indicated strains (all in the BY4742 background) on glucose (YPD) or glycerol (YPGlycerol) media. Overnight cultures were normalized to an initial cell density, and aliquots were spotted at tenfold serial dilutions. The plates were imaged after 2 (glucose) or 3 (glycerol) days at 30 °C. (**B**) Growth curves of the strains shown in (**A**), cultured in YPD glucose medium. Ln-transformed absorbance values (600 nm) are plotted on the *y* axis against time (*x* axis). Measurements were taken using a microplate reader and corrected for background absorbance of the sterile medium. Data are presented as Loess regression curves; shaded regions represent 95% confidence intervals (*n* = 8 independent replicates per strain).Source data are available online for this figure.

Our data thus far suggest that eliminating the *CAK1* uORF has limited phenotypic consequences in standard laboratory settings, likely because T169 phosphorylation levels are robust against a twofold to threefold change in Cak1 abundance. Since T169 phosphorylation is regulated by both “writer” (Cak1) and “eraser” (phosphatase) enzymes, we next examined the role of protein phosphatases. Type 2C phosphatases dephosphorylate Cdc28 at T169 (Cheng et al, 1999). The seven type 2C phosphatases in budding yeast (Ptc1-7) have overlapping roles with each other and with other phosphatase classes. While cells lacking all seven *PTC* genes are viable in standard conditions, specific mutant combinations are sensitive to various stresses (Sharmin et al, 2014). To test for functional interactions, we first constructed *uORF-0, ptc2,3Δ* triple mutants, because Ptc2 and Ptc3 were reported to dephosphorylate Cdc28 (Cheng et al, 1999). The overall proliferation of *uORF-0, ptc2,3Δ* cells was indistinguishable from that of the *uORF-16, ptc2,3Δ* control on solid media with glucose or glycerol (Fig. EV4A) or in liquid glucose batch cultures (Fig. EV4B). To generate a more sensitized background capable of revealing the consequences of higher Cak1 abundance in *uORF-0* cells, we constructed *uORF-0, ptc1,2,3Δ* quadruple mutants. We chose the additional *PTC1* deletion because Ptc1 is involved in more processes than any other type 2C phosphatase in yeast (Ariño et al, 2019). Indeed, *ptc1,2,3Δ* mutants (with or without the *CAK1* uORF) proliferated more slowly than wild-type or *ptc2,3Δ* cells in glucose- or glycerol-containing media (Fig. EV4).

**Figure 6:**
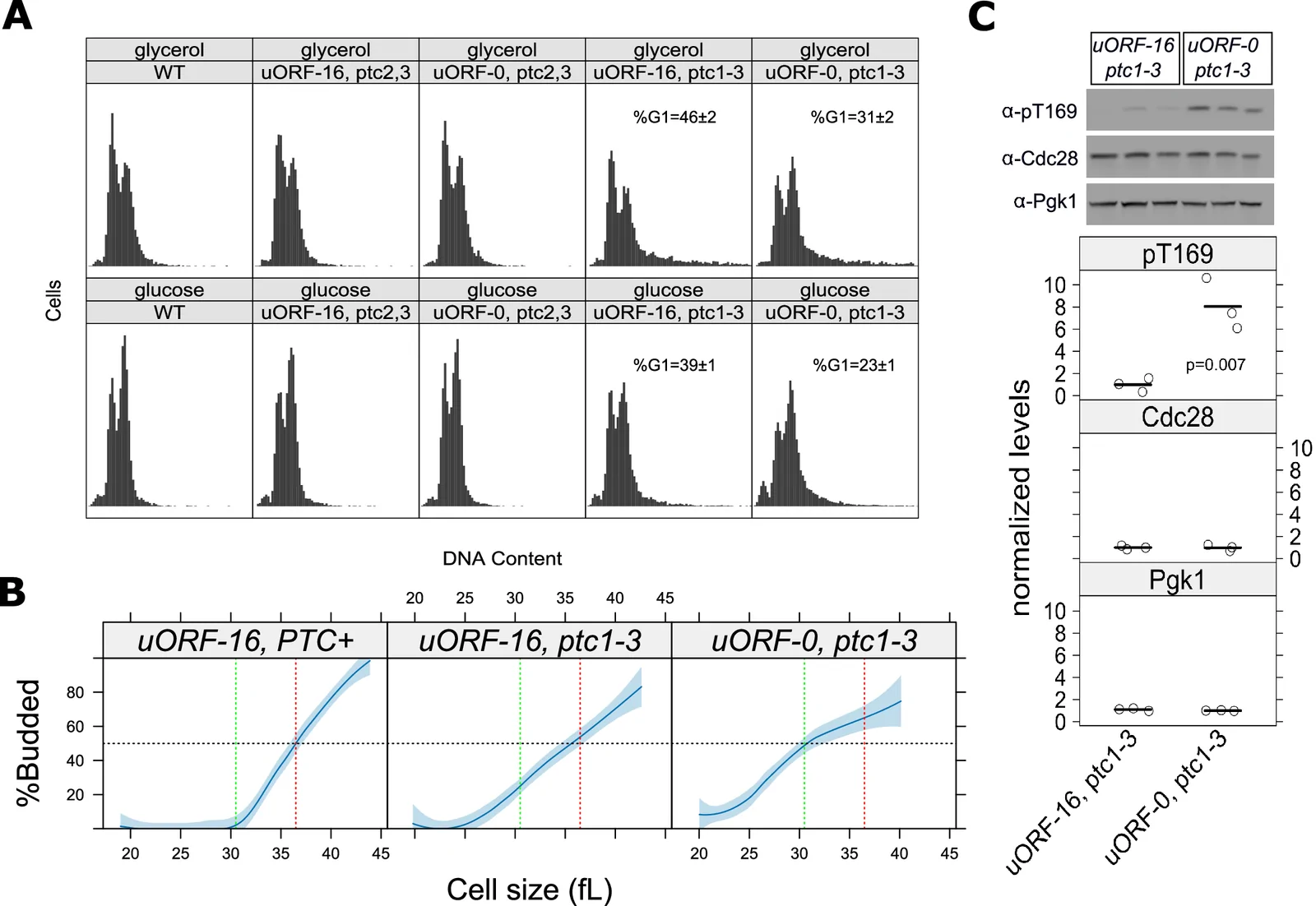
Type 2C protein phosphatases buffer changes in cell cycle kinetics in *uORF-0* cells. (**A**) DNA content analysis of the indicated strains (all in the BY4742 background) in glucose (YPD) or glycerol (YPGlycerol) media growing exponentially in liquid cultures, measured by flow cytometry. Histograms show cell counts (*y* axis) vs. fluorescence per cell (*x* axis). The average percentage (± SD) of cells in the G1 phase from three independent cultures is indicated in select strains. (**B**) Cell cycle progression of elutriated synchronous early G1 cells in YP-glucose. The percentage of budded cells is plotted against cell size (fL) for the indicated strains. The plots show Loess curves with 95% confidence intervals. Red lines indicate the critical size for budding (the size at which 50% of cells have budded) for the wild-type cells (*uORF-16, PTC*^*+*^; strain BY4742), while green lines indicate the critical size of the *uORF-0, ptc1-3Δ* cells (strain HB530). (**C**) (Top) Immunoblots of pT169-Cdc28, Cdc28, and Pgk1 from the indicated strains from three independent cultures grown in glycerol are shown at the top. (Bottom) Strip plots displaying the quantification of the immunoblot data. Band intensities for pT169-Cdc28 and total Cdc28 were first normalized to their respective Pgk1 loading controls. All values, including the standalone Pgk1 signals, were subsequently expressed relative to the mean value of the uORF-16 strain. The *x* axis indicates the respective strains. The indicated *P* value was determined using a two-sample *t* test (Welch’s *t* test), which does not assume equal variance of the two test groups. Source data are available online for this figure.

We next measured the DNA content of these cells proliferating exponentially in liquid batch cultures (Fig. 6A). A noticeable fraction of triple *ptc1-3Δ* cells (with or without the uORF) exhibited a greater than 2N DNA content in both carbon sources, consistent with mitotic defects (Fig. 6A, compare the right two panels to the others). Furthermore, there was a significant decrease (from 39 to 23%, *P* < 0.0001 based on robust bootstrap ANOVA) in the fraction of cells with G1 DNA content in *uORF-0, ptc1,2,3Δ* cells compared to *uORF-16, ptc1,2,3Δ* cells (Fig. 6A, bottom right). A similar effect was evident in glycerol cultures (Fig. 6A, top right). These results indicate that losing the *CAK1* uORF in the absence of “eraser” phosphatases significantly alters cell cycle kinetics.

We then examined synchronous cultures obtained by elutriation (Fig. 6B). Because *ptc1,2,3Δ* cells proliferate slowly and contain many dead cells, they are difficult to elutriate. Even in glucose media, only about two-thirds of the elutriated G1 fraction eventually budded, compared to >90% for wild-type (BY4742) cells (Fig. 6B). Although *uORF-16, ptc1,2,3Δ* cells had the same critical size (~37 fL) as wild-type cells, there were nonetheless more budded smaller cells (Fig. 6B; compare left and middle panels), arguing that the loss of type 2C PPases may promote the initiation of cell division. This effect was greatly exaggerated in *uORF-0, ptc1,2,3Δ* cells, which exhibited a much lower critical size (~30 fL), indicating a pronounced acceleration of START (Fig. 6B, right panel). Given their overall proliferation rate was similar to *uORF-16, ptc1,2,3Δ* cells (Fig. EV4), we conclude that *uORF-0, ptc1,2,3Δ* cells accelerate entry into S phase but delay exit from mitosis, exactly as expected for cells with elevated Cdk activity. Although increased Cdk activity drives the G1/S transition, Cdk must be turned off for cells to complete mitosis. Hence, the elevated T-loop phosphorylation in these cells is expected to impede exit from mitosis. While these phosphatases influence multiple cellular processes, their absence creates a sensitized context that exposes the uORF’s impact on cell cycle progression.

To determine whether these cell cycle defects arise from altered Cdk1 regulation, we measured T169 phosphorylation levels in the *uORF-16, ptc1,2,3Δ* and *uORF-0, ptc1,2,3Δ* cells. Strikingly, the *uORF-0* mutation increased activating phosphorylation in glucose-grown cells, and this effect was amplified in glycerol (Fig. 6C, a sixfold to sevenfold increase, *P* < 0.0001 based on robust bootstrap ANOVA). This surge in T169 phosphorylation explains the accelerated START and delayed mitotic exit observed in this sensitized background (Fig. 6A,B). By unmasking these phenotypes in cells lacking Ptc1-3, these data suggest that the *CAK1* uORF can exert a potent influence on cell cycle progression. These results strongly suggest that protein phosphatases act as a buffer, counteracting the elevated Cak1 abundance caused by the loss of uORF-mediated translational control.

## Discussion

**Figure 7:**
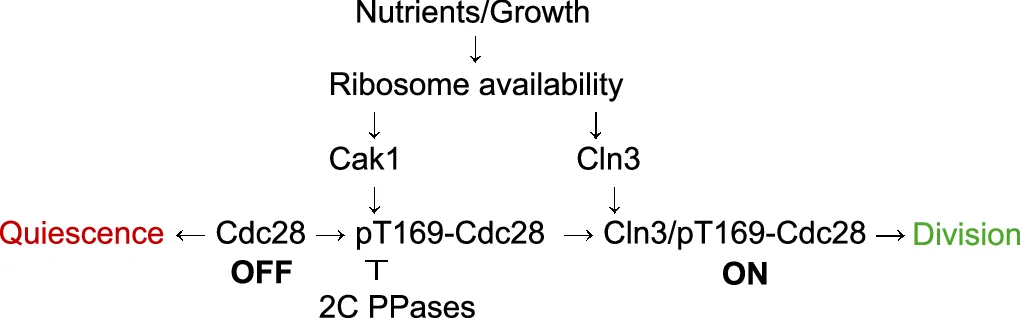
A model of G1/S control through translation. The uORF-mediated translational control of the transcripts encoding two core Cdk regulators, Cak1 and Cln3, couples cell division to ribosome availability, which is known to be a linear function of growth rate (Warner and Gorenstein, 1978; Waldron and Lacroute, 1975; Ju and Warner, 1994; Metzl-Raz et al, 2017; Gao et al, 2025; Xia et al, 2022). It is also assumed that the activity of Cln3/Cdc28 requires T169 phosphorylation.

The results we presented resolve a long-standing question regarding the regulation of CAK activity by demonstrating growth-dependent control of Cak1 synthesis and T169 phosphorylation. We identified the molecular mechanism underpinning this control as a cis element (the uORF) within the *CAK1* transcript, rendering Cak1 synthesis disproportionately sensitive to a central aspect of cell growth: ribosome availability. This uORF-mediated regulatory logic is consistent with the classic kinetic model of translation (Lodish, 1974; Mills and Green, 2017), where ribosomes initiating at a uORF bypass the downstream start codon of the main ORF. Because yeast ribosome content scales with growth rate (Warner and Gorenstein, 1978; Waldron and Lacroute, 1975; Ju and Warner, 1994; Metzl-Raz et al, 2017; Gao et al, 2025; Xia et al, 2022), the reduced ribosome availability in nutrient-poor conditions or at low chemostat dilution rates disfavors translation of the *CAK1* ORF. Consequently, the cell’s bulk biosynthetic capacity is directly linked to the activation of its core cell cycle machinery (Fig. 7).

We previously reported a similar uORF-mediated mechanism controlling the early G1 cyclin Cln3 (Polymenis and Schmidt, 1997; Blank et al, 2018). Hence, yeast cells couple the levels of two direct Cdk activators, Cak1 and Cln3, to protein synthesis capacity, despite their vastly different protein stabilities (Fig. EV1). While Cak1 is a very stable protein (see Fig. EV1 and (Christiano et al, 2014; Kaldis et al, 1998a)), Cln3 is highly unstable (Fig. EV1 and (Yaglom et al, 1995; Cross and Blake, 1993; Tyers et al, 1992)). This kinetic difference likely reflects distinct physiological roles. Several reports have questioned the initial assumption (Tyers et al, 1993) that Cln3 abundance does not vary in the cell cycle. Instead, Cln3 levels (Litsios et al, 2019; Sommer et al, 2021; Thorburn et al, 2013; Zapata et al, 2014; Landry et al, 2012; Litsios et al, 2022) and protein synthesis rates (Takhaveev et al, 2023; Litsios et al, 2024) appear to be dynamic in the cell cycle. Cln3’s instability makes it better suited to act quickly as a real-time indicator of protein synthesis capacity. In contrast, because Cak1 is not rapidly degraded, its uORF-mediated repression slowly lowers total Cdk activity, not just G1/Cdk, resetting the cell to a much lower proliferative baseline to optimize survival during prolonged nutrient limitation.

Cycling cells were impervious to changes in Cak1 levels in most laboratory contexts. This was the result of counteracting type 2C protein phosphatases, which are apparently quite efficient in buffering T169 phosphorylation against changes in Cak1 levels mediated by the uORF mechanism we described (Fig. 6). Our data also suggest that translational control of *CAK1* may promote survival in the wild (Figs. 4 and 5). In natural environments, where limited and changing resources are the norm, the uORF in *CAK1* would promote fitness by efficiently inhibiting Cak1 synthesis and cell division when nutrient availability drops significantly. Furthermore, while our emphasis here was on the T-loop phosphorylation of Cdc28, the only essential function of Cak1, it is known that Cak1 targets additional proteins. Hence, it is possible that the growth-dependent regulation of Cak1 through the uORF affects other processes as well, coupling them to cellular biosynthesis, contributing to the phenotypes we observed.

Our data suggest the relationship between Cak1 abundance and the level of T169-Cdc28 phosphorylation is non-linear. While eliminating the uORF restored Cak1 levels in the stationary phase, it failed to restore T169 phosphorylation (Fig. 2). In chemostat cultures, T169 phosphorylation dropped precipitously at low dilution rates even when Cak1 levels were stabilized or increased by the absence of the uORF (Fig. 4). These results suggest that Cak1 abundance is not the only determinant of T-loop phosphorylation. It is highly likely that “eraser” mechanisms, such as the activity of type 2 C protein phosphatases (Ptcs), become dominant in poor growth conditions. Our finding that cells lacking several PTC genes become significantly more sensitive to Cak1 levels supports this buffering role for phosphatases.

Other processes may also account for the non-linear relation between Cak1 levels and T169 phosphorylation. In the stationary phase or in chemostats at low dilution rates, cyclins will be absent or at exceedingly low levels, and most of the Cdk would exist as a monomer. Although Cak1 targets the Cdk monomer (Kaldis et al, 1998b; Tsakraklides and Solomon, 2002), in certain cases, it can preferentially target Cdk-Cyclin dimers (López-Hernández and Vázquez-Ramos, 2023). In poor growth conditions, the Cdk monomer may be a poorer substrate for Cak1 or more susceptible to dephosphorylation by phosphatases. In such a scenario, the uORF-mediated control of Cak1 synthesis contributes to the maximum level of Cdk activation. Phosphatases and cyclin availability may provide an additional fail-safe to ensure T169 dephosphorylation when growth is unfavorable.

The data we described contribute to a unifying view of CAK across all eukaryotes. Observations in animal cells, such as the higher threshold of T-loop Cdk phosphorylation required for the G1/S transition in response to extracellular signals and the heightened involvement of counteracting phosphatases at that proliferative transition (Schachter et al, 2013; Blank et al, 2025), point to a general and conserved role for CAK in safeguarding cells before they commit to a new round of division. More generally, recent studies in mammals also suggest that Cdk T-loop phosphorylations are subject to greater regulatory control than previously assumed (Schachter et al, 2013; Kirova et al, 2022). Lastly, human CAK has been identified as a promising target for cancer therapy, and several CAK inhibitors are being evaluated in clinical trials (NCT04247126, NCT03134638, NCT05394103), underscoring the interest and significance of CAK-mediated control of cell proliferation.

## Methods

**Table:** Reagents and tools table

Reagent/resource	Reference or source	Identifier or catalog number
**Experimental models**		
*S. cerevisiae*, X2180-5A (A segregant of the reference strain S288C (MATα SUC2 mal mel gal2 CUP1)	Yeast Genetic Stock Center - R.K. Mortimer	
*S. cerevisiae*, X2180-5A (ChVI:79250::KanMX6 uORF-35 V5-CAK1, X2180-5A otherwise)	This study (MSP258)	
*S. cerevisiae*, X2180-5A (ChVI:79250::KanMX6 uORF-0 V5-CAK1, X2180-5A otherwise)	This study (MSP259)	
*S. cerevisiae*, X2180-5A (ChVI:79250::KanMX6 uORF-3 V5-CAK1, X2180-5A otherwise)	This study (MSP268)	
*S. cerevisiae*, BY4741 (MATa his3Δ1 leu2Δ0 met15Δ0 ura3Δ0)	Giaever et al, 2002	RRID:SCR_003093
*S. cerevisiae*, BY4741 (CDC28-TAP HIS3MX6, BY4741 otherwise)	YSC1178-202229953	Dharmacon
*S. cerevisiae*, BY4742 (MATα his3Δ1 leu2Δ0 lys2Δ0 ura3Δ0)	Giaever et al, 2002	RRID:SCR_003093
*S. cerevisiae*, BY4743 (MATa/α his3Δ1/his3Δ1 leu2Δ0/leu2Δ0 LYS2/lys2Δ0 met15Δ0/MET15 ura3Δ0/ura3Δ0)	Giaever et al, 2002	RRID:SCR_003093
*S. cerevisiae*, BY4742 (ChVI:79250::KanMX6 uORF-16 CAK1, BY4742 otherwise)	This study (MSP256)	
*S. cerevisiae*, BY4742 (ChVI:79250::KanMX6 uORF-0 CAK1, BY4742 otherwise)	This study (MSP257)	
*S. cerevisiae*, BY4743 (ChVI:79250::KanMX6 uORF-3 CAK1, BY4742 otherwise)	This study (MSP267)	
*S. cerevisiae*, BY4742 (ptc2Δ::URA3, BY4742 otherwise)	This study (MSP269)	
*S. cerevisiae*, BY4743 (ptc3Δ::KanMX/ptc3Δ::KanMX, BY4743 otherwise)	Giaever et al, 2002	33082
*S. cerevisiae*, BY4742 (ChVI:79250::KanMX6 uORF-16 CAK1 ptc2Δ::URA3 ptc3Δ::KanMX, BY4742 otherwise)	This study (HB509)	
*S. cerevisiae*, BY4742 (ChVI:79250::KanMX6 uORF-0 CAK1 ptc2Δ::URA3 ptc3Δ::KanMX, BY4742 otherwise)	This study (HB505)	
*S. cerevisiae*, BY4742 (ChVI:79250::KanMX6 uORF-16 CAK1 ptc1Δ::HIS3 ptc2Δ::URA3 ptc3Δ::KanMX, BY4742 otherwise)	This study (HB531)	
*S. cerevisiae*, BY4742 (ChVI:79250::KanMX6 uORF-0 CAK1 ptc1Δ::HIS3 ptc2Δ::URA3 ptc3Δ::KanMX, BY4742 otherwise)	This study (HB530)	
*S. cerevisiae*, BY4741 (ptc1Δ::HIS3, BY4741 otherwise)	(González-Rubio et al)	
*S. cerevisiae*, BY4741 (CLN3-13MYC::TRP1 WHI5-TAP::HIS3, BY4741 otherwise)	This study (HB490)	
*E. coli*, DH5alpha	New England Biolabs	C2987H
**Recombinant DNA**		
pFA6a-KanMX6	PMID: 9717241	
MoClo-YTK plasmid kit	John Dueber	Addgene_1000000061
pV5CAK1_ENTRY (Synthetic DNA fragment containing the V5-CAK1 allele flanked by adapter sequences for multi-part cloning)	This study	
YEp-pCUP1-V5-CAK1 (Plasmid for inducible expression of V5-CAK1)	This study	
uORF-mTurquoise2_ENTRY (Synthetic DNA fragment containing chrVI:79401-79196 fused to the mTurquoise2 ORF flanked by adapter sequences for multi-part cloning)	This study	
CAK1-mRuby2_ENTRY (Synthetic DNA fragment containing chrVI:79401-79159 fused to the mRuby2 ORF flanked by adapter sequences for multi-part cloning)	This study	
uORFm-CAK1-mRuby2_ENTRY (Synthetic DNA fragment containing chrVI:79401-79159 fused to the mRuby2 ORF, but with the uORF start codon mutated to AAG, and flanked by adapter sequences for multi-part cloning)	This study	
**Antibodies**		
Anti-Pgk1p antibody, mouse monoclonal	ThermoFisher	459250
Alexa Fluor® 488 AffiniPure, goat anti-mouse IgG (H + L)	Jackson Immunoresearch Laboratories	115-545-003
Anti-Mouse IgG (H + L), goat HRP-conjugated	ThermoFisher	31431
Anti-S6 phospho Ser235/236, rabbit monoclonal	Cell Signaling	4858
Anti-Rabbit IgG (H + L), goat HRP-conjugated	ThermoFisher	31466
Anti-Cdc28p, mouse monoclonal	Santa Cruz Biotechnologies	sc-515762
Anti-pT169 Cdc28p, rabbit polyclonal	This study, custom-made (Biomatik, Ontario, Canada)	
**Oligonucleotides and other sequence-based reagents**		
VI:79250-79325-F1 (AAAGACCCCCTGTATAGAAAATATGTATCTTAGTGGAAAATGTGATTTGACTATTTAAGTTATAGAAGTAGTTTCGGATCCCCGGGTTAATTAA)	This study	
VI:79159-79249-R1 (ATCCAGTTTCATTAAGATGGGCGTGAATTTTGTTGCCTCTCTTCTTCATCTGGCCTCTTTGCTTCTATGAGACTTCTGTCGTTTTCCTTTTTTTAGGATCGAATTCGAGCTCGTTTAAAC)	This study	
VI:79600-FWD (AGATGCTCAGTGGTCTACGC)	This study	
VI:78815-REV (GCAACAATTGGGATACTGGGA)	This study	
V5-CAK1-REV (AGCAGTTCTAGTAGATTTGACCAACTGACAGTGTGTAATGTCTATACTATCCAGTTTCGTAGAATCGAGACCGAGGAGAGGGTTAGGGATAGGCTTACCCATTAAGATGGGCGTGAATTTTGTTG)	This study	
VI:79159-uORFm-R1 (ATCCAGTTTCATTAAGATGGGCGTGAATTTTGTTGCCTCTCTTCTTCTTCTGGCCTCTTTGCTTCTATGAGACTTCTGTCGTTTTCCTTTTTTTAGGATCGAATTCGAGCTCGTTTAAAC)	This study	
uORFm1-3-REV (GGCTTACCCATATGAGACCATTAAGATGGGCGTGAATTTTGTTGCCCTACTTCTTCATCTGGCCTCTTTGCTTCTATGAGACTTCTGTCG)	This study	
AmpR-FWD (GACAGATCGCTGAGATAGG)	This study	
pBR322ori-FWD (GGGGGAAACGCCTGGTATCTTT)	This study	
VI:79398-FWD (ACTAGAATGGTTGAAACGTG)	This study	
mTurquoise2-FWD (GCTGCTGGTATTACCTTGGG)	This study	
mRuby2-FWD (AAGGGATCCTGAATCCTGAG)	This study	
CAK1-FAM-probe	Sc04106277_s1, Cat#4448892	Thermo Fisher Scientific
UBC6-VIC-probe	Sc04118595_s1, Cat# 4351372	Thermo Fisher Scientific
V:337690-FWD (GCTGATAAATGGAAGGGGATGG)	This study	
V:335594-REV (ATCCGCGCTGTCTACCTTAG)	This study	
PTC2-URA3-FWD (ATCCCTAGGATTTTGACTATTCCATTGTTGTATAAAATATAGAGAACCAGAAAAAGAAAAATGTCGAAAGCTACATATAAGG)	This study	
PTC2-URA3-REV (TCTTTCTGTTCGTTCGGTTCGTATATAGGTATGTATATATAATGAAGGATGGAAGATCCTTTAGTTTTGCTGGCCGCATCTTC)	This study	
II:113444-FWD (TGTTACTCCGCTGTCAACAAG)	This study	
II:114791-REV (TGGTGGTTATTGCGAACACT)	This study	
KanMX-REV (GCGCATCAACAATATTTTCACCT)	This study	
**Chemicals, enzymes, and other reagents**		
Dextrose	Millipore-Sigma	D9434
Glycerol	Millipore-Sigma	G5516
Zymolyase 20 T	MP biomedicals	SKU 08320921
Tris (hydroxymethyl) aminomethane	Millipore-Sigma	252859
Tris base	Roche	TRIS-RO
Sodium chloride	Millipore-Sigma	S7653
Magnesium chloride hexahydrate	USP	1374248
DTT	Millipore-Sigma	D0632
Triton™ X-100	Millipore-Sigma	T8787
Phosphate-buffered saline (PBS)	Millipore-Sigma	P4417
Sodium dodecyl sulfate	Millipore-Sigma	L3771
Glycine	Millipore-Sigma	G8898
Sucrose	Millipore-Sigma	S0389
Ammonium sulfate	Millipore-Sigma	A4915
Cycloheximide	Millipore-Sigma	C1988
Sodium Azide	Millipore-Sigma	S2002
Copper(II) sulfate	Millipore-Sigma	C1297
Ampicillin sodium salt	Millipore-Sigma	A9518
Chloramphenicol	Millipore-Sigma	C0378
Kanamycin sulfate	Millipore-Sigma	K1377
G 418 disulfate salt	Millipore-Sigma	G1279
SYTOX™ Green Nucleic Acid Stain - 5 mM Solution in DMSO	ThermoFisher	S7020
One step RT-ddPCR Advanced kit for probes	Bio-Rad	1864021
BioTrace™ NT nitrocellulose	Pall Corporation	66485
Immobilon®-FL membrane	Millipore-Sigma	IPFL07810
Mini-PROTEAN® TGX Stain-Free™ Gels, 4-15%	Bio-Rad	4568086
30% Acrylamide/Bis Solution, 29:1	Bio-Rad	1610156
SuperSignal™ West Pico PLUS Chemiluminescent Substrate	ThermoFisher	34579
IgG Sepharose™ 6 Fast Flow	Millipore-Sigma	GE17-0969-01
BsaI-HF®v2	New England Biolabs	R3733S
T4 DNA Ligase	New England Biolabs	M0202S
Yeast extract	Millipore-Sigma	Y1625
Peptone	Millipore-Sigma	P5905
Tryptone	Millipore-Sigma	T7293
Yeast Nitrogen Base Without Amino Acids and Ammonium Sulfate	Millipore-Sigma	Y1251
Glass beads	Scientific Industries	SI-BG05

### Strains and media

All *S. cerevisiae* strains used in this study are listed in the Reagents and Tools Table.

#### Culture conditions

Unless indicated otherwise, the cells were cultured on the standard, rich, YPD medium (1% ^w^/_v_ yeast extract, 2% ^w^/_v_ peptone, 2% ^w^/_v_ dextrose), at 30 °C. Throughout the manuscript, glucose refers to dextrose (D-glucose). In some experiments, as indicated, glycerol (2% ^w^/_v_) replaced dextrose as the carbon source. Synthetic minimal media (SMM) contained 0.17% ^w^/_v_ yeast nitrogen base without amino acids and ammonium sulfate, 0.5% ^w^/_v_ nitrogen source (ammonium sulfate or proline, as indicated), 2% ^w^/_v_ dextrose, and were supplemented as needed for auxotrophies (Kaiser et al, 1994). For experiments involving the inducible expression of *CAK1*, the media were standard synthetic complete, lacking uracil (SC-Ura) (Kaiser et al, 1994), with 2% ^w^/_v_ dextrose or glycerol, as indicated, and the cells were cultured at 30 °C. For the experiments using the reporter plasmids, the media were also SC, but lacking both uracil and leucine. For plasmid construction and propagation in *E. coli* bacteria, cells were cultured on the standard lysogeny broth media (0.5% ^w^/_v_ yeast extract, 1% ^w^/_v_ tryptone, 1% ^w^/_v_ sodium chloride), at 37 °C.

#### Chemostat experiments

Chemostat experiments were conducted using a BioFlo 110 bioreactor system (New Brunswick Scientific, Edison, NJ) with a working volume of 880 mL. To monitor the abundance of various proteins and T169 phosphorylation, we used prototrophic strains with agitation set at 800 rpm for these experiments, at room temperature (Blank et al, 2018; Henry et al, 2010; Guo et al, 2004). The carbon-limited media were 0.17% ^w^/_v_ yeast nitrogen base without amino acids and ammonium sulfate, 0.5% ^w^/_v_ ammonium sulfate, and 0.08% ^w^/_v_ dextrose. The nitrogen-limited media were 0.17% ^w^/_v_ yeast nitrogen base without amino acids and ammonium sulfate, 0.002% ^w^/_v_ ammonium sulfate, and 2% ^w^/_v_ dextrose. The dilution rate was adjusted as indicated for each experiment. The cell density remained >1E + 07 cells/mL and did not vary more than threefold between the lowest and highest dilution rates. For experiments with auxotrophic strains (Fig. 5), the media were supplemented as needed.

#### Strain construction

**Figure EV5:**
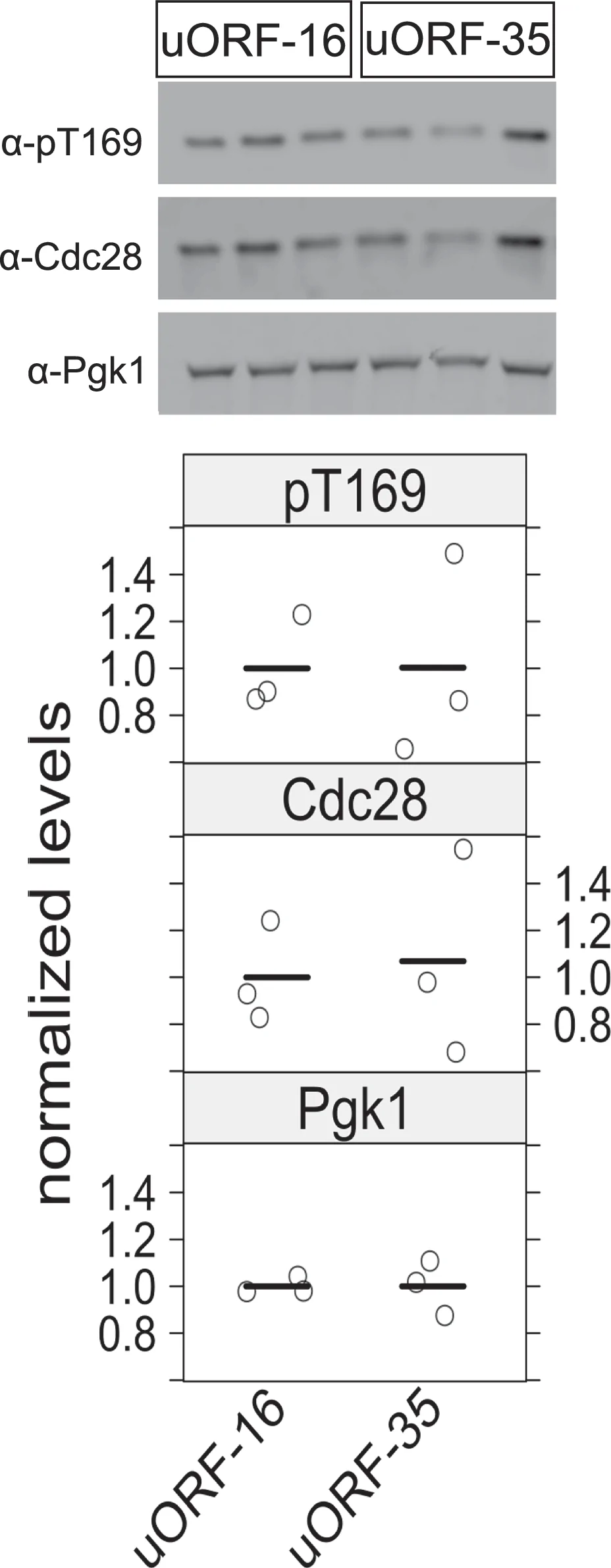
The levels of Cdk T169 phosphorylation are the same in uORF-16 and uORF-35 cells. Immunoblots of pT169-Cdc28, Cdc28, and Pgk1 from the indicated strains (both in the X2180-5A background) from three independent cultures grown in glucose are shown at the top. The strip plot graphs below display the quantification of each target from the three independent experiments shown on each gel. Band intensities were quantified, and their normalized values were calculated as in Fig. 6C for each target protein and plotted on the *y* axis; the *x* axis indicates the respective strains.Source data are available online for this figure.

To monitor Cak1 protein abundance, we initially constructed strains carrying an allele encoding a TAP-tag fused to *CAK1* at the C-terminus. However, these strains lost viability after a few generations, and the cells had elongated, sausage-like morphology, indicative of mitotic defects. Consequently, we then constructed strains carrying N-terminal V5-tagged *CAK1* alleles, expressing *CAK1* from the endogenous chromosomal locus. We first inserted a *KanMX6* cassette upstream, at position ChrVI:79250, with PCR-mediated methodology (Longtine et al, 1998), using primers VI:79250-79325-F1 and VI:79159-79249-R1 (see Reagents and Tools Table), and plasmid pFA6a-KanMX6 as a template. We introduced the PCR product into strain BY4742 to generate strain MSP256. The *KanMX6* insertion was verified by PCR, using primers VI:79600-FWD and VI:78815-REV. Then, we used genomic DNA from strain MSP256 as a template in a PCR reaction with primers V5-CAK1-REV and VI:79600-FWD. The PCR product was used to transform the prototrophic strain X2180-5A. The resulting strain (MSP258; denoted as *uORF-35, V5-CAK1* throughout the manuscript) expressed a protein of the expected size for V5-Cak1, based on immunoblots, and the genomic area was also sequenced to verify the absence of any introduced mutations. Note that the uORF normally encodes a 16-residue peptide (Fig. 2, top; denoted as uORF-16). By inserting the V5 sequences at the *CAK1* locus, the uORF was extended to 35 residues (Fig. 2, second from top; denoted as uORF-35). The doubling time and the percentage of budded cells between strains X2180-5A (*uORF-16*, *CAK1*) and MSP258 (*ChrVI:79250::KanMX6*, *uORF-35*, *V5*-*CAK1*, X2180-5A otherwise) were indistinguishable, in glucose and glycerol media. Furthermore, *uORF-35* cells (strain MSP258) displayed T169 phosphorylation levels identical to those of *uORF-16* cells (Fig. EV5), and their viability was the same after 10 d in the stationary phase (93.5% ± 6.2 vs. 92.6% ± 6.0, respectively; see Source Data for Fig. 5A).

To mutate the start codon of the uORF (AUG → AAG substitution) upstream of the *CAK1* ORF, we used primers VI:79250-79325-F1 and VI:79159-uORFm-R1 in a PCR reaction with plasmid pFA6a-kanMX6 as a template. The PCR product was then used to transform strain BY4742, generating strain MSP257 (*ChrVI:79250::KanMX6*, *uORF-0*, *CAK1*, BY4742 otherwise). The *KanMX6* insertion was verified by PCR, and the introduction of the mutation was confirmed by sequencing. As above, we then used genomic DNA from strain MSP257 as a template in a PCR reaction with primers V5-CAK1-REV and VI:79600-FWD. The PCR product was used to transform the prototrophic strain X2180-5A, generating strain MSP259 (Fig. 2, second from bottom; denoted as *uORF-0*, *V5*-*CAK1*), which was verified as described above for strain MSP258 (*uORF-35*, *V5*-*CAK1*). We also generated a strain carrying a truncated uORF, encoding only its first three amino acids. We used primers VI:79600-FWD and uORFm1-3-REV in a PCR reaction with genomic DNA from strain MSP256 as a template. The PCR product was used to transform strain X2180-5A to generate strain MSP267. Introduction of the desired mutation was verified by sequencing as described above. To epitope-tag the *CAK1* allele in this strain background, we then used genomic DNA of strain MSP267 as a template in a PCR reaction with primers V5-CAK1-REV and VI:79600-FWD. The PCR product was used to transform the prototrophic strain X2180-5A, generating strain MSP268 (*uORF-3*, *V5*-*CAK1*), verified by sequencing and immunoblotting to ensure that it expresses V5-Cak1 as expected.

To generate a strain lacking *PTC2* (encoding a type 2C protein phosphatase), we amplified the *URA3* ORF using primers PTC2-URA3-FWD and PTC2-URA3-REV and genomic DNA from strain X2180-5A. We then used the resulting PCR product to transform strain BY4742, yielding a *ptc2Δ::URA3* derivative (strain MSP269). We verified the correct replacement of *PTC2* with *URA3* using primers V:337690-FWD and V:335594-REV and the genomic DNA of each putative transformant as a template. A haploid strain (HB499) lacking *PTC3* was obtained by sporulation of the homozygous diploid *ptc3Δ::KanMX/ptc3Δ::KanMX* strain 33082. The double *ptc2Δ::URA3 ptc3Δ::KanMX* haploid strain (HB509) was a segregant of the cross between HB499 and MSP256. The *ChrVI:79250::KanMX6, uORF-0, ptc2,3Δ* haploid strain (HB505) was a segregant of the cross between HB509 and MSP257. Lastly, we crossed a *ptc1Δ::HIS3* (BY4741 otherwise) strain described elsewhere (González-Rubio et al, 2023) with strain HB509 or HB505. Segregants of these crosses were selected for being triple *ptc1-3Δ* mutants without (HB531) or with the *uORF-0* mutation (HB530), respectively, by co-segregation of the *KanMX*^*r*^, *URA*^*+*^, and *HIS*^*+*^ markers. Using genomic DNA from these strains in PCR reactions with primers VI:78815-REV and VI:79398-FWD, we validated the presence of the uORF-0 mutation (strain HB531) or not (strain HB530) by sequencing the PCR product with the VI:78815-REV primer. Since the *PTC3* deletion was also marked with the *KanMX* marker, we confirmed that these strains were indeed *ptc3Δ::KanMX* through PCR, with primers II:113444-FWD and KanMX-REV, and genomic DNA as template. We also confirmed that they had no wild-type *PTC3* allele, in a PCR reaction with primers II:113444-FWD and II:114791-REV, and genomic DNA as template.

To measure the half-life of Cln3 (Fig. EV1), we used strain HB490, a derivative of HB94 (Blank et al, 2018). Because HB94 carries the *CLN3-13MYC::TRP1* allele but is a segregant from a mixed W303/BY background, we backcrossed it twice to the BY background to generate HB490.

### Plasmids

All enzymes used in plasmid construction were from New England Biolabs. To generate the plasmid driving copper-dependent expression of *V5-CAK1*, we used the single-pot approach and the plasmids included in the MoClo kit (Lee et al, 2015), as detailed below. A synthetic DNA fragment was made (Integrated DNA Technologies, Coralville, IA) containing the V5*-CAK1* allele flanked with adapter sequences compatible with the MoClo multi-part assembly. The V5*-CAK1* DNA fragment was digested with BsaI, electrophoresed through an agarose gel (1% ^w^/_v_), and purified. It was then combined with plasmids YTK002, YTK067, YTK031, YTK063, YTK074, YTK082, and YTK083 in a ligase reaction containing T4 ligase together with the BsaI restriction endonuclease. The reaction was carried out in a thermocycler at 37 °C for 20 m; (37 °C for 5 m, 16 °C for 5 m) for 50 cycles; 60 °C for 5 m; 80 °C for 10 m. An aliquot was used to transform *E. coli* (high-efficiency DH5alpha cells; New England Biolabs). Transformants were selected and verified to contain the high-copy (2 micron; 2 μ) YEp-pCUP1-V5-CAK1 plasmid, where the copper-inducible *pCUP1* promoter drives V5*-CAK1* expression, the terminator is *tADH1*, the yeast selection marker is *URA3*, and AmpR-ColE1 is used for bacterial selection and propagation. The plasmid was sequenced with primers pBR322ori-FWD and AmpR-FWD (see Reagents and Tools Table). Expression of a protein of the expected size upon induction with CuSO_4_ (0.5 mM) was confirmed by immunoblotting for V5-Cak1 (see Fig. EV1B).

We also followed the same single-pot approach with reagents from the MoClo kit to generate several reporter constructs. Synthetic DNA inserts were made (Integrated DNA Technologies, Coralville, IA) containing *CAK1* upstream sequences (starting at position chrVI:79401) fused to sequences encoding fluorescent reporter proteins and flanked with adapter sequences compatible with the MoClo multi-part assembly. These inserts were:

- *uORF*::mTurquoise2: chrVI:79401-79196 fused to the mTurquoise2 ORF.
- *uORF* + *CAK1*::mRuby2: chrVI:79401-79159 fused to the mRuby2 ORF.
- *uORF-0 CAK1*::mRuby2 (chrVI:79401-79159 fused to the mRuby2 ORF, but with the uORF start codon mutated to AAG).

The plasmids containing these synthetic insert DNA fragments were digested with BsaI, electrophoresed through an agarose gel (1% ^w^/_v_), and the inserts isolated. The inserts were then combined with plasmids YTK002, YTK067, YTK031, YTK063, YTK074 (for the mRuby2 reporters) or YTK075 (for the mTurquoise2 reporter), YTK082, and YTK083 in a ligase reaction containing T4 ligase together with the BsaI restriction endonuclease. The reactions were performed as above and used to transform *E. coli* and isolate the relevant plasmids. The plasmids were verified by sequencing as above, and with additional oligonucleotides that correspond to their insert sequences (VI:79398-FWD, mTurquoise2-FWD, and mRuby2-FWD; see Reagents and Tools Table). The plasmids contained the *tADH1* terminator, the 2 micron (2μ) element for high-copy propagation in yeast cells, and the AmpR-ColE1 elements for bacterial selection and propagation. The yeast selection marker is *URA3* for the mRuby2 reporters, and *LEU2* for the mTurquoise2 one.

### Cdc28-TAP immunoprecipitation and mass spectrometry for T169-Cdc28 phosphorylation

To detect the T169 phosphorylation by mass spectrometry (Fig. 1), we used a strain (BY4741 background) carrying a *CDC28-TAP* allele expressed from its endogenous chromosomal location (Ghaemmaghami et al, 2003) (see Reagents and Tools Table).

Overnight cultures growing in rich undefined media with glucose (YPDextrose) or glycerol (YPGlycerol) as the carbon source were diluted 1:100 into fresh 1 L cultures and incubated at 30 °C for several hours until they reached a cell density of ~2-5E + 07 cells/mL in YPD, or ~8E + 06 to 1E + 07 cells/mL in YPGlycerol. The cultures were quenched with sodium azide (at 0.1% final concentration) and cycloheximide (100 μg/mL). The cells were collected by centrifugation, and the pellets were stored at −80 °C until needed.

For immunoprecipitation, the cell pellets were washed in ice-cold lysis/IP buffer (10 mM Tris-HCl, pH = 8.0, 150 mM NaCl), resuspended at ~1E + 09 cells per reaction in 0.6 mL of lysis buffer, containing protease and phosphatase inhibitor cocktails, and kept on ice. Glass beads (~0.35 mL) were then added. The cells were broken in eight cycles of vortexing at the maximum speed for 30 s, followed by placement on ice for 30 s. The samples were centrifuged at 5000× *g* for 5 m, and the supernatants were collected and placed in fresh tubes on ice. To each reaction were added 50 μL of IgG-Sepharose-6 fast-flow beads (pre-washed with lysis buffer), and incubated for 2 h on a rotisserie mixer at 4 °C. The beads were collected by centrifugation at 500× *g* for 1 m, washed twice with lysis buffer and twice with 10 mM ammonium bicarbonate buffer, prepared fresh on the day of the experiment. The beads were resuspended in 75 μL of ammonium bicarbonate buffer and shipped at 4 °C to the Proteomics Core Facility of the Genome Center, University of California, Davis.

Protein samples on beads were washed four times with 0.2 mL of 50 mM Triethyl ammonium bicarbonate (TEAB) with a 20 m shake time at 4 °C in between each wash. Roughly 2.5 μg of trypsin was added to the bead and TEAB mixture, and the samples were digested overnight at 800 rpm shake speed, at 37 °C, followed by a boost addition of trypsin using the same wt/wt ratios for overnight digestion at 37 °C. To stop the digestion, the reaction mixture was acidified with 1% trifluoroacetic acid (TFA). The supernatant containing the tryptic peptides was dried in a vacuum centrifuge and reconstituted in water with 2% acetonitrile (ACN).

All mass spectrometry runs were performed on an Orbitrap Fusion^TM^ Lumos^TM^ Tribrid^TM^ instrument coupled to a Dionex Ultimate3000 RSLCnano system (Thermo Fisher Scientific). Specifically, peptides were resolved on a Thermo Scientific Dionex UltiMate 3000 RSLC system using a PepSep 150umx25cm C18 analytical column (PepSep, Denmark) with 1.5μm particle size (10 Å pores), heated to 49 °C. Separation was performed in a total run time of 90 m with a flow rate of 0.520 μL/m with mobile phases A: water/0.1% formic acid and B: 80%ACN/0.1% formic acid. Peptides eluting from the LC were electrosprayed directly into a tribrid Lumos mass spectrometer (Thermo Fisher Scientific), operated in data-independent acquisition mode, DIA. A survey full scan MS (from *m/z* 350 to 1200) was acquired in the Orbitrap at a resolution of 120,000 (at 200 *m/z*). The automatic gain control (AGC) target for MS1 was set to 250% (1e6), and the injection time was set to Auto. For the fragmentation spectra, the following settings were used: the mass range of 350–1200 Da was segmented into 19 windows overlapping by 1 Da, with an isolation window width of 45.7 Da. They were fragmented via higher collisionally induced dissociation (HCD) at 33% normalized collision energy. AGC target set to 1000% (5e5), and ion filling time was set to Auto. Detection was in the Orbitrap, at a resolution of 15,000.

LCMS files were processed with Spectronaut v.20 (Biognosys, Zurich, Switzerland) using DirectDIA analysis mode. Mass tolerance/accuracy for precursor and fragment identification was set to default settings. The reviewed FASTA for yeast and a FASTA of 381 common laboratory contaminants (https://github.com/HaoGroup-ProtContLib) were used. A maximum of two missing cleavages was allowed, the required minimum peptide sequence length was 7 amino acids, and the peptide mass was limited to a maximum of 4600 Da. Carbamidomethylation of cysteine residues was set as a fixed modification, and S,T,Y phosphorylation, M oxidation, and protein N-termini acetylation were set as variable modifications. A decoy false discovery rate (FDR) at less than 1% for peptide spectrum matches and protein group identifications was used for spectra filtering (Spectronaut default). Decoy database hits, proteins identified as potential contaminants, and proteins identified exclusively by one site modification were excluded from further analysis.

The signal in each sample for Cdc28 (in Dataset EV1/Sheet1_IdentifiedProts) or pT169-Cdc28 (in Dataset EV1/Sheet2_PhosphoQuant) was first normalized against the total “input” signal in each case. The ratio of the two normalized values (i.e., pT169-Cdc28 : Cdc28) was used to compare the different samples (Fig. 1B). All the data from this mass spectrometry experiment are in Dataset EV1.

### Protein surveillance

#### Immunoblotting

Protein extracts were made as described (Wallace et al, 2022), and resolved on 12% Tris-Glycine SDS–PAGE gels, unless indicated otherwise. V5-Cak1 was detected using a mouse anti-V5 tag monoclonal antibody, conjugated with horseradish peroxidase (HRP), used at a 1:5000 dilution (ThermoFisher; Cat#: R961-25). Loading was measured with a mouse anti-Pgk1 primary antibody, followed by a secondary antibody (at 1:5000; ThermoFisher; Cat#: 31431). To detect total amounts of Cdc28, we used a commercially available mouse monoclonal anti-Cdc28 antibody (at 1:1000; Santa Cruz Biotechnologies #sc-515762).

For detecting the activating phosphorylation of Cdc28, we obtained a custom rabbit polyclonal antibody (Biomatic, Kitchener, Ontario, Canada) raised against a peptide (RAFGVPLRAY(pT)HEIVT) encompassing T169 in the yeast Cdc28 protein. The antibody (α-pT169) recognized a protein of the same apparent mass as one recognized by the anti-Cdc28 antibody (Fig. 1C, compare the middle and right panels on the top). The signal was diminished (Fig. 1C, middle panel on the top) when the samples were first treated for 30 m at 30 °C with 400 u of Lambda Protein Phosphatase (New England Biolabs #P0753). The reduction in signal intensity was comparable to the reduction observed for ribosomal protein S6 phosphorylation (Fig. 1C, compare the left and middle panels on the top). The latter was detected by a specific rabbit monoclonal antibody against Ser235/236 of the human protein (at 1:5000; Cell Signaling, Cat#: 4858), followed by a secondary antibody (at 1:5000; ThermoFisher; Cat#: 31466).

#### V5-Cak1 stability assay

To monitor the stability of V5-Cak1 (Fig. EV1C), we transformed BY4742 haploid cells with the pCUP1-V5-CAK1 plasmid described above. The transformants were cultured in synthetic complete drop-out medium (SC-Ura), with glucose or glycerol as the carbon source. Cultures proliferating exponentially were induced with CuSO_4_ for 20 m, and then treated with cycloheximide (CHX; 100 μg/mL) to block protein synthesis. The levels of V5-Cak1 were then monitored by immunoblotting as described above, from samples taken at various time points.

In addition to the above inducible system, we also used strains expressing at endogenous levels V5-Cak1 (strain MSP258; uORF-35, V5-CAK1) or Cln3-13Myc (strain HB490), to query the stability of these proteins after addition of CHX (100 μg/mL). Cln3 is known to be unstable and serves as a control (Fig. EV1D–F). The band intensities at each time point were quantified with ImageJ and normalized against the intensities of Pgk1, serving as a loading control, and then plotted as shown in Fig. EV1F to obtain the estimated t_1/2_ value.

#### Image analysis

Images were processed with the “Subtract Background” tool, and band intensities quantified using the “Measure” tool to obtain a mean intensity for each band, using the ImageJ software package. The area measured was kept constant for a particular sample series for each blot analyzed. The source data for all the immunoblots are in the supporting files.

### Transcript abundance using digital droplet PCR (ddPCR)

For RNA surveillance, RNA extracts were made as described previously (Blank et al, 2024, 2020). Briefly, the ddPCR reaction mixture was prepared by following the manufacturer’s protocol (One-Step RT-ddPCR Advanced Kit for Probes), using the TaqMan® hydrolysis probes labeled with FAM-MBP for *CAK1* and VIC-MBP for *UBC6* reporter fluorophores. The abundance of the transcripts was obtained using the QuantaSoft™ Software. Transcript levels of *CAK1* were normalized against the corresponding transcript levels of *UBC6* in each sample.

### Centrifugal elutriation and cell size measurements

In each experiment, a 250 mL cell culture was grown to a density typical of mid-log cultures (~1-5E + 07 cells/mL in YPD), then loaded onto a 40 mL elutriator chamber at a flow rate of 35 mL/min and centrifuged at 3200 rpm. The cells were washed twice with 250 mL of medium, first at 2800 rpm, then at 2400 rpm. Finally, cells were elutriated with 250 mL of the same medium at a pump speed of 38 mL/min and centrifuge speed of 2400 rpm. This early G1 daughter cell fraction was isolated and monitored regularly as it progressed through the cell cycle.

From live, unfixed cells, the percentage of budded cells was determined via phase microscopy, and cell size was measured using a Beckman Z2 channelyzer (Hoose et al, 2012; Soma et al, 2014). The Beckman Accucomp software was used to generate size histograms, from which the geometric mean size was calculated. In addition, the ‘birth’ size of asynchronous cultures was determined from the size histograms using established methods (Maitra et al, 2019; Truong et al, 2013). Briefly, the population to the left of the peak of the cell size histogram was assumed to contain daughter cells, and the size corresponding to the 10% smallest of these cells was taken as ‘birth size’.

### Doubling time measurements in batch cultures

For experiments shown in Fig. EV2, cell numbers were determined by direct counting using a hemocytometer, from shake flask cultures at 30 °C sampled at regular intervals over a day. From the same measurements, the budding index and cell size were also measured. Natural log-transformed cell numbers were plotted against time (h). The specific growth rate (*k*, in h^−1^) was derived from the slope of the linear regression during the exponential phase. Doubling time (Td) was calculated using the equation: Td=ln2/*k*.

For experiments in Figs. 5C and EV4, optical density at 600 nm was measured as a proxy for cell density using an Agilent Synergy H1 plate reader. Cultures (0.2 mL per well) were incubated at 30 °C with orbital agitation for 10 s prior to hourly readings. TD estimates were calculated as described above, using the linear portion of the growth curves between the 2 h and 10 h timepoints.

### Viability assays (colony-forming units)

For the experiments shown in Fig. 5A, to assess reproductive viability, yeast cultures (in YPD) were left standing at 30 °C. Six independent cultures for each of the two strains (SCMSP256, *uORF-16*; and SCMSP257, *uORF-0*) were evaluated. At the indicated time points over 20 days, aliquots from each culture were collected and serially diluted (1:20,000 final dilution) in sterile distilled water. Aliquots of 50 µL were then spread onto YPD agar plates, using three plates for each culture. Plates were incubated at 30 °C for 48–72 h until colonies reached a countable size. Viability for each culture was calculated as a percentage of colony-forming units relative to the first time point. Since we queried the same cultures over multiple time points, longitudinal measurements were evaluated with mixed-effects models. We used the lme4 and lmerTest R language packages with the following syntax: lmer(viability ~ time + strain + (1 | ID), data = Fig. 5A). It fits a model where “viability” is the response variable, and “time”, and “strain” are the fixed effects. “ID” is an individual replicate culture of the same strain, considered as a random effect to account for the non-independence of measurements taken from the same cultures over time.

### Flow cytometry and fluorescence microscopy

The percentage of cells in the G1 phase of the cell cycle (%G1) was quantified as detailed in previously published protocols (Hoose et al, 2012, 2013). For the experiments in Fig. 6A, we used a Cytek® Muse® Cell Analyzer. Because of *S. cerevisiae*’s small genome size and the close spacing of DNA peaks in the histograms, which often makes the application of software developed for the analysis of DNA content in mammalian cells problematic, we used the following approach: Briefly, the area under the left half of the G1 peak in the DNA content histogram was measured, doubled to account for the entire G1 peak, and then divided by the total area under the histogram. This fraction, representing the proportion of cells with G1 DNA content (1 N for haploid cells), was multiplied by 100 to express it as a percentage (%G1). For the experiments in Fig. EV3B, we used a BD Accuri C6 Flow Cytometer at the Flow Cytometry Facility of the Texas A&M University College of Veterinary Medicine & Biomedical Sciences. The calculated %G1 fraction in this case was from FlowJo^TM^ flow cytometry software (BD Biosciences, Franklin Lakes, NJ).

For the experiments in Fig. 3, BY4743 cells carrying the mTurquoise2 and mRuby2 reporter plasmids were grown to mid-log phase at 30 °C in synthetic complete (SC) medium lacking leucine and uracil, supplemented with either glucose or glycerol as the carbon source. To prepare samples, exponentially proliferating cultures were briefly sonicated to separate cell clumps. Cells were then collected by centrifugation at 5000× *g* for 1 m, and the pellets were resuspended in phosphate-buffered saline (PBS) containing 0.5% ^w^/_v_ bovine serum albumin (BSA). Samples were protected from light and processed immediately for analysis, using a BD Accuri C6 Flow Cytometer at the Flow Cytometry Facility of the Texas A&M University College of Veterinary Medicine & Biomedical Sciences.

For fluorescence microscopy, the samples were prepared as above and promptly imaged using a Nikon TS100 inverted microscope equipped with a Plan Apo 100X oil DIC objective and a CoolSNAP DYNO camera. The filters used were an AT-DAPI filter set (excitation at λ_*ex*_ ≅ 360–390 nm) for imaging the mTurquoise2 reporter, and a DM575-TRITC filter set (excitation at λ_*ex*_ ≅ 540–580 nm) for imaging the mRuby2 reporter.

### Sample size and replicates

The number of samples in each experiment was not determined based on a formal power analysis. There was no blinding in the experimental design. All replicates represented distinct biological samples from separate cultures. Every datapoint shown is from a separate experiment. In experiments with a minimum of three replicates, statistical comparisons between groups were performed using a robust bootstrap ANOVA (t1waybt function, with the number of bootstrap samples set to 599) followed by post hoc tests (mcppb20 function) from the WRS2 R package (Mair and Wilcox, 2020). Bootstrap ANOVA was chosen to account for unequal group variances and small sample sizes, which violate the assumptions of a standard one-way ANOVA. For experiments with at least four replicates, nonparametric statistical methods were also employed, as indicated in each case. All collected data points, including any potential outliers, were included in the analyses.

### Graphics

Figure 1A was created with BioRender.com.

## Supplementary information

Peer Review File

Source data Fig. 1

Source data Fig. 2

Source data Fig. 3

Source data Fig. 4

Source data Fig. 5

Source data Fig. 6

Figure EV1 Source Data

Figure EV2 Source Data

Figure EV3 Source Data

Figure EV4 Source Data

Figure EV5 Source Data

Expanded View Figures

## Data Availability

This study includes no data deposited in external repositories. The source data of this paper are collected in the following database record: biostudies:S-SCDT-10_1038-S44319-026-00916-z.
