# Supplementary material for: uORF-mediated translational control of CAK and Cdk activation in response to low nutrients in yeast
Source: EMBO Rep. 2026 Sep 5;27(19):5582–605. doi: 10.1038/s44319-026-00916-z (PMC13649776; doi:10.1038/s44319-026-00916-z)
Supplement: Supplementary file 1 [file 44319_2026_916_MOESM13_ESM.pdf]

Expanded View Figures

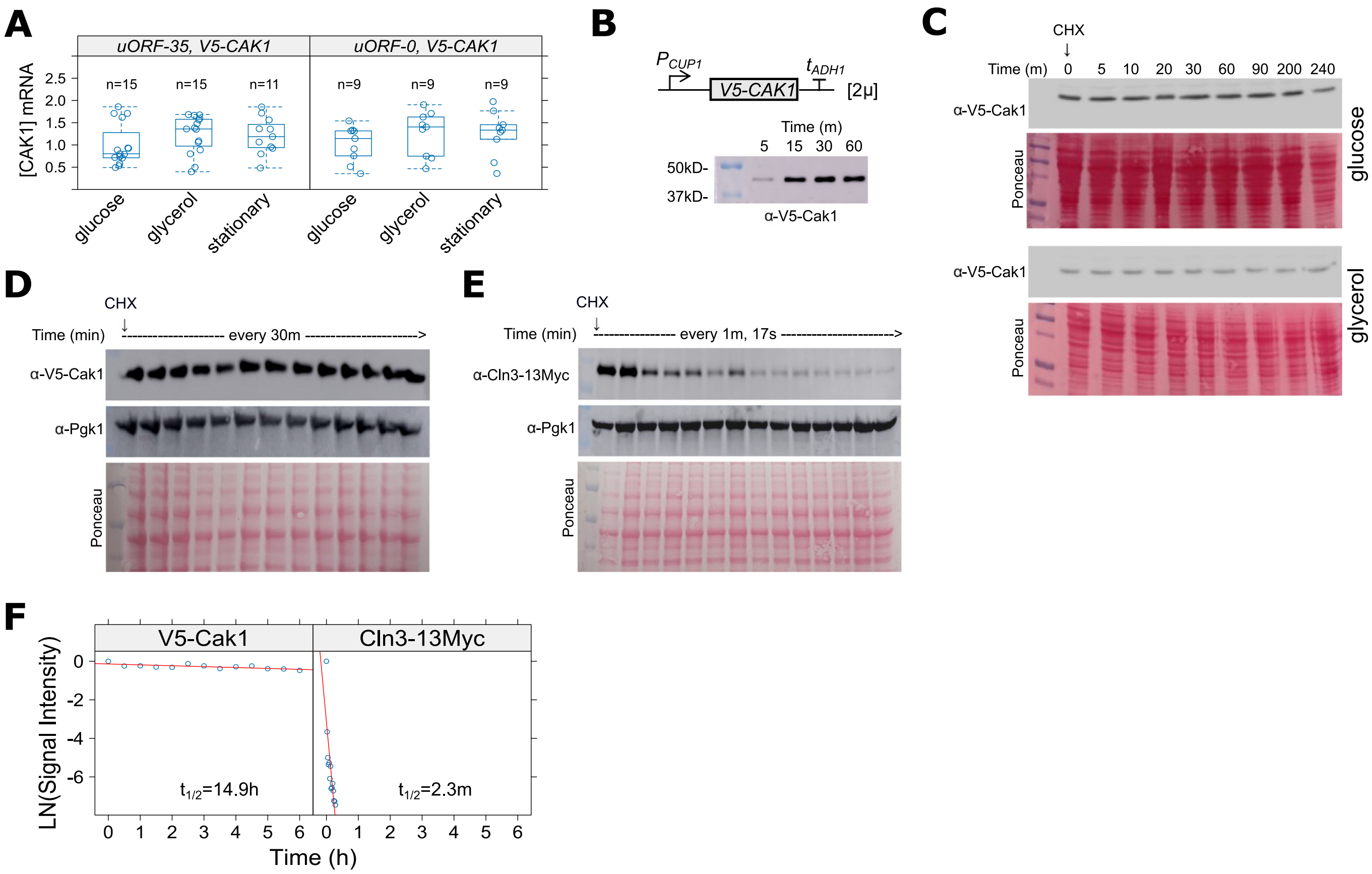

Figure EV1. *CAK1* expression is not regulated by transcription or proteolysis as a function of growth.

(**A**) Relative steady-state levels of *CAK1* mRNA from multiple independent experiments (*y* axis) across the indicated growth conditions (*x* axis). Strains and growth conditions are identical to those in Fig. 1C. Boxplots illustrate the distribution of normalized levels across biological replicates; the horizontal line represents the median, box boundaries indicate the interquartile range (IQR), and whiskers extend to the minimum/maximum values within 1.5 times the IQR. Individual open circles represent independent experimental data points. mRNA levels were measured by ddPCR and normalized to the mean of exponentially growing *uORF-35 V5-CAK1* cells (first box). No significant differences were found between strains or conditions ($P > 0.05$, Kruskal–Wallis test). Every datapoint is from a separate experiment. (**B**) (Top) Schematic of the plasmid used for copper-inducible synthesis of V5-Cak1. (Bottom) Immunoblot of cells (BY4742 background) carrying the plasmid, showing V5-Cak1 protein levels (detected via an anti-V5 antibody) at the indicated times after induction with $CuSO_4$. (**C**) V5-Cak1 protein stability in glucose and glycerol. Exponentially proliferating cells (BY4742 background) carrying the pCUP1-V5-CAK1 plasmid (described in **B**) were induced for 20 m with 0.5 mM $CuSO_4$ and then treated with cycloheximide (CHX; 100 μg/mL) to block translation. V5-Cak1 levels were monitored by immunoblotting (top panels) in glucose (upper set) and glycerol (lower set) media. Total protein loading is shown via Ponceau S staining (bottom panels). (**D**) A similar cycloheximide-chase experiment as in (**C**) was performed in YPD using strain MSP258, expressing V5-Cak1 from its endogenous locus. Immunoblots show levels of V5-Cak1 (top) and Pgk1 (middle) as a loading control. Total protein is shown by Ponceau S staining (bottom). (**E**) A similar experiment to (**D**) was performed using strain HB490, which expresses Cln3-13Myc from its endogenous locus. (**F**) The natural logarithm (LN) of signal intensities (*y* axis), normalized to Pgk1, is plotted against time (*x* axis). Red lines represent linear regression fits. V5-Cak1 (left) remains stable over the 6-h course, whereas Cln3-13Myc (right) is rapidly degraded. Estimated half-lives, $t_{1/2}$, were derived from the slopes of the regression fits. Source data are available online for this figure.

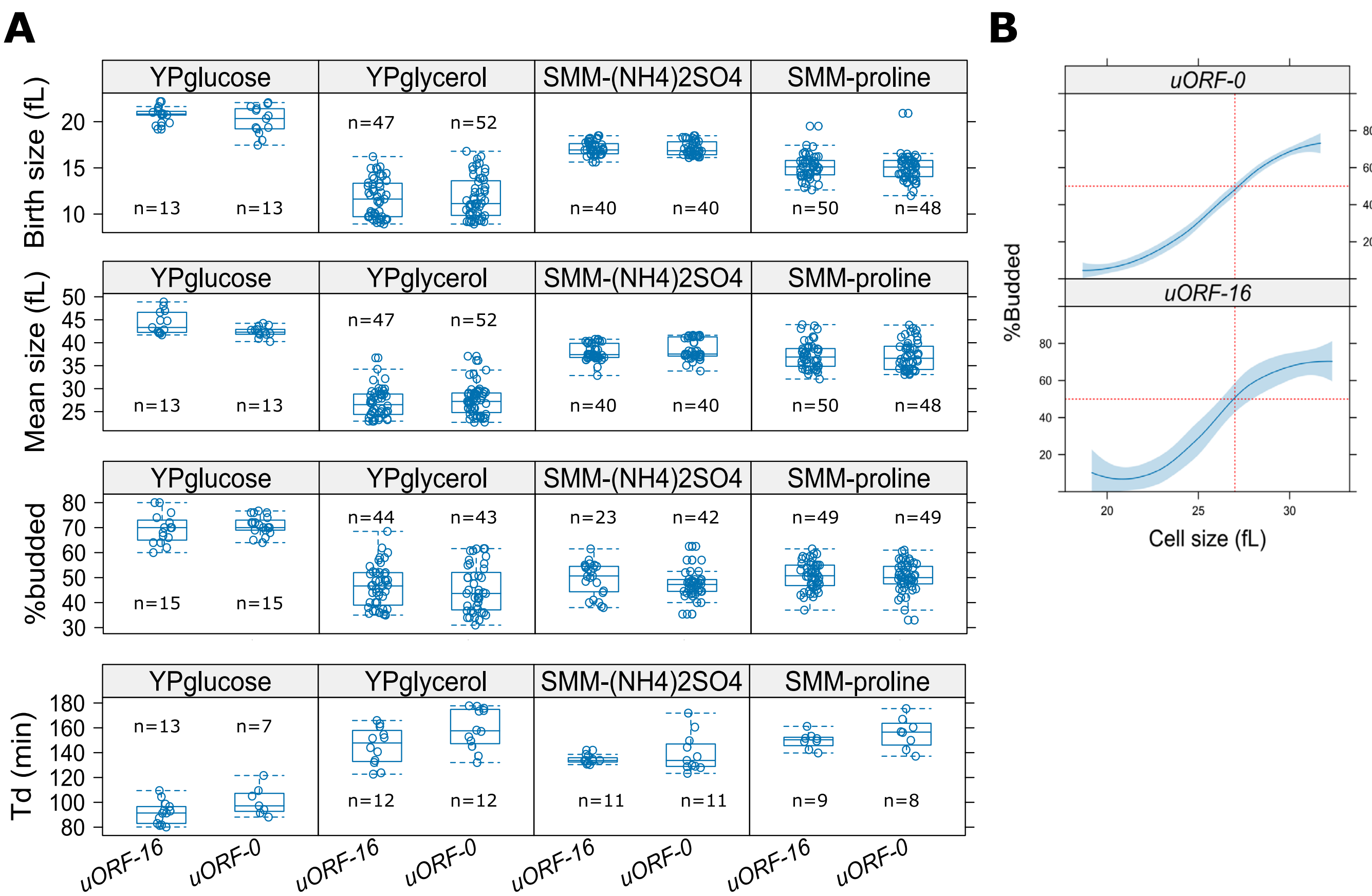

Figure EV2. Eliminating the uORF of *CAK1* does not affect cell cycle kinetics in batch cultures.

(**A**) Cell cycle parameters for the *uORF-16 CAK1* (MSP256) and *uORF-0 CAK1* (MSP257) strains are shown on the *x* axis, grown in the indicated media. The boxplots, drawn as described in Fig. EV1, show (from top to bottom): birth size, mean cell size, %budding, and doubling time (Td), on the *y* axis. Values were obtained from independent experiments. No significant differences were found between the strains ($P > 0.05$, Wilcoxon rank-sum test). Every datapoint is from a separate experiment. (**B**) Cell cycle progression of elutriated synchronous early G1 cells in YP-glycerol. The percentage of budded cells is plotted against cell size (fL) for the same strains as in (**A**). The plots show Loess curves with 95% confidence intervals. Red lines indicate the critical size for budding (the size at which 50% of cells have budded).Source data are available online for this figure.

 © The Author(s)

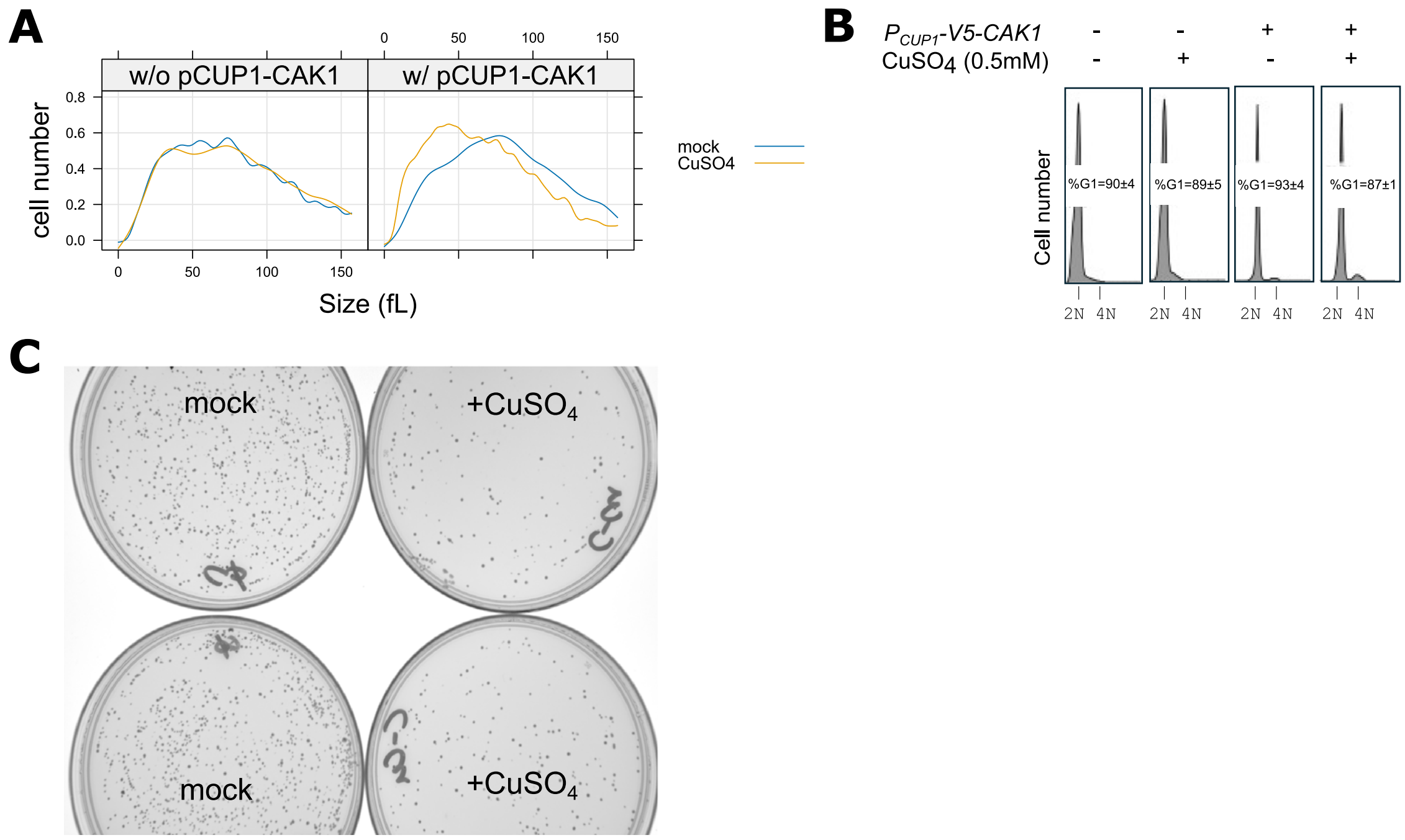

Figure EV3. Overexpression of *CAK1* in the stationary phase reduces cell size and viability.

(**A**) Cell size distribution of stationary phase cells (BY4743) with (right panel) or without (left panel) the pCUP1-V5-CAK1 plasmid. Histograms show the fraction of cell counts (*y* axis) by size (*x* axis) from 6 d cultures. Density lines represent cultures induced with 0.5 mM $CuSO_4$ to express V5-Cak1 (yellow) versus mock-treated controls (blue). (**B**) DNA content analysis of the same samples shown in (**A**), measured by flow cytometry. Histograms show cell counts (*y* axis) vs. fluorescence per cell (*x* axis). The average percentage (± SD) of cells in the G1 phase from three independent cultures is indicated. (**C**) Representative cell viability plates for cultures carrying the pCUP1-V5-CAK1 plasmid, as shown in (**A**, right panel). Cultures were either mock-treated (left plates) or induced with $CuSO_4$ (right plates) before plating on media lacking $CuSO_4$. Viability dropped from 67% in mock-treated cultures to 19% in $CuSO_4$-treated cultures ($\chi^2 = 8123.4$, df=1, $P < 2.2e\text{-}16$).Source data are available online for this figure.

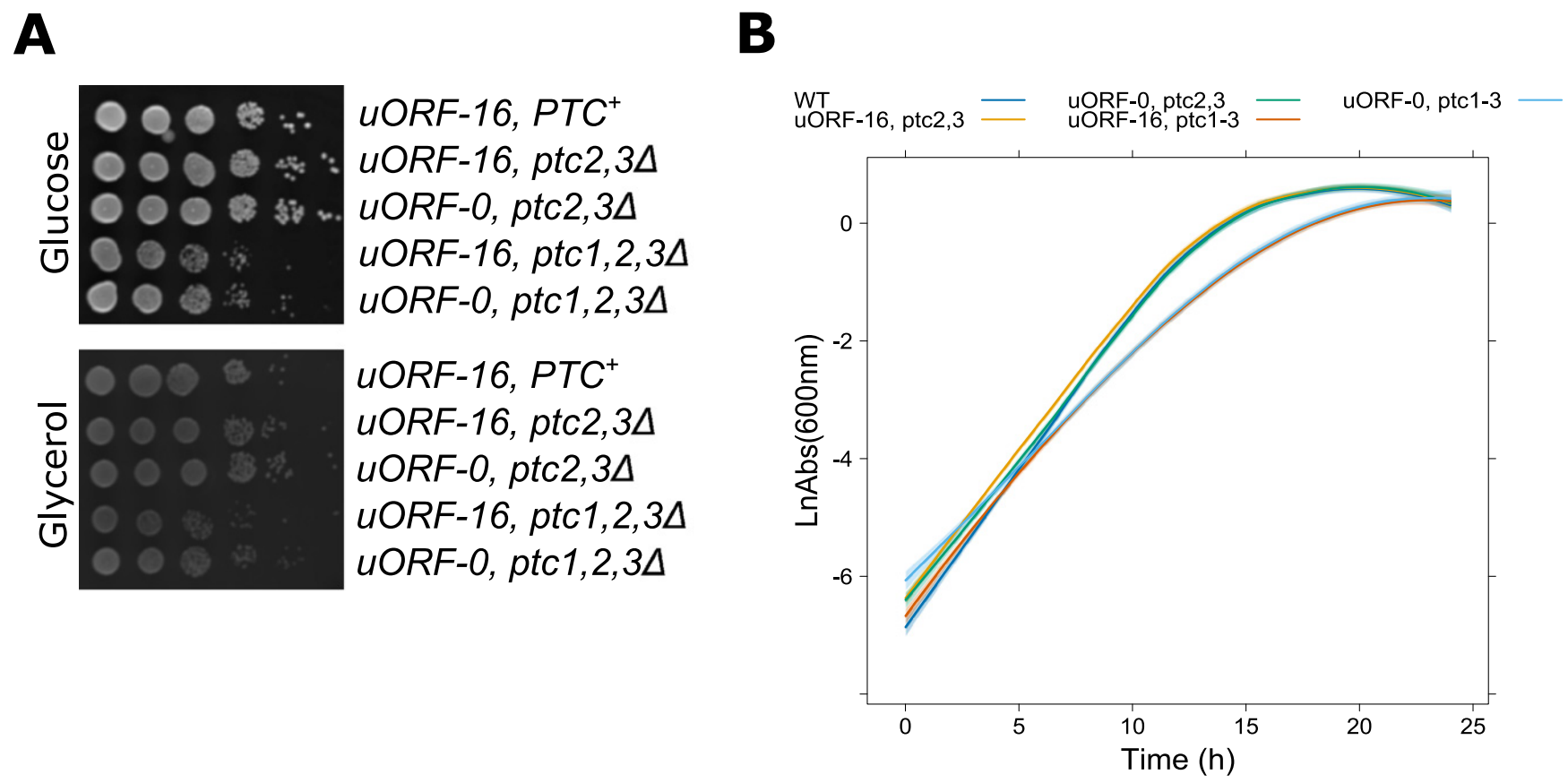

Figure EV4. Proliferation of type 2C protein phosphatase mutants with or without the uORF in *CAK1*.

(**A**) Growth spot assays to assess the relative growth fitness of the indicated strains (all in the BY4742 background) on glucose (YPD) or glycerol (YPGlycerol) media. Overnight cultures were normalized to an initial cell density, and aliquots were spotted at tenfold serial dilutions. The plates were imaged after 2 (glucose) or 3 (glycerol) days at 30 °C. (**B**) Growth curves of the strains shown in (**A**), cultured in YPD glucose medium. Ln-transformed absorbance values (600 nm) are plotted on the *y* axis against time (*x* axis). Measurements were taken using a microplate reader and corrected for background absorbance of the sterile medium. Data are presented as Loess regression curves; shaded regions represent 95% confidence intervals ($n = 8$ independent replicates per strain).Source data are available online for this figure.

 © The Author(s)

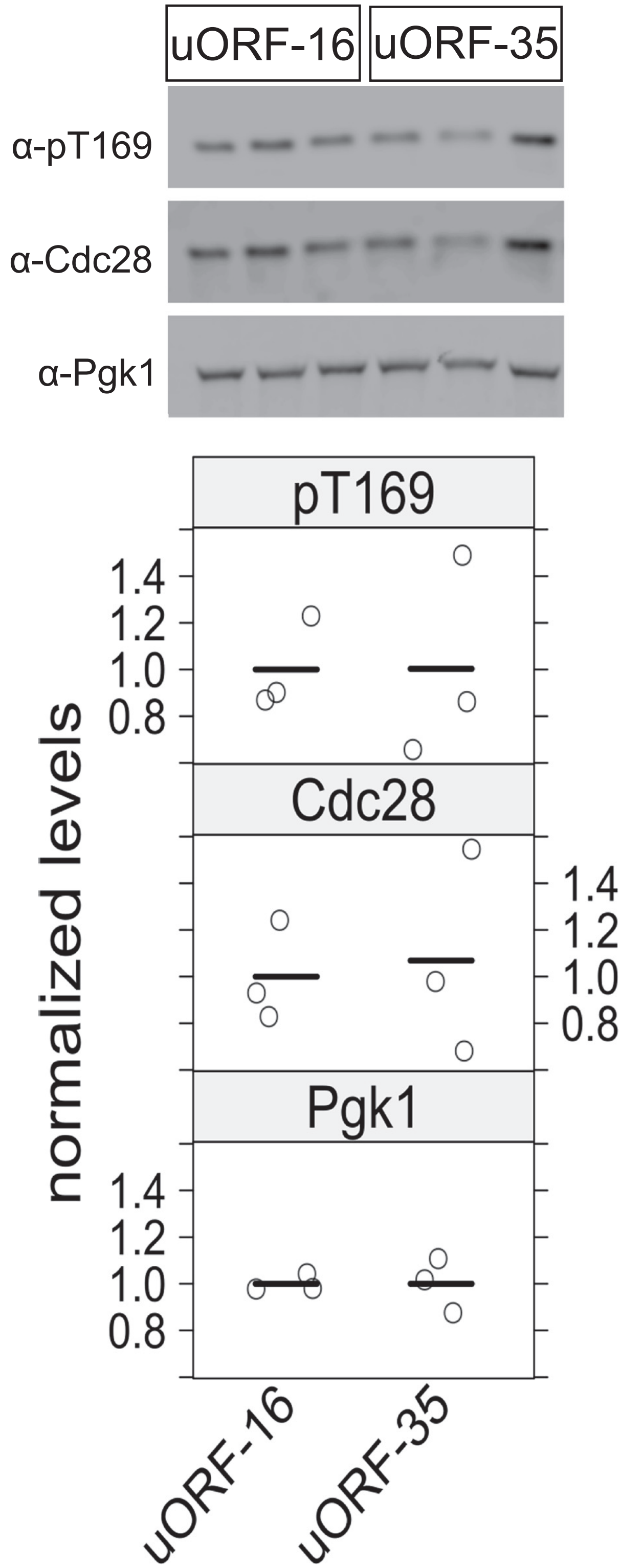
uORF-16
uORF-35
α-pT169
α-Cdc28
α-Pgk1
pT169
Cdc28
Pgk1
normalized levels
1.4
1.2
1.0
0.8
uORF-16
uORF-35

◀ **Figure EV5. The levels of Cdk T169 phosphorylation are the same in uORF-16 and uORF-35 cells.**

Immunoblots of pT169-Cdc28, Cdc28, and Pgk1 from the indicated strains (both in the X2180-5A background) from three independent cultures grown in glucose are shown at the top. The strip plot graphs below display the quantification of each target from the three independent experiments shown on each gel. Band intensities were quantified, and their normalized values were calculated as in Fig. 6C for each target protein and plotted on the *y* axis; the *x* axis indicates the respective strains.Source data are available online for this figure.

 © The Author(s)
