# Supplementary material for: uORF-mediated translational control of CAK and Cdk activation in response to low nutrients in yeast
Source: EMBO Rep. 2026 Sep 5;27(19):5582–605. doi: 10.1038/s44319-026-00916-z (PMC13649776; doi:10.1038/s44319-026-00916-z)
Supplement: Supplementary file 2 [file 44319_2026_916_MOESM1_ESM.pdf]

uORF-mediated translational control of CAK and Cdk activation in response to low nutrients in yeast

Heidi Blank, Eun-Gyu No, Ainsley Nelson, Abigail Payne, Sofia Lykidis, and Michael Polymenis

Corresponding author(s): Michael Polymenis (polymenis@tamu.edu)

Review Timeline:

Transfer Date:	18th Jun 26
Editorial Decision:	24th Jul 26
Revision Received:	11th Aug 26
Accepted:	20th Aug 26

Editor: Esther Schnapp

Transaction Report: This manuscript was transferred to EMBO Reports following peer review at Review Commons.

(Note: With the exception of the correction of typographical or spelling errors that could be a source of ambiguity, letters and reports are not edited. Depending on transfer agreements, referee reports obtained elsewhere may or may not be included in this compilation. Referee reports are anonymous unless the Referee chooses to sign their reports.)

Review #1

1. Evidence, reproducibility and clarity:

Evidence, reproducibility and clarity (Required)

The study "Translational control of CAK and Cdk T-loop phosphorylation in response to growth in yeast" by Blank et al investigates the regulation of the CDK activating Kinase (CAK) in yeast. This conserved kinase phosphorylates a conserved residue on the main cell cycle kinase Cdk1, which is threonine T169 in yeast. While it has long been known that the phosphorylation is required for full Cdk activity, little is known (particularly in yeast) how this site is regulated and what the physiological relevance of this regulation would be. This is particularly interesting since this phosphorylation does not seem to be regulated during the cell cycle per se.
Here, the authors show that the phosphorylation of T169 is nutrient/growth rate dependent. Both the fraction of phosphorylated T169 and the abundance of CAK itself are regulated with nutrients/growth rate. The authors show that CAK is mainly regulated postranscriptionally by a uORF directly upstream of the CAK ATG. This suggests that the levels of CAK are regulated by ribosome availability (though this is only inferred and not directly demonstrated), i.e. biosynthetic capacity of the cell. Surprisingly though, the abundance of CAK does not correlate well with the amount of T169 phosphorylation, implying additional layers of regulation. The authors look for this at the level of dephosphorylation by Ptcs and present some evidence that this is the case. More convincing I find the evidence that dysregulated CAK prevents ordered entry into stationary phase/quiescence.

Overall, I find the results very intriguing and in sum mostly convincing. However, I have some major issues with data representation and also transparency as to how data was generated and what individual data points mean. Although my list of major points is long, most of them can be addressed by replotting existing data or clarifying things in the text. Additionally, I have some optional suggestions on how to connect some of the dots and make the story more coherent, depending on the timeline and journal the authors are aiming for, see significance statement.

Major points:

1. Box plots are meaningful only for larger datasets, I would suggest at least 20 datapoints. Box plots with less than five datapoints are by definition mathematical nonsense! Please replace all boxplots with few data points by something reasonably, such as showing the

individual points as dots and the average as a line etc.
2. Comparison of uORF 16 and 35 is missing. It is of course very unfortunate that the protein required N-terminal tagging, sometimes you just have to deal with what is possible. But, I dont see anywhere a thorough comparison of the uORF 16 and 35. The authors state in the methods that they dont see any difference in the growth rates and budding. But what about fraction of T169 phosphorylation? What about viability in stationary phase etc.? The authors try to correlate the biochemical data with the phenotype, but do this with two different strains (that even have slightly different backgrounds if I interpret the methods correctly).
3. Figure 1: B-> exchange box plot. C-> I dont see how the pRps6 AB validates anything. Assuming you have a working phosphatase, then your AB has a residual affinity towards the unphosphorylated site. Just admit that you have some % background and fine. D. Again, replace box plots.
4. Figure 2: A-> the overview figure is in principle great, but I found the illustration confusing. 2B-> Again boxplots are non-sensical here. Maybe plotting the three strains in one plot rather than (or in addition to) comparing the strain to itself under different conditions would be helpful, especially to compare with EV2A.
5. Figure EV2: It is not stated here or in the methods how these experiments were performed. Are these growth experiments in shake flasks? Or in multiwell-plates? If multi-well plates, were the wild-type and mutant paired, or was everything done on a single day? How many replicates were performed? When was the size/budding data taken? Once per experiment or is it an average across exponential phase?
6. EV2: Why is the data so extremely noisy? There seems to be up to 50% difference in growth rate even in the wildtype. This makes no sense. Maybe a simple OD-based growth assay would be better than manual cell counting (why was this done?) There seems to be a clear overall trend that the uORF-0 grows slightly slower, but this is masked by the noise in the data.
7. Figure 3. This is in principle a very powerful assay to prove the point. It might have been better to put both reporters on one plasmid for ratiometric analysis. A. Labelling reporter 2 CAK1-mRuby in the figure gives the impression this is a fusion protein, but I am assuming it is just mRuby, or mRuby with just a small N-terminal section of CAK. How long is the actual uORF in the mRuby construct? B. The localization of the Turquoise looks weird, any explanation? C. It is a bit unclear how many replicates where done and what the induvial data points refer to. Are these averages of x number of cells in y-number of flow cytometry experiments? The y-axis makes no sense. What ratio are you referring to? Wouldnt it make sense to show the mRuby to mTurquoise ratio per cell instead of normalizing within the fluorophore? Why is there no difference between glucose and glycerol in reporter 2? Isnt that one of the key points about the idea how the uORF works?

8. Figure EV3: B - Are these diploid cells? Why?
9. Figure 4: The authors completely brush over the fact that in the uORF 0, the V5-Cak actually decreases with growth rate. In fact, it decreases considerably more, than the Cak increases in the uORF35. What could this mean for regulation?
10. Figure 4C: I understand that running these chemostats is a lot of work so you cannot run all of them in replicates. However, it might be worth looking at the critical dilution where the two strains differ and repeating those to see if the differences in the curves are noise or if the strains actually have a different "critical point".
11. Figure 5: A- The difference in viability is by far the most convincing phenotype shown. Yet, it seems to come from only one replicate? This is a reasonably simple experiment to do, I would recommend triplicates and then putting more emphasis on this finding, see below. B. Here, it seems there was also only one replicate each? Maybe replicate just the carbon limited experiment? At higher dilution rate, the mutant seems to have an advantage. Do the authors have any thoughts on this?
12. Figure 6: The authors should show the T169 phosphorylation in these mutants. Does this somehow correlate with the G1 fraction? There does seem to be something going on with Ptc, but I find the data only semi-convincing. The triple mutant per se likely has all kinds of defects. Working with Phosphatases is tricky. But maybe trying to find a phenotype in steady-state growth is not what the authors should be aiming for, see significance.
13. Figure 7. Both the graphical abstract and Figure 7 are overstating the findings. "Schematic of our findings" is certainly not true. Maybe better "putative model of G1/S control through translation". Formally, the authors did nothing to show that it has to do with ribosome availability. Also, I am not sure if it has been shown that the "freak" cyclin Cln3 actually needs pT169 phosphorylation. What also doesn't become clear in the picture or anywhere else is that CAK and Cln3 operate on different timescales due to their very different protein stability.
14. The discussion is extremely superficial and does not do a good job of connecting the findings to the literature (the authors cite a total of four papers, three of which are their own).
15. In general, the reference list is very self-centred. To a certain extend this is normal, but I find this one extreme. There is not a single paper cited that shows the connection of ribosome content/growth rate/growth condition. Also, there has been so much recent work on connecting nutrient signalling/growth control to cell cycle progression. It would have been so much nicer to see a discussion of these results in a bigger context.
16. Methods: Some of the methods are very detailed, some of them completely lack critical information. For example, how were cells grown (shakeflasks, mutliwell-plates…?)? Also, "We gave it to the facility" is NOT a method. I dont know if this was laziness or sloppiness, but I am sure the facility can provide a proper protocol.

17. Generally, the proteomics data is not well accessible. As far as I can tell, the raw data was not deposited on PRIDE etc. And the "source data" provided for Fig 1B is a non-annotated csv that does not contain the detected peptides or any other information typically provided in proteomics experiments. Or did I miss an additional table somewhere?

Minor Points:

1. EV figures dont map 1:1 to main figures. This of course depends on the journal, but I find it confusing.
2. Figure 1D and 2B-> y-axis label is confusing. Call it normalized abundance or something like that. What is an "expressed ratio"? Apart from that, you later show that it is not expression but translation.
3. EV3: "Histograms show cell counts", no it doesn't, it shows fractions. There is generally a lot of imprecision in the figure legends.
4. In many figures the position of the axis labelling is weird.

2. Significance:

Significance (Required)

I think the observations from this study are highly significant to the yeast cell cycle field, and also relevant for the entire cell cycle/ growth control field. I am sure the study is publishable within its current scope in a decent journal if the technical issues are fixed. However, in its current state there are so many loose ends that would be reasonably easy to connect. It is a pity that the authors dont really try to develop a model of what is going on and why this regulation is important. Clearly, in steady state the cell does not seem to particularly care about the precise CAK levels and the T169 levels are mainly controlled by phosphatase activity. So why would a protein with a half-life of 4 hours be translationally regulated? It must operate on a completely different logic than Cln3, which has a half-life of only a few minutes. The most obvious phenotype that the authors observe is a failure to enter stationary phase properly. So why not investigate this a bit more closely? Just run growth curves in shake flask and monitor growth, CAK levels, T169 phosphorylation etc over the transition from glucose to ethanol to stationary phase.
Also, one main advantage of a translation-based regulation rather than a transcriptional regulation would be a much faster re-activation upon exit from stationary phase. Maybe that is something to look at?

3. How much time do you estimate the authors will need to complete the suggested revisions:

Estimated time to Complete Revisions (Required)

(Decision Recommendation)

Between 1 and 3 months

4. *Review Commons* values the work of reviewers and encourages them to get credit for their work. Select 'Yes' below to register your reviewing activity at [Web of Science Reviewer Recognition Service] (formerly Publons); note that the content of your review will not be visible on Web of Science.

Web of Science Reviewer Recognition

No

Review #2

1. Evidence, reproducibility and clarity:

Evidence, reproducibility and clarity (Required)

The authors have discovered an interesting regulatory mechanism governing the cellular levels of Cak1, the essential Cdk-activating kinase of budding yeast. Cak1 phosphorylates T169 in the major Cdk, Cdc28, resulting in full activation of Cdc28 and cell-cycle progression. Surprisingly, and despite its central importance, Cak1 is not believed to be regulated during the cell cycle - apart from previous evidence (Kaldis et al 1998) that Cak1 levels decline when proliferation is inhibited by starvation (stationary phase). The current paper revisits the question of Cak1 regulation and uncovers evidence that growth conditions govern Cak1 levels by changing its rate of translation. An upstream ORF appears to reduce Cak1 translation in slow growth conditions. The experiments are generally convincing and clear, and a variety of rigorous approaches have been used.

The paper begins in Figure 1 with clear evidence that T169 phosphorylation on Cdc28 declines in poor growth conditions and is essentially absent in stationary phase. This decline in pT169 is accompanied by a decline in Cak1 levels. The authors then uncover the mechanism that reduces Cak1 levels. After ruling out some mechanisms (no changes in Cak1 mRNA abundance or Cak1 stability), they pursue the role of a small uORF that starts just upstream of the Cak1 start codon. The results in Figure 2 demonstrate the mutation of

the uORF start codon increases Cak1 levels significantly in poor growth conditions, and even a very short uORF reduces Cak1 levels, suggesting that translation initiation at the upstream site reduces Cak1 translation in poor growth conditions. Experiments with fluorescent reporter genes in Figure 3 further demonstrate the inhibitory effect of the uORF on translation. In Figure 4, chemostat experiments under various growth conditions further support these conclusions. All these experiments provide a convincing case that the uORF is responsible for reduced Cak1 levels in poor growth conditions.

The story becomes less straightforward when the authors analyze T169 phosphorylation in these experiments. There is a significant lack of correlation between Cak1 levels and T169 phosphorylation. In Figure 2, mutation of the uORF restores Cak1 levels in stationary phase but does not affect T169 phosphorylation, suggesting that Cak1 is not the sole determinant of pT169 levels, and perhaps that some other mechanism is also suppressing phosphorylation (Activation of a phosphatase? More rapid Cdc28 dephosphorylation in the absence of cyclins in stationary phase?). The lack of correlation between Cak1 and pT169 is even more striking in Figure 4. In the presence of the uORF, T169 phosphorylation drops precipitously even at moderate dilutions while Cak1 remains unchanged. The correlation becomes negative in the absence of the uORF, where Cak1 levels increase in poor growth conditions while T169 phosphorylation decreases. It seems very likely that additional mechanisms are involved, but the authors do not discuss this issue in any depth or propose additional mechanisms by which T169 phosphorylation is reduced in poor growth conditions. A full exploration of the underlying mechanisms is optional here, but the paper would still benefit from a fuller acknowledgement and discussion of these questions.

Figures 5 and 6 are devoted to studies of the subtle phenotype that results from mutation of the uORF. Figure 5 suggests a decline in viability/fitness in slow growth conditions. In Figure 6, a slightly more pronounced cell cycle defect is uncovered by combining the uORF mutation with mutations in three Type 2C phosphatases (Ptc1-3) that are thought to remove T169 phosphorylation. The results are consistent with the authors' hypothesis that PP2C buffers excess T169 phosphorylation that results from higher Cak1 levels in the uORF mutant. However, given that these phosphatases might have effects on a variety of cell processes, a synthetic defect with the uORF mutation is not easily interpreted.

Minor points:

1. Kaldis et al (Cell 1996) is cited for the discovery of Cak1. The authors should also cite the other two Cak1 discovery papers, published at the same time: Thuret et al., Cell 1996 (86:565); and Espinoza et al., Science 1996 (273:1714).

2. More details are required for the methods used to carry out quantitative mass spectrometry of the phosphorylated T169 peptide in Figure 1.

2. Significance:

Significance (Required)

The discovery of a mechanism for translational control of CAK activity adds a new layer to our understanding of cell cycle control mechanisms, of interest to experts in this field and in related fields of cell biology and signaling.

3. How much time do you estimate the authors will need to complete the suggested revisions:

Estimated time to Complete Revisions (Required)

(Decision Recommendation)

Less than 1 month

4. *Review Commons* values the work of reviewers and encourages them to get credit for their work. Select 'Yes' below to register your reviewing activity at [Web of Science Reviewer Recognition Service]() (formerly Publons); note that the content of your review will not be visible on Web of Science.

Web of Science Reviewer Recognition

No

Review #3

1. Evidence, reproducibility and clarity:

Evidence, reproducibility and clarity (Required)

Summary:

In this manuscript, the authors address this question in budding yeast. Based on an old observation that Cak1 protein levels decline in stationary phase (Kaldis et al., 1998), they investigate the regulatory mechanism of such decline. They show that Cak1 concentration is regulate post-transcriptionally via an uORF in the 5' region of the transcript competing for the translation machinery. Under conditions of limiting resources (e.g. ribosomes, initiation factors) - the authors speculate - the uORF could repress Cak1 translation. Indeed,

mutating the start codon of the uORF results in a partial derepression of Cak1 under nutrient limiting conditions - though somewhat puzzling - this does not impact the state of Cdk1 T-loop phosphorylation. Consistently, the phenotype of such derepression remains relatively mild with slight impacts on viability during quiescence and in slow-proliferating populations, but is strengthened when the putative counteracting phosphatases are knocked-out in addition.

General comment:

As you will see from the more detailed comments, it was often difficult to understand and compare the measurements and results due to general depiction of the data (log scales and unclear normalization, conditions to be compared depicted on different plots) and lacking information about the replicates and number of measurements performed in the experiments. The authors should state these aspects more clearly.

Major:

- The authors state "When uORF-0 cells proliferated with glycerol as the carbon source or were in stationary phase, the levels of Cak1 were derepressed, albeit not completely, compared to uORF-35 cells." It is important to note that even in glucose the Cak1 levels are significantly higher in uORF-0. Therefore, it was difficult to judge whether the reduction is proportional or less to the initial levels for the uORF-0. The authors might want to think about a way to make this data - a crucial point of this paper - more easily comparable for the reader. Interestingly, uORF-3 shows a similar increase in glucose, but stronger repression in stationary in line with the authors arguments, but this is really not easy to see from these box blots.
- The authors should state in the figure legends how many experiments/cells were analysed (e.g. for Figure 3C, data is pooled from three biological replicates with x, y, and z number of cells).
- Figure 3C: I am a bit surprised that the authors used flow cytometry - a high-throughput method - to analyse the fluorescence but then only analysed a few cells (often <10) per condition and experiment (derived from the source data).
- Figure 5: If I understand this correctly, these are single experiments. Given the relatively mild changes (the compressed y-axes make them look larger than they are) replicates would be necessary.
- The authors use the triple ptc1,2,3 knockout strain to investigate the role of phosphatases in combination with derepressed CAK1. Given the observed phenotypes which they link to Cak1, it would be important to also demonstrate the impact on Cdk1 pT169

phosphorylation in this context using their phospho-specific antibody.
- Whereas Cdk1 is the most crucial Cak1 substrate in budding yeast, it is not the only one. Given that the derepression of Cak1 had limited impact on the phosphorylation state of Cdk1, the authors should at least discuss the possibility that some of the phenotypes are actually a consequence of Cak1 activity onto other substrates.

Minor:

- The Materials and Methods section on the mass spectrometry is insufficient. For example, from the information provided in the Material and Methods section, I had difficulties to understand what kind of normalization of the mass spectrometry data was performed (i.e. what the 'input' signal is). In case the Cdc28 and pT169-Cdc28 was measured in the same run, the input signal should be the same and the terms cancel each other out during the normalization - however, this does not seem to be the case judged from the data provided in the supplementary table. The authors should clarify, how exactly these measurements were performed and how the raw data was subsequently treated.
- Looking at the source data for Figure 1 D and reading the description of how the normalization was performed (did the authors normalize over the different conditions for the same target that doesn't seem a good idea considering that the signals vary significantly?), I was not able to recapitulate how the authors derived at the numbers shown. The authors should make more clear how exactly they treated their raw data.
- Considering that very clear effects are observed for the pT169-CDK1 antibody in different growth conditions, I am surprised to see the relatively mild reduction in signal after lambda-phosphatase treatment. It almost suggests that the phosphatase treatment was not very efficient. Maybe the authors want to consider optimizing the conditions.
- Although based on previously published data, the slow degradation of CAK after CHX addition is likely, it would have been more convincing to show an additional control with an 'unstable' protein that is degraded quickly to demonstrate that the CHX treatment was actually effective in this experiment.
- Figure 3B: I suggest replacing 'blue filter' and 'red filter' with 'mTurquoise2' and 'mRuby2', respectively.

2. Significance:

Significance (Required)

Cyclin-dependent kinases (CDKs) are important regulators of cell cycle progression. As such their activity is tightly regulated by several mechanisms including association with their respective cyclins, post-translational modifications, and inhibitory protein binding. In

order to be active, canonical CDKs require phosphorylation of a threonine in their activation loop (T-loop) mediated by a cyclin-activating kinase (Cak1 in budding yeast, CDK7 in humans - though these two kinases differ significantly). Whereas T-loop phosphorylation and CAK activity have been considered to be continues for a long time (e.g. Kaldis et al., 1998; Tassan et al. 1994), CDK T-loop phosphorylations have more recently emerged as more regulated than initially presumed (e.g., Schachter et al. 2013, Mol Cell; Kirova et al. 2022 Dev Cell). Furthermore, CDK7 has recently emerged as an interesting target for cancer therapy being frequently overexpressed in cancer with several inhibitors in preclinical and clinical trials. Nevertheless, it has remained curious and unresolved, why CAK activity itself seems to be so little regulated.

General assessment:

The regulation of Cak1 by the uORF and the direct tuning of the expression levels by the growth rate via an uORF for a major cell cycle regulator is convincing and will be interesting to the (yeast) cell cycle community. However, the phenotypic analyses are technically less convincing, and I would encourage the authors to improve this part of the manuscript before publication. Furthermore, the manuscript can be strengthened by more clearly stating what has been done and improving how results are depicted.

Field of expertise: Cell cycle, CDK1 regulation, quantitative biology

3. How much time do you estimate the authors will need to complete the suggested revisions:

Estimated time to Complete Revisions (Required)

(Decision Recommendation)

Between 1 and 3 months

4. *Review Commons* values the work of reviewers and encourages them to get credit for their work. Select 'Yes' below to register your reviewing activity at [Web of Science Reviewer Recognition Service](#) (formerly Publons); note that the content of your review will not be visible on Web of Science.

Web of Science Reviewer Recognition

Yes

We are very grateful to all three reviewers for their thorough reading of the manuscript and highly constructive suggestions. We have addressed all of their comments, which included performing several new experiments and making extensive text edits. Our point-by-point response follows.

Reviewer #1 (Evidence, reproducibility and clarity (Required)):

The study "Translational control of CAK and Cdk T-loop phosphorylation in response to growth in yeast" by Blank et al investigates the regulation of the CDK activating Kinase (CAK) in yeast. This conserved kinase phosphorylates a conserved residue on the main cell cycle kinase Cdk1, which is threonine T169 in yeast. While it has long been known that the phosphorylation is required for full Cdk activity, little is known (particularly in yeast) how this site is regulated and what the physiological relevance of this regulation would be. This is particularly interesting since this phosphorylation does not seem to be regulated during the cell cycle per se.
Here, the authors show that the phosphorylation of T169 is nutrient/growth rate dependent. Both the fraction of phosphorylated T169 and the abundance of CAK itself are regulated with nutrients/growth rate. The authors show that CAK is mainly regulated postranscriptionally by a uORF directly upstream of the CAK ATG. This suggests that the levels of CAK are regulated by ribosome availability (though this is only inferred and not directly demonstrated), i.e. biosynthetic capacity of the cell. Surprisingly though, the abundance of CAK does not correlate well with the amount of T169 phosphorylation, implying additional layers of regulation. The authors look for this at the level of dephosphorylation by Ptcs and present some evidence that this is the case. More convincing I find the evidence that dysregulated CAK prevents ordered entry into stationary phase/quiescence.
Overall, I find the results very intriguing and in sum mostly convincing. However, I have some major issues with data representation and also transparency as to how data was generated and what individual data points mean. Although my list of major points is long, most of them can be addressed by replotting existing data or clarifying things in the text. Additionally, I have some optional suggestions on how to connect some of the dots and make the story more coherent, depending on the timeline and journal the authors are aiming for, see significance statement.

Major points:
1. Box plots are meaningful only for larger datasets, I would suggest at least 20 datapoints. Box plots with less than five datapoints are by definition mathematical nonsense! Please replace all boxplots with few data points by something reasonably, such as showing the individual points as dots and the average as a line etc.

RESPONSE: Done. As suggested, the box plots with less than 5 points have now been converted to strip plots.

2. Comparison of uORF 16 and 35 is missing. It is of course very unfortunate that the protein required N-terminal tagging, sometimes you just have to deal with what is possible. But, I dont see anywhere a thorough comparison of the uORF 16 and 35. The authors state in the methods that they dont see any difference in the growth rates and budding. But what about fraction of

T169 phosphorylation? What about viability in stationary phase etc.? The authors try to correlate the biochemical data with the phenotype, but do this with two different strains (that even have slightly different backgrounds if I interpret the methods correctly).

RESPONSE: Good points. We confirmed that T169 phosphorylation is the same in uORF-16 and uORF-35 cells (new Figure EV5). We also compared the viability of uORF-35 vs uORF-16 cells, as suggested. After 10 days in the stationary phase, the viability of the two strains was indistinguishable.

To reflect the above, we added the following in the text: "Furthermore, *uORF-35* cells (strain MSP258) displayed T169 phosphorylation levels identical to those of *uORF-16* cells (Figure EV5), and their viability was the same after 10d in the stationary phase (93.5% ± 6.2 vs. 92.6% ± 6.0, respectively; see Source Data for Figure 5A)".

3. Figure 1: B-> exchange box plot. C-> I dont see how the pRps6 AB validates anything. Assuming you have a working phosphatase, then your AB has a residual affinity towards the unphosphorylated site. Just admit that you have some % background and fine. D. Again, replace box plots.

RESPONSE: Box plots replaced with strip plots. As for the Rps6, we used it as a comparison. Although residual affinity of these antibodies for their non-phosphorylated epitopes is a formal possibility, we think it is an unlikely explanation because there are settings (stationary phase or low chemostat dilution rates) where there is no phospho-signal while Cdc28 remains abundant. Nonetheless, to address the reviewer's point we added the following (end of the 1st paragraph of the results): "While incomplete dephosphorylation is the most likely explanation for the residual signal, it is also possible that some residual affinity of these antibodies for their non-phosphorylated target may remain."

4. Figure 2: A-> the overview figure is in principle great, but I found the illustration confusing. 2B-> Again boxplots are non-sensical here. Maybe plotting the three strains in one plot rather than (or in addition to) comparing the strain to itself under different conditions would be helpful, especially to compare with EV2A.

RESPONSE: The illustration (2A) is an accurate depiction of the locus in the different strains. We are not sure why it is confusing and, if so, how to fix it.

Box plots were replaced with strip plots.

We do not think the comparison the reviewer proposed will be better. Our focus is how protein signals respond to growth changes. The comparison relied on 'expressed ratios', instead of other normalization methods. It has been used widely in the cell cycle field, in time series. It properly normalizes the signal of a given protein in different conditions on the same gel, independent of the signal of other proteins. It effectively turns data into

"fold-change" data, avoiding inter-gel variability. Doing what the reviewer suggested, with strains run on different gels, would re-introduce such bias and noise.

To clarify, we added the following (end of the 2nd paragraph of the results): "This internal mean normalization, the 'expressed ratio' method (Spellman et al, 1998), displays protein abundances in the different conditions we tested. Unlike other normalizations (e.g., to a loading control), this approach calibrates each protein's signal relative to its average across the experimental series on a single gel. It accounts for gel-to-gel and exposure differences among independent replicates. The Log2 transformation shows protein levels with equal weight around a central baseline (zero), yielding a symmetrical, biologically intuitive view of protein level fluctuations across conditions."

5. Figure EV2: It is not stated here or in the methods how these experiments were performed. Are these growth experiments in shake flasks? Or in multiwell-plates? If multi-well plates, were the wild-type and mutant paired, or was everything done on a single day? How many replicates were performed? When was the size/budding data taken? Once per experiment or is it an average across exponential phase?

RESPONSE: These pieces of information were in the Source Data_FigureEV2A file. In short, it was from multiple independent experiments over a 2-month period, involving at least two different people and several different batches of media. We added the following information in the methods (in the Doubling time measurements in batch cultures section): "...from shake flask cultures at 30°C sampled at regular intervals over a day. From the same measurements, the budding index and cell size was also measured."

6. EV2: Why is the data so extremely noisy? There seems to be up to 50% difference in growth rate even in the wildtype. This makes no sense. Maybe a simple OD-based growth assay would be better than manual cell counting (why was this done?) There seems to be a clear overall trend that the uORF-0 grows slightly slower, but this is masked by the noise in the data.

RESPONSE: As we described in the previous point, we pooled measurements done over many independent experiments, by different people and also from different batches of media. In this context, the variability in these experiments is not surprising. The trend the reviewer points to is just that, a trend that did not reach statistical significance. Please also note that similar experiments done with a plate reader in FigureEV4 (albeit in the ptc2,3 background) also showed no significant difference in doubling time between wild type and the uORF mutant. Likewise, in a new experiment monitoring cells after they exit the stationary phase, once they reached the exponential phase of growth there was again no significant difference in their specific growth rate (see new Figure 5C).

7. Figure 3. This is in principle a very powerful assay to prove the point. It might have been better to put both reporters on one plasmid for ratiometric analysis. A. Labelling reporter 2 CAK1-mRuby in the figure gives the impression this is a fusion protein, but I am assuming it is just mRuby, or mRuby with just a small N-terminal section of CAK. How long is the actual uORF

in the mRuby construct? B. The localization of the Turquoise looks weird, any explanation? C. It is a bit unclear how many replicates where done and what the induvial data points refer to. Are these averages of x number of cells in y-number of flow cytometry experiments? The y-axis makes no sense. What ratio are you referring to? Wouldnt it make sense to show the mRuby to mTurquoise ratio per cell instead of normalizing within the fluorophore? Why is there no difference between glucose and glycerol in reporter 2? Isnt that one of the key points about the idea how the uORF works?

RESPONSE: We see the confusion. Our naming was not ideal. It is the fluorescent proteins expressed from the *uORF* ATG for mTurquoise or the *CAK1* ATG for mRuby. We changed the names of these plasmids to: *uORF*::mTurquoise2 (Reporter 1), *uORF*+ *CAK1*::mRuby2 (Reporter 2), and *uORF-0 CAK1*::mRuby2 (Reporter 3). Hopefully that makes it clear. As for the localization pattern we have no explanation but we are not sure it means anything. The level of fluorescence is very high (they are 2micron plasmids).

In Figure 3C, each data point is from an independent culture. They were also repeated on separate days. All the data were then pooled and displayed. All this information is in the Source Data Figure3C file. To account for the variability in the obtained values (different media, dates, etc) we normalized as before, using the internal mean/expressed ratio approach. We maintain that this is the least biased approach. As for normalizing using the ratio of the two colors, we argue that this would be of lesser value. The point of the figure is not to show how the two colors compare to each other, but how each color changes in the two media conditions. It also shows the effect of the uORF mutation, and the fact that the ATG of the uORF can drive expression of a gene product.

8. Figure EV3: B - Are these diploid cells? Why?

RESPONSE: Yes they are diploid. There was no particularly significant reason for it, other than a possible ease in imaging larger cells (diploid) vs. smaller ones (haploid). We do not see why this would affect any of the conclusions.

9. Figure 4: The authors completely brush over the fact that in the uORF 0, the V5-Cak actually decreases with growth rate. In fact, it decreases considerably more, than the Cak increases in the uORF35. What could this mean for regulation?

RESPONSE: In wild type cells V5-Cak1 decreases with growth rate. Indeed, as the reviewer pointed out, the pattern appears to be reversed in the uORF-0 cells but only in nitrogen-limited cultures, not in carbon-limited ones, where it remains flat. We now state the observation the reviewer noted with a sentence in the text: "If anything, in uORF-0 cells under nitrogen limitation Cak1 levels might be reduced at higher dilution rates."

We are not sure about what it means. It is possible that nitrogen limitation is a more sensitized condition than glucose limitation (the uORF-0 effects on T169 phosphorylation also appear a bit more pronounced in nitrogen-limited cultures, but only slightly so). We

wish to point out, however, that the key result is clear: the uORF is responsible for the decrease in V5-Cak1 levels in low growth rates in wild type cells.

10. Figure 4C: I understand that running these chemostats is a lot of work so you cannot run all of them in replicates. However, it might be worth looking at the critical dilution where the two strains differ and repeating those to see if the differences in the curves are noise or if the strains actually have a different "critical point".

RESPONSE: The data we show are not from single experiments. Adding more repetitions to possibly further narrow the dilution rate at which T169 phosphorylation drops precipitously may bring slightly higher resolution but in the end it will offer little to justify the substantial time and effort of these experiments. None of the conclusions of this work will be altered in any significant way.

11. Figure 5: A- The difference in viability is by far the most convincing phenotype shown. Yet, it seems to come from only one replicate? This is a reasonably simple experiment to do, I would recommend triplicates and then putting more emphasis on this finding, see below. B. Here, it seems there was also only one replicate each? Maybe replicate just the carbon limited experiment? At higher dilution rate, the mutant seems to have an advantage. Do the authors have any thoughts on this?

RESPONSE: We now repeated the viability experiment, with 6 independent replicates for each strain, carried over a longer period of time, and analyzed it with robust mixed-effects statistics, due to the longitudinal measurements. The results are more convincing than before and highly significant (p=7.41E-5). The experiments are shown in the new Figure 5A.

We also added additional experiments to the carbon-limited chemostat competition data (see updated Figure 5B) as suggested. The conclusions remain the same. We also note that there was no advantage of the mutant at the higher dilution rate.

12. Figure 6: The authors should show the T169 phosphorylation in these mutants. Does this somehow correlate with the G1 fraction? There does seem to be something going on with Ptc, but I find the data only semi-convincing. The triple mutant per se likely has all kinds of defects. Working with Phosphatases is tricky. But maybe trying to find a phenotype in steady-state growth is not what the authors should be aiming for, see significance.

RESPONSE: We now added exciting new data, showing that T169 phosphorylation is markedly elevated in ptc1,2,3 cells lacking the CAK1 uORF compared to ptc1,2,3 cells with the uORF (see new Figure 6C). We describe them in the text as follows:

"To determine whether these cell cycle defects arise from altered Cdk1 regulation, we measured T169 phosphorylation levels in the *uORF-16, ptc1,2,3Δ* and *uORF-0, ptc1,2,3Δ* cells. Strikingly, the *uORF-0* mutation increased activating phosphorylation in glucose-

grown cells, and this effect was amplified in glycerol (Figure 6C, xxxx). This surge in T169 phosphorylation explains the accelerated START and delayed mitotic exit observed in this sensitized background (Figure 6A,B). By unmasking these phenotypes in cells lacking Ptc1-3, these data suggest that the *CAK1* uORF can exert a potent influence on cell cycle progression. These results strongly suggest that protein phosphatases act as a buffer, counteracting the elevated Cak1 abundance caused by the loss of uORF-mediated translational control."

13. Figure 7. Both the graphical abstract and Figure 7 are overstating the findings. "Schematic of our findings" is certainly not true. Maybe better "putative model of G1/S control through translation". Formally, the authors did nothing to show that it has to do with ribosome availability. Also, I am not sure if it has been shown that the "freak" cyclin Cln3 actually needs pT169 phosphorylation. What also doesn't become clear in the picture or anywhere else is that CAK and Cln3 operate on different timescales due to their very different protein stability.

RESPONSE: We changed the title of Figure 7 to: "A model of G1/S control through translation".

That ribosome availability is regulated as a function of growth has been known for decades and confirmed with newer technologies in recent years. We now provide the citations as requested.

As to whether Cln3/Cdc28 requires T169 phosphorylation the reviewer is formally correct, since it has been very difficult to measure Cln3/Cdc28 activity. Hence, we added in the legend the sentence "It is also assumed that the activity of Cln3/Cdc28 requires T169 phosphorylation."

We note, however, that other proteins (Ringo/Speedy) known to activate mammalian Cdks without T-loop phosphorylation have no yeast counterparts.

14. The discussion is extremely superficial and does not do a good job of connecting the findings to the literature (the authors cite a total of four papers, three of which are their own).

RESPONSE: Fair point. We were pressed for character limitations for our intended journal, but that is not a good excuse. We expanded the discussion substantially. To our defense, we also note that 'our own citations' were just 2 reviews, including a recent one about CAK control, where we cite all the primary literature.

15. In general, the reference list is very self-centred. To a certain extend this is normal, but I find this one extreme.

RESPONSE: Please see our response to the previous point.

There is not a single paper cited that shows the connection of ribosome content/growth rate/growth condition.

RESPONSE: Corrected. Please see our response to point #13, above.

Also, there has been so much recent work on connecting nutrient signalling/growth control to cell cycle progression. It would have been so much nicer to see a discussion of these results in a bigger context.

RESPONSE: We understand the reviewer's point. We note, however, that our focus is on the role of the T-loop phosphorylation, not on an expansive review of nutrient signalling/growth control. Nonetheless, the expanded discussion hopefully addresses the reviewer's concern.

16. Methods: Some of the methods are very detailed, some of them completely lack critical information. For example, how were cells grown (shakeflasks, mutliwell-plates...?)?

RESPONSE: We now added this information, please see our response to point #5, above.

Also, "We gave it to the facility" is NOT a method. I dont know if this was laziness or sloppiness, but I am sure the facility can provide a proper protocol.

RESPONSE: Agreed. The full description is now in the Methods.

17. Generally, the proteomics data is not well accessible. As far as I can tell, the raw data was not deposited on PRIDE etc. And the "source data" provided for Fig 1B is a non-annotated csv that does not contain the detected peptides or any other information typically provided in proteomics experiments. Or did I miss an additional table somewhere?

RESPONSE: Apologies for this. There must have been a server error somewhere. We had uploaded a separate comprehensive file (Dataset EV1), listing all the proteins and phosphopeptides we identified. We will ensure this file is accessible to the reviewers.

Minor Points:
1. EV figures dont map 1:1 to main figures. This of course depends on the journal, but I find it confusing.

RESPONSE: Thanks for alerting us to this. It was our impression that the extended view and main figure numbers don't have to match. If that is not the case, we will follow the Journal's instructions.

2. Figure 1D and 2B-> y-axis label is confusing. Call it normalized abundance or something like that. What is an "expressed ratio"? Apart from that, you later show that it is not expression but translation.

RESPONSE: We changed the y-axis labels in Figures 1D, 2B, and 3C to "normalized levels" from "expressed ratio".

3. EV3: "Histograms show cell counts", no it doesn't, it shows fractions. There is generally a lot of imprecision in the figure legends.

RESPONSE: Corrected, to "fraction of cell counts" in the legend description. Thank you.

4. In many figures the position of the axis labelling is weird.

RESPONSE: We do not understand this comment. All figures were done with optimal aspect ratios and standard placements of axis labelling (R language lattice defaults).

Reviewer #1 (Significance (Required)):

I think the observations from this study are highly significant to the yeast cell cycle field, and also relevant for the entire cell cycle/ growth control field. I am sure the study is publishable within its current scope in a decent journal if the technical issues are fixed.
However, in its current state there are so many loose ends that would be reasonably easy to connect. It is a pity that the authors dont really try to develop a model of what is going on and why this regulation is important. Clearly, in steady state the cell does not seem to particularly care about the precise CAK levels and the T169 levels are mainly controlled by phosphatase activity. So why would a protein with a half-life of 4 hours be translationally regulated? It must operate on a completely different logic than Cln3, which has a half-life of only a few minutes.

RESPONSE: As suggested, we added the following in the text:
"While Cak1 is a very stable protein (see Figure EV1 and (Christiano et al, 2014; Kaldis et al, 1998a)), Cln3 is highly unstable (Figure EV1 and (Yaglom et al, 1995; Cross & Blake, 1993; Tyers et al, 1992)). This kinetic difference likely reflects distinct physiological roles. Several reports have questioned the initial assumption (Tyers et al, 1993) that Cln3 abundance does not vary in the cell cycle. Instead, Cln3 levels (Litsios et al, 2019; Sommer et al, 2021; Thorburn et al, 2013; Zapata et al, 2014; Landry et al, 2012; Litsios et al, 2022) and protein synthesis rates (Takhaveev et al, 2023; Litsios et al, 2024) appear to be dynamic in the cell cycle. Cln3's instability makes it better suited to act quickly as a real-time indicator of protein synthesis capacity. In contrast, because Cak1 is not rapidly degraded, its uORF-mediated repression slowly lowers total Cdk activity, not just G1/Cdk, resetting the cell to a much lower proliferative baseline to optimize survival during prolonged nutrient limitation."

The most obvious phenotype that the authors observe is a failure to enter stationary phase properly. So why not investigate this a bit more closely? Just run growth curves in shake flask and monitor growth, CAK levels, T169 phosphorylation etc over the transition from glucose to ethanol to stationary phase. Also, one main advantage of a translation-based regulation rather

than a transcriptional regulation would be a much faster re-activation upon exit from stationary phase. Maybe that is something to look at?

RESPONSE: We did not do the suggested diauxic shift experiment for two reasons: First, we do not see how the glucose to ethanol transition would offer different insights from the extensive data we already collected and showed comparing steady-state glucose vs. glycerol cultures. Second, Cak1 is very stable (see new data in Figure EV1), with a half-life that exceeds the time it takes for this transition. However, we did do the second portion of the suggested experiment, following cells as they exited the stationary phase and re-entered the cell cycle (thank you for the suggestion). These new results are shown in the revised Figure 5. We describe the results in the text, as follows:

"Lastly, we also monitored *uORF-0* cells as they exited quiescence and reëntered the mitotic cell cycle. After 1w in the stationary phase, we placed cells in fresh, rich media (YPD) and measured cell numbers, size, budding, and levels of T169 phosphorylation at regular intervals. We found that *uORF-0* cells exhibited a 1-1.5h delay reaching exponential cell proliferation kinetics (Figure 5C). Once the culture reached the exponential phase of growth, however, the specific rate of growth was similar to *uORF-35* cells (p=0.286, ANCOVA testing for homogeneity of regression slopes). During the lag phase, the cell size of *uORF-0* cells was slightly smaller than that of uORF-35 cells (Figure 5D), but they budded with the same kinetics as *uORF-35* cells (Figure 5E), suggesting that the delay in reaching the exponential phase of growth was likely the result of delayed exit from mitosis during the initial reëntry into mitotic cycling. We also monitored the abundance of V5-Cak1, pT169, Cdc28, and Pgk1 during these experiments by immunoblotting (Figure 5F). V5-Cak1 levels rose faster in *uORF-0* cells (Figure 5F, left panels), validating the inhibitory role of the uORF. In line with this rapid Cak1 accumulation, T169 phosphorylation trended higher 1–2 hours after refeeding (Figure 5F, second panels). While our statistical power was limited, Cdk activation was clearly not delayed. Together, these findings indicate that the slower quiescence exit of *uORF-0* cells is driven by a delayed exit from mitosis, rather than impaired Cdk activation."

We also note that since Cdk activation requires not only T169 phosphorylation but also cyclin binding, it likely explains why uORF-0 cells did not accelerate completion of the G1/S transition as they exited quiescence.

Reviewer #2 (Evidence, reproducibility and clarity (Required)):

The authors have discovered an interesting regulatory mechanism governing the cellular levels of Cak1, the essential Cdk-activating kinase of budding yeast. Cak1 phosphorylates T169 in the major Cdk, Cdc28, resulting in full activation of Cdc28 and cell-cycle progression. Surprisingly, and despite its central importance, Cak1 is not believed to be regulated during the cell cycle - apart from previous evidence (Kaldis et al 1998) that Cak1 levels decline when proliferation is inhibited by starvation (stationary phase). The current paper revisits the question of Cak1 regulation and uncovers evidence that growth conditions govern Cak1 levels by changing its rate of translation. An upstream ORF appears to reduce Cak1 translation in slow growth conditions. The experiments are generally convincing and clear, and a variety of rigorous approaches have been used.

The paper begins in Figure 1 with clear evidence that T169 phosphorylation on Cdc28 declines in poor growth conditions and is essentially absent in stationary phase. This decline in pT169 is accompanied by a decline in Cak1 levels. The authors then uncover the mechanism that reduces Cak1 levels. After ruling out some mechanisms (no changes in Cak1 mRNA abundance or Cak1 stability), they pursue the role of a small uORF that starts just upstream of the Cak1 start codon. The results in Figure 2 demonstrate the mutation of the uORF start codon increases Cak1 levels significantly in poor growth conditions, and even a very short uORF reduces Cak1 levels, suggesting that translation initiation at the upstream site reduces Cak1 translation in poor growth conditions. Experiments with fluorescent reporter genes in Figure 3 further demonstrate the inhibitory effect of the uORF on translation. In Figure 4, chemostat experiments under various growth conditions further support these conclusions. All these experiments provide a convincing case that the uORF is responsible for reduced Cak1 levels in poor growth conditions.

The story becomes less straightforward when the authors analyze T169 phosphorylation in these experiments. There is a significant lack of correlation between Cak1 levels and T169 phosphorylation. In Figure 2, mutation of the uORF restores Cak1 levels in stationary phase but does not affect T169 phosphorylation, suggesting that Cak1 is not the sole determinant of pT169 levels, and perhaps that some other mechanism is also suppressing phosphorylation (Activation of a phosphatase? More rapid Cdc28 dephosphorylation in the absence of cyclins in stationary phase?). The lack of correlation between Cak1 and pT169 is even more striking in Figure 4. In the presence of the uORF, T169 phosphorylation drops precipitously even at moderate dilutions while Cak1 remains unchanged. The correlation becomes negative in the absence of the uORF, where Cak1 levels increase in poor growth conditions while T169 phosphorylation decreases. It seems very likely that additional mechanisms are involved, but the authors do not discuss this issue in any depth or propose additional mechanisms by which T169 phosphorylation is reduced in poor growth conditions. A full exploration of the underlying mechanisms is optional here, but the paper would still benefit from a fuller acknowledgement and discussion of these questions.

RESPONSE: Excellent points. We added the following in the Discussion:

“Our data suggest the relationship between Cak1 abundance and the level of T169-Cdc28 phosphorylation is non-linear. While eliminating the uORF restored Cak1 levels in the stationary phase, it failed to restore T169 phosphorylation (Figure 2). In chemostat cultures, T169 phosphorylation dropped precipitously at low dilution rates even when Cak1 levels were stabilized or increased by the absence of the uORF (Figure 4). These results suggest that Cak1 abundance is not the only determinant of T-loop phosphorylation. It is highly likely that "eraser" mechanisms, such as the activity of type 2C protein phosphatases (Ptcs), become dominant in poor growth conditions. Our finding that cells lacking several PTC genes become significantly more sensitive to Cak1 levels supports this buffering role for phosphatases.

Other processes may also account for the non-linear relation between Cak1 levels and T169 phosphorylation. In the stationary phase or in chemostats at low dilution rates, cyclins will be absent or at exceedingly low levels, and most of the Cdk would exist as a monomer. Although Cak1 targets the Cdk monomer (Kaldis et al, 1998b; Tsakraklides & Solomon, 2002), in certain cases, it can preferentially target Cdk-Cyclin dimers (López-Hernández & Vázquez-Ramos, 2023). In poor growth conditions the Cdk monomer may be a poorer substrate for Cak1 or more susceptible to dephosphorylation by phosphatases. In such a scenario, the uORF-mediated control of Cak1 synthesis contributes to the maximum level of Cdk activation. Phosphatases and cyclin availability may provide an additional fail-safe to ensure T169 dephosphorylation when growth is unfavorable.

The data we described contribute to a unifying view of CAK across all eukaryotes. Observations in animal cells, such as the higher threshold of T-loop Cdk phosphorylation required for the G1/S transition in response to extracellular signals and the heightened involvement of counteracting phosphatases at that proliferative transition (Schachter et al, 2013; Blank et al, 2025), point to a general and conserved role for CAK in safeguarding cells before they commit to a new round of division. More generally, recent studies in mammals also suggest that Cdk T-loop phosphorylations are subject to greater regulatory control than previously assumed (Schachter et al, 2013; Kirova et al, 2022). Lastly, human CAK has been identified as a promising target for cancer therapy, and several CAK inhibitors are being evaluated in clinical trials (NCT04247126, NCT03134638, NCT05394103), underscoring the interest and significance of CAK-mediated control of cell proliferation.”

Figures 5 and 6 are devoted to studies of the subtle phenotype that results from mutation of the uORF. Figure 5 suggests a decline in viability/fitness in slow growth conditions. In Figure 6, a slightly more pronounced cell cycle defect is uncovered by combining the uORF mutation with mutations in three Type 2C phosphatases (Ptc1-3) that are thought to remove T169 phosphorylation. The results are consistent with the authors' hypothesis that PP2C buffers excess T169 phosphorylation that results from higher Cak1 levels in the uORF mutant. However, given that these phosphatases might have effects on a variety of cell processes, a synthetic defect with the uORF mutation is not easily interpreted.

RESPONSE: We see the reviewer’s point. Nonetheless, these phosphatase mutants do provide a sensitized-enough background for the uORF phenotype to manifest itself. We now added the following (last paragraph of this section in the results): “While these

phosphatases influence multiple cellular processes, their absence creates a sensitized context that exposes the uORF's impact on cell cycle progression."

We also wish to point out that the phenotype of START acceleration in the uORF mutant is <u>not</u> subtle. In our hands, it is stronger than most other well-known mutants that accelerate Start, including the dominant CLN3-1 mutant.

Minor points:

1. Kaldis et al (Cell 1996) is cited for the discovery of Cak1. The authors should also cite the other two Cak1 discovery papers, published at the same time: Thuret et al., Cell 1996 (86:565); and Espinoza et al., Science 1996 (273:1714).

RESPONSE: Corrected. Citations were added.

2. More details are required for the methods used to carry out quantitative mass spectrometry of the phosphorylated T169 peptide in Figure 1.

RESPONSE: Corrected. The description is now shown in the Methods. Please see also our response to Reviewer 1, point #17, regarding the complete dataset of all identified peptides from this experiment.

Reviewer #2 (Significance (Required)):

The discovery of a mechanism for translational control of CAK activity adds a new layer to our understanding of cell cycle control mechanisms, of interest to experts in this field and in related fields of cell biology and signaling.

Reviewer #3 (Evidence, reproducibility and clarity (Required)):

Summary:
In this manuscript, the authors address this question in budding yeast. Based on an old observation that Cak1 protein levels decline in stationary phase (Kaldis et al., 1998), they investigate the regulatory mechanism of such decline. They show that Cak1 concentration is regulate post-transcriptionally via an uORF in the 5' region of the transcript competing for the translation machinery. Under conditions of limiting resources (e.g. ribosomes, initiation factors) - the authors speculate - the uORF could repress Cak1 translation. Indeed, mutating the start codon of the uORF results in a partial derepression of Cak1 under nutrient limiting conditions - though somewhat puzzling - this does not impact the state of Cdk1 T-loop phosphorylation. Consistently, the phenotype of such derepression remains relatively mild with slight impacts on viability during quiescence and in slow-proliferating populations, but is strengthened when the putative counteracting phosphatases are knocked-out in addition.

General comment:
As you will see from the more detailed comments, it was often difficult to understand and compare the measurements and results due to general depiction of the data (log scales and unclear normalization, conditions to be compared depicted on different plots) and lacking information about the replicates and number of measurements performed in the experiments. The authors should state these aspects more clearly.

Mayor:
- The authors state "When uORF-0 cells proliferated with glycerol as the carbon source or were in stationary phase, the levels of Cak1 were derepressed, albeit not completely, compared to uORF-35 cells." It is important to note that even in glucose the Cak1 levels are significantly higher in uORF-0. Therefore, it was difficult to judge whether the reduction is proportional or less to the initial levels for the uORF-0. The authors might want to think about a way to make this data - a crucial point of this paper - more easily comparable for the reader. Interestingly, uORF-3 shows a similar increase in glucose, but stronger repression in stationary in line with the authors arguments, but this is really not easy to see from these box blots.

RESPONSE: We do not quite understand the confusion. Looking at our data in Figure 2B (now shown as strip plots, instead of box plots), in uORF-35 cells the difference in V5-Cak1 levels is ~3-4-fold between glucose and glycerol (see bottom left panel). This difference is reduced to <0.5 fold in the uORF-0 mutant (middle left panel), but it remains at ~3-fold in the uORF-3 mutant (top left panel). These results support our conclusions that in the uORF mutant in glycerol Cak1 expression is de-repressed.

Perhaps another way to look at the data is that V5-Cak1 levels are similar in all conditions in uORF-0 cells, but show differences (highest in glucose, lower in glycerol, and much lower in stationary phase) in uORF-35 and uORF-3 mutants.

- The authors should state in the figure legends how many experiments/cells were analysed (e.g. for Figure 3C, data is pooled from three biological replicates with x, y, and z number of cells).

RESPONSE: The number of independent experiments is reflected in the data points. We now added the following in the legend: "Every datapoint is from a separate experiment scoring thousands of cells in each case."

- Figure 3C: I am a bit surprised that the authors used flow cytometry - a high-throughput method - to analyse the fluorescence but then only analysed a few cells (often <10) per condition and experiment (derived from the source data).

RESPONSE: As mentioned above, fluorescence per cell was scored from thousands of cells in multiple independent experiments. Each datapoint is not a single cell, but an independent experiment.

- Figure 5: If I understand this correctly, these are single experiments. Given the relatively mild changes (the compressed y-axes make them look larger than they are) replicates would be necessary.

RESPONSE: We now repeated the viability experiment, with 6 independent replicates for each strain, carried over a longer period of time, and analyzed with robust mixed-effects statistics, due to the longitudinal measurements. The results are more convincing than before and highly significant (p=7.41E-5). It is shown in the new Figure 5A.

- The authors use the triple ptc1,2,3 knockout strain to investigate the role of phosphatases in combination with derepressed CAK1. Given the observed phenotypes which they link to Cak1, it would be important to also demonstrate the impact on Cdk1 pT169 phosphorylation in this context using their phospho-specific antibody.

RESPONSE: Excellent suggestion. This is the same response as for point #12 of Reviewer 1. We now added exciting new data, showing that T169 phosphorylation is markedly elevated in ptc1,2,3 cells lacking the CAK1 uORF compared to ptc1,2,3 cells with the uORF (see new Figure 6C). We describe them in the text as follows:

"To determine whether these cell cycle defects arise from altered Cdk1 regulation, we measured T169 phosphorylation levels in the *uORF-16, ptc1,2,3Δ* and *uORF-0, ptc1,2,3Δ* cells. Strikingly, the *uORF-0* mutation increased activating phosphorylation in glucose-grown cells, and this effect was amplified in glycerol (Figure 6C, xxxx). This surge in T169 phosphorylation explains the accelerated START and delayed mitotic exit observed in this sensitized background (Figure 6A,B). By unmasking these phenotypes in cells lacking Ptc1-3, these data suggest that the *CAK1* uORF can exert a potent influence on cell cycle progression. These results strongly suggest that protein phosphatases act as

a buffer, counteracting the elevated Cak1 abundance caused by the loss of uORF-mediated translational control."

- Whereas Cdk1 is the most crucial Cak1 substrate in budding yeast, it is not the only one. Given that the derepression of Cak1 had limited impact on the phosphorylation state of Cdk1, the authors should at least discuss the possibility that some of the phenotypes are actually a consequence of Cak1 activity onto other substrates.

RESPONSE: Good point. We added the following in the Discussion: "Furthermore, while our emphasis here was on the T-loop phosphorylation of Cdc28, the only essential function of Cak1, it is known that Cak1 targets additional proteins. Hence, it is possible that the growth-dependent regulation of Cak1 through the uORF affects other processes as well, coupling them to cellular biosynthesis, contributing to the phenotypes we observed."

Minor:
- The Materials and Methods section on the mass spectrometry is insufficient. For example, from the information provided in the Material and Methods section, I had difficulties to understand what kind of normalization of the mass spectrometry data was performed (i.e. what the 'input' signal is). In case the Cdc28 and pT169-Cdc28 was measured in the same run, the input signal should be the same and the terms cancel each other out during the normalization - however, this does not seem to be the case judged from the data provided in the supplementary table. The authors should clarify, how exactly these measurements were performed and how the raw data was subsequently treated.

RESPONSE: We have now added the detailed mass spectrometry methodology. We also better explain how the ratio was determined. Note that the total abundance of Cdc28 (i.e the denominator in the ratio of pT169 : Cdc28) was from the sum of all Cdc28 peptides identified in this experiment, as shown in Dataset EV1.

- Looking at the source data for Figure 1 D and reading the description of how the normalization was performed (did the authors normalize over the different conditions for the same target that doesn't seem a good idea considering that the signals vary significantly?), I was not able to recapitulate how the authors derived at the numbers shown. The authors should make more clear how exactly they treated their raw data.

RESPONSE: This is the same response as for point #4 of Reviewer 1. The comparison here relied on 'expressed ratios', instead of other normalization methods. It has been used widely in the cell cycle field, in time series. It properly normalizes the signal of a given protein in different conditions on the same gel, independent of the signal of other proteins. To clarify, we added the following (end of the 2nd paragraph of the results): "This internal mean normalization, the 'expressed ratio' method (Spellman et al, 1998), displays protein abundances in the different conditions we tested. Unlike other normalizations (e.g., to a loading control), this approach calibrates each protein's signal

relative to its average across the experimental series on a single gel. It accounts for gel-to-gel and exposure differences among independent replicates. The Log2 transformation shows protein levels with equal weight around a central baseline (zero), yielding a symmetrical, biologically intuitive view of protein level fluctuations across conditions."

- Considering that very clear effects are observed for the pT169-CDK1 antibody in different growth conditions, I am surprised to see the relatively mild reduction in signal after lambda-phosphatase treatment. It almost suggests that the phosphatase treatment was not very efficient. Maybe the authors want to consider optimizing the conditions.

RESPONSE: This is the same response as for point #3 of Reviewer 1. We added the following (end of the 1st paragraph of the results): "While incomplete dephosphorylation is the most likely explanation for the residual signal, it is also possible that some residual affinity of these antibodies for their non-phosphorylated target may remain."

- Although based on previously published data, the slow degradation of CAK after CHX addition is likely, it would have been more convincing to show an additional control with an 'unstable' protein that is degraded quickly to demonstrate that the CHX treatment was actually effective in this experiment.

RESPONSE: Following the reviewer's suggestion, we repeated this experiment (now shown in the expanded Figure EV1) using strains expressing at endogenous levels V5-Cak1 or Cln3-13Myc, to query the stability of these proteins after addition of CHX (100µg/mL). We describe the results in the text as follows:

"We extended these observations using strains expressing at endogenous levels V5-Cak1 (Figure EV1D) or Cln3-13Myc (Figure EV1E), to query the stability of these proteins after addition of CHX (100µg/mL). Cln3 is a G1 cyclin known to be extremely unstable, with a half-life of a few minutes (Yaglom *et al*, 1995), and served as the control. We obtained a 2.3m estimated half-life for Cln3-13Myc, but little degradation for V5-Cak1, with an estimated half-life of 14.9h (Figure EV1F). These results argue against Cak1 destabilization as the primary mechanism explaining the observed differences in Cak1 protein abundance in glucose versus the poorer glycerol media. We note that the general notion that Cak1 is a very stable protein agrees with prior studies (Christiano *et al*, 2014; Kaldis *et al*, 1998a), and with Cak1's listed half-life (16.9h) on the Saccharomyces Genome Database (SGD)".

- Figure 3B: I suggest replacing 'blue filter' and 'red filter' with 'mTurquoise2' and 'mRuby2', respectively.

RESPONSE: Good point. Done.

Reviewer #3 (Significance (Required)):

Significance:
Cyclin-dependent kinases (CDKs) are important regulators of cell cycle progression. As such their activity is tightly regulated by several mechanisms including association with their respective cyclins, post-translational modifications, and inhibitory protein binding. In order to be active, canonical CDKs require phosphorylation of a threonine in their activation loop (T-loop) mediated by a cyclin-activating kinase (Cak1 in budding yeast, CDK7 in humans - though these two kinases differ significantly). Whereas T-loop phosphorylation and CAK activity have been considered to be continues for a long time (e.g. Kaldis et al., 1998; Tassan et al. 1994), CDK T-loop phosphorylations have more recently emerged as more regulated than initially presumed (e.g., Schachter et al. 2013, Mol Cell; Kirova et al. 2022 Dev Cell). Furthermore, CDK7 has recently emerged as an interesting target for cancer therapy being frequently overexpressed in cancer with several inhibitors in preclinical and clinical trials. Nevertheless, it has remained curious and unresolved, why CAK activity itself seems to be so little regulated.

RESPONSE: Thank you. We also added the following in the Discussion: "More generally, recent studies in mammals also suggest that Cdk T-loop phosphorylations are subject to greater regulatory control than previously assumed (Schachter et al, 2013; Kirova et al, 2022). In addition, human CAK has been identified as a promising target for cancer therapy, and several inhibitors are being evaluated in clinical trials (NCT04247126, NCT03134638, NCT05394103), underscoring the interest and significance of CAK-mediated control of cell proliferation."

General assessment:
The regulation of Cak1 by the uORF and the direct tuning of the expression levels by the growth rate via an uORF for a major cell cycle regulator is convincing and will be interesting to the (yeast) cell cycle community. However, the phenotypic analyses are technically less convincing, and I would encourage the authors to improve this part of the manuscript before publication. Furthermore, the manuscript can be strengthened by more clearly stating what has been done and improving how results are depicted.

Field of expertise: Cell cycle, CDK1 regulation, quantitative biology

Dear Dr. Polymenis,

Thank you for the submission of your revised manuscript. We have now received the enclosed reports from the referees and I am happy to say that all support its publication now. Referee 1 still has a few more suggestions that I would like you to incorporate before we can proceed with the official acceptance of your manuscript.

In case you do not agree with the data presentation issue raised by referee 1, can you please send me a point-by-point response that I can discuss with the other referees? Thank you.

A few editorial requests will also need to be addressed:

- Please write the abstract in present tense, as per journal policy.

- Please correct the content of the Data Availability Section to "This study includes no data deposited in external repositories".

- "Data not shown" is not allowed per journal policy. I think the statement in the methods can just be deleted, but please re-write the statement in the results section or show the data (page 14).

- Please co-submit with your final ms a completed author checklist, which you can download from our author guidelines <https://link.springer.com/journal/44319/submission-guidelines> The completed author checklist will also be part of the transparent peer-review file.

- The Reagents & Tools TABLE needs to be provided in the Word template we have in our guide to authors; it needs to be uploaded as Reagents table file type; the correct callout is Reagents and Tools Table (instead of Key Resources Table).
The Table template is available in our guide to authors online: https://link.springer.com/journal/44319/submission-guidelines

- Materials and Methods should be just Methods.

- BioRender should be acknowledged at the end of the Methods section in the following way: Graphics: (some of the... OR Figure #... OR synopsis) Graphics were created with BioRender.com.

- The resolution of the submitted figures is too low for blot and microscopy images. This reduction in resolution is commonly caused by converting original 16-bit TIFF files to RGB format for publication. While this is not inherently problematic, it can raise concerns about image integrity for critical readers.
To avoid any misunderstanding and to meet EMBO Press standards, we kindly ask that you: Resubmit the complete figure set at the captured original data resolution.

* Figure Legends - Comments *

- Please note that the exact p values are not provided in the legends of figure 6C. Please provide exact values as reasonable.
- Please note that information related to n is missing in the legends of figures 3C; EV-1A; EV-2A.

EMBO press papers are accompanied online by A) a short (1-2 sentences) summary of the findings and their significance, B) 2-3 bullet points highlighting key results and C) a synopsis image that is exactly 550 pixels wide and 300 pixels high. The synopsis image should provide a sketch of the major findings, like a graphical abstract. Please note that text needs to be readable at the final size. Please send us this information along with the final manuscript. (The current synopsis image needs to be removed from the ms file and uploaded as separate file. The current image size is not correct)

I look forward to seeing a final version of your manuscript as soon as possible.

Best regards,
Esther

Esther Schnapp, PhD
Senior Editor
EMBO reports

Referee #1:

In this revised submission, the authors have strengthened the manuscript in several ways including more convincing data on the viability differences of the uORF-0 and the uORF-16 strain during stationary phase, in competition assays, and the data on the increased T169 phosphorylation in the triple phosphatase knock-out. The authors have also added additional controls and experimental information, which has improved the clarity of the manuscript, and significantly expanded their discussion to put their findings into a larger context.

As discussed in my original review, the identification of translational regulation of Cak1 via an uORF is an interesting finding for the yeast cell cycle community and, more broadly, for the cell cycle field. Although the underlying mechanism and the phenotypic consequences remain incompletely defined in this manuscript and will require further investigation, the study nevertheless provides a sufficiently novel and important contribution to warrant publication.

Before publication, I have two mayor points and a few minor points, I would encourage the authors to consider.

Mayor:
- Reviewer #1 correctly pointed out that it is curious that in Figure 3C for Reporter 2 no reduction is observed in glycerol vs. glucose - something that had been observed for Cak1 in Figure 1. This puts into question the proposed mechanism by which the uORF is regulating translation of a downstream ORF and would suggest Cak1 specific elements to be contributing as well. However, neither in their rebuttal nor in their manuscript the authors seem to discuss these contradictory finding, but should do so.
- I had difficulties to follow how an increased T169 phosphorylation would cause a mitotic exit delay as the authors suggest. Maybe they would want to discuss this a bit more?

Minor:
- Whereas it is now much clearer to me, how the authors are normalizing many of their experimental data, I still find it not very intuitive and would encourage the authors to once more reconsider whether this is the most convincing way how to depict their data for the reader. A few examples:

(1) Because the normalization for V5-Cak1 used for Figure 1D includes data points that are not displayed in the figure (i.e., data presented later in Figure 2), the resulting transformation does not, in fact, produce the "symmetrical, biologically intuitive view of protein level fluctuations" around "a central baseline (zero)" claimed by the authors. Also, I can see how a central baseline makes sense, when normalizing longitudinal cell cycle data, but seems less desirable when comparing unperturbed to perturbed.
(2) For the normalization in 2B, the authors normalize the datapoints per target and immunoblot. However, not all immunoblots contain the same experimental conditions (two immunoblots do not have the uORF-3 glycerol condition). This could create a strange skew in the data: For example if uORF-3 glycerol had a dramatically increased Cak1 concentration compare to the other conditions, the baseline would be dramatically different for two of these experiments. I admit, this is not the case in this particular experiment, but highlights the general issue of applying such normalization on non-identical datasets.
(3) The fold-changes are hard to read and compare between conditions. The best example comes from the authors themselves, who write in the rebuttal: "Perhaps another way to look at the data is that V5-Cak1 levels are similar in all conditions in uORF-0 cells...". I would not call a consistent two-fold change between glucose and stationary similar. Furthermore, whereas the fold-change is clearly less pronounced the absolute decrease is actually similar (10000 to 5000 vs. 5000 to close to 0), something which is not at all obvious from the figure.
(4) In Figures 5C and EV5 the data is even derived from the same immunoblot. It is unclear why the data needed to undergo such normalization in the first place and just makes it more difficult to extract the information. In addition, the log2 with these axis-limits obscures any potential differences for Cdc28 and Pgk1.
- There are still several instances where the authors refer to 'independent experiments' without specifying how many experiments have been performed, sometimes one is able to guess from the figure, but sometimes one is not (e.g., how many replicates on the growth curves in 5C, are the averages or single data points?). I suggest to rigorously state the number of experiments and the applied statistics for each experiment and also highlight diversion (e.g. that in Figure 2 all experiment were performed four-times except the uORF-3 glycerol condition, which was only measured twice).
- I agree with reviewer #1 that the alternating left/right y-axis labels on many of the figures, make it more difficult to read the figures. I suggest to position them all on the left side.

Referee #2:

The authors responded to all points of my initial (lengthy) review. I see all critical points addressed: The figures are more clear, methods seem complete and transparent, important control/extension experiments have been done and successful, and the discussion now much better captures the context of previous findings in the field. I think this study is a meaningful addition to the cell cycle and growth control field and is suitable for publications in EMBO Reports.

Referee #3:

The authors have addressed my previous concerns. This work is a highly significant contribution to our understanding of cell cycle control mechanisms, and will be of considerable interest to members of the cell cycle and cancer biology fields.

We thank the reviewers for their time and effort. Our response to the remaining points from Reviewer 1 is shown below.
—---
Referee #1:

In this revised submission, the authors have strengthened the manuscript in several ways including more convincing data on the viability differences of the uORF-0 and the uORF-16 strain during stationary phase, in competition assays, and the data on the increased T169 phosphorylation in the triple phosphatase knock-out. The authors have also added additional controls and experimental information, which has improved the clarity of the manuscript, and significantly expanded their discussion to put their findings into a larger context.

As discussed in my original review, the identification of translational regulation of Cak1 via an uORF is an interesting finding for the yeast cell cycle community and, more broadly, for the cell cycle field. Although the underlying mechanism and the phenotypic consequences remain incompletely defined in this manuscript and will require further investigation, the study nevertheless provides a sufficiently novel and important contribution to warrant publication.

Before publication, I have two mayor points and a few minor points, I would encourage the authors to consider.

Mayor:
- Reviewer #1 correctly pointed out that it is curious that in Figure 3C for Reporter 2 no reduction is observed in glycerol vs. glucose - something that had been observed for Cak1 in Figure 1. This puts into question the proposed mechanism by which the uORF is regulating translation of a downstream ORF and would suggest Cak1 specific elements to be contributing as well. However, neither in their rebuttal nor in their manuscript the authors seem to discuss these contradictory finding, but should do so.

RESPONSE: We appreciate the reviewer pointing out this detail. We agree that in Figure 3C (third panel), mRuby2 expression reporting on Cak1 levels does not show a reduction in glycerol versus glucose.

However, we do not believe this observation challenges the proposed mechanism of uORF-mediated regulation. The lack of a glucose/glycerol difference in this specific figure is likely the result of using high-copy plasmids, which can saturate the regulatory machinery and mask physiological responses. Our proposed mechanism is fully supported by numerous experiments across various conditions using strains that express Cak1 from its endogenous chromosomal location (where native stoichiometry is maintained).

The primary purpose of the reporter plasmids in Figure 3C was to confirm two distinct points: 1) the uORF is indeed expressed in cells (first and second panels), and 2) removing the uORF significantly derepresses Cak1 synthesis (comparing the third and fourth panels). Notably, the degree of de-repression in the plasmid assay (10- to 15-fold)

is much greater than what we observe in the chromosomal strains, further supporting the idea that high-copy expression skews the absolute magnitude of regulation.

We agree that this discrepancy should be explicitly addressed in the text to avoid confusing the reader. We have modified the manuscript as follows:

"We note that the magnitude of the repression appears greater with these reporters compared to our results from immunoblotting (Figure 2B, 2- to 3-fold), and the regulatory difference between glucose and glycerol was blunted. This is likely an artifact of using high-copy plasmids in this specific assay."

- I had difficulties to follow how an increased T169 phosphorylation would cause a mitotic exit delay as the authors suggest. Maybe they would want to discuss this a bit more?

RESPONSE: We now added this statement in the text: "Although increased Cdk activity drives the G1/S transition, Cdk must be turned off for cells to complete mitosis. Hence, the elevated T-loop phosphorylation in these cells is expected to impede exit from mitosis."

Minor:
- Whereas it is now much clearer to me, how the authors are normalizing many of their experimental data, I still find it not very intuitive and would encourage the authors to once more reconsider whether this is the most convincing way how to depict their data for the reader. A few examples:

(1) Because the normalization for V5-Cak1 used for Figure 1D includes data points that are not displayed in the figure (i.e., data presented later in Figure 2), the resulting transformation does not, in fact, produce the "symmetrical, biologically intuitive view of protein level fluctuations" around "a central baseline (zero)" claimed by the authors. Also, I can see how a central baseline makes sense, when normalizing longitudinal cell cycle data, but seems less desirable when comparing unperturbed to perturbed.
(2) For the normalization in 2B, the authors normalize the datapoints per target and immunoblot. However, not all immunoblots contain the same experimental conditions (two immunoblots do not have the uORF-3 glycerol condition). This could create a strange skew in the data: For example if uORF-3 glycerol had a dramatically increased Cak1 concentration compare to the other conditions, the baseline would be dramatically different for two of these experiments. I admit, this is not the case in this particular experiment, but highlights the general issue of applying such normalization on non-identical datasets.

RESPONSE: To clarify, in all but two cases, we are <u>not</u> comparing the absolute baseline levels of wild-type cells versus uORF mutants. Instead, we are comparing the *relative* fluctuations of a single protein within *one specific strain* (e.g., wild-type or a specific mutant) across different growth conditions run on the same gel.

Because the internal mean normalization is calculated independently for each strain, the mutant data shown in Figure 2 does not mathematically influence or skew the wild-type normalization shown in Figure 1D. The "central baseline (zero)" represents the average expression of the protein for *that specific strain* across its tested conditions. This approach allows us to answer our primary biological question of how the abundance of this protein fluctuates internally when a given strain transitions from glucose, to glycerol, to stationary phase.

Similarly, regarding the missing *uORF-3* glycerol conditions in certain replicates (Point 2), because we are strictly analyzing the relative behavior of the protein within a given strain across available conditions, and not artificially anchoring mutant protein levels to a shared, absolute wild-type baseline, this does not create the cross-strain mathematical skew the reviewer is concerned about. The relative trends of repression and de-repression within the *uORF-3* strain remain robust and biologically consistent. We believe this internal normalization provides the most accurate reflection of the dynamic changes occurring within each specific genetic background.

There are only two cases (Figure 6C and EV5), where two different strains in one condition were compared and the levels normalized in this way. Although in both of these cases the samples were run on the same blot and the mean normalization is, in our opinion, still valid, we revised them with the normalization done against Pgk1 (as the reviewer suggested). There were no differences in the conclusions.

In all other cases, the comparisons are within each individual panel plot, not across all panels in a multipanel display. To make the above clearer, we added in the legend of Figure 1: "For each experiment, the signal for each quantified target was internally normalized by dividing its intensity by the average intensity for that target across all conditions *for that specific strain* on the same gel..."

(3) The fold-changes are hard to read and compare between conditions. The best example comes from the authors themselves, who write in the rebuttal: "Perhaps another way to look at the data is that V5-Cak1 levels are similar in all conditions in uORF-0 cells...". I would not call a consistent two-fold change between glucose and stationary similar. Furthermore, whereas the fold-change is clearly less pronounced the absolute decrease is actually similar (10000 to 5000 vs. 5000 to close to 0), something which is not at all obvious from the figure.

RESPONSE: We chose the Log2 method to visualize *relative fold changes*, since immunoblot signals do not provide absolute counts. This approach also captures proportional shifts in protein levels that typically drive many biological transitions and threshold responses. The Log2 transformation yields a straightforward, easy to read display, highlights relative differences by design, and it is in widespread use in most gene expression studies. We hope this clarifies our rationale for maintaining the Log2 scale for fold-change differences in protein levels.

(4) In Figures 5C and EV5 the data is even derived from the same immunoblot. It is unclear why the data needed to undergo such normalization in the first place and just makes it more difficult to extract the information. In addition, the log2 with these axis-limits obscures any potential differences for Cdc28 and Pgk1.

RESPONSE: The reviewer probably refers to Fig 6C (Fig 5C has growth curves). As we mentioned above, we revised the normalization of that figure. As for EV5, where uORF-16 and uORF-35 cells are compared, we also revised the normalization as suggested, again showing no difference.

- There are still several instances where the authors refer to 'independent experiments' without specifying how many experiments have been performed, sometimes one is able to guess from the figure, but sometimes one is not (e.g., how many replicates on the growth curves in 5C, are the averages or single data points?). I suggest to rigorously state the number of experiments and the applied statistics for each experiment and also highlight diversion (e.g. that in Figure 2 all experiment were performed four-times except the uORF-3 glycerol condition, which was only measured twice).

RESPONSE: Thank you, indeed the only exception in Figure 2 was the uORF-3 glycerol condition. We now state that in the figure legend. We also added in the Methods that "Every datapoint shown is from a separate experiment." As for Figure 5C, the growth curves, it clearly shows three separate experiments for each strain. We now added that information in the legend.

- I agree with reviewer #1 that the alternating left/right y-axis labels on many of the figures, make it more difficult to read the figures. I suggest to position them all on the left side.

RESPONSE: While we appreciate the suggestion, we believe the current compact layout is the most efficient and visually pleasing way for readers to compare the data across groups, and we have decided to keep the original formatting. The alternating y-axis labels are a core design principle of the Trellis graphics framework (implemented via the R lattice package). This layout maximizes the data-ink ratio by allowing adjacent panels to share boundaries tightly.

Michael Polymenis
Texas A&M University
Biochemistry and Biophysics
2128 TAMU
College Station, Texas 77843
United States

Dear Michael,

I am very pleased to accept your manuscript for publication in the next available issue of EMBO reports. Thank you for your contribution to our journal.

Your manuscript will be processed for publication by EMBO Press. It will be copy edited and you will receive page proofs prior to publication. Please note that you will be contacted by Springer Nature Author Services to complete licensing and payment information.

You may qualify for financial assistance for your publication charges - either via a Springer Nature fully open access agreement or an EMBO initiative. Check your eligibility: https://link.springer.com/journal/44319/how-to-publish-with-us

Should you be planning a Press Release on your article, please notify the production team via this form in order to coordinate publication and release dates: https://forms.cloud.microsoft/e/eH4A8JVP6g

If you have any questions, please do not hesitate to contact the Editorial Office. Thank you for your contribution to EMBO Reports.

Best regards,
Esther

Esther Schnapp, PhD
Senior Editor
EMBO reports

>>> Please note that it is EMBO Reports policy for the transcript of the editorial process (containing referee reports and your response letter) to be published as an online supplement to each paper. If you do NOT want this, you will need to inform the Editorial Office via email immediately. More information is available here: https://link.springer.com/partners/embo-press/editorial-policies#Peer%20review

EMBO Press Author Checklist

Corresponding Author Name: Michael Polymenis	
Journal Submitted to: EMBO Reports	
Manuscript Number: EMBOR-2026-64565	

USEFUL LINKS FOR COMPLETING THIS FORM
The EMBO Journal - Author Guidelines
EMBO Reports - Author Guidelines
Molecular Systems Biology - Author Guidelines
EMBO Molecular Medicine - Author Guidelines

Reporting Checklist for Life Science Articles (updated January
This checklist is adapted from Materials Design Analysis Reporting (MDAR) Checklist for Authors. MDAR establishes a minimum set of requirements in transparent reporting in the life sciences (see Statement of Task: 10.31222/osf.io/9sm4x). Please follow the journal's guidelines in preparing your manuscript.
Please note that a copy of this checklist will be published alongside your article.

Abridged guidelines for figures
1. Data
The data shown in figures should satisfy the following conditions:
- the data were obtained and processed according to the field's best practice and are presented to reflect the results of the experiments in an accurate and unbiased manner.
- ideally, figure panels should include only measurements that are directly comparable to each other and obtained with the same assay.
- plots include clearly labeled error bars for independent experiments and sample sizes. Unless justified, error bars should not be shown for technical
- if n<5, the individual data points from each experiment should be plotted. Any statistical test employed should be justified.
- Source Data should be included to report the data underlying figures according to the guidelines set out in the authorship guidelines on Data

2. Captions
Each figure caption should contain the following information, for each panel where they are relevant:
- a specification of the experimental system investigated (eg cell line, species name).
- the assay(s) and method(s) used to carry out the reported observations and measurements.
- an explicit mention of the biological and chemical entity(ies) that are being measured.
- an explicit mention of the biological and chemical entity(ies) that are altered/varied/perturbed in a controlled manner.
- the exact sample size (n) for each experimental group/condition, given as a number, not a range;
- a description of the sample collection allowing the reader to understand whether the samples represent technical or biological replicates (including how many animals, litters, cultures, etc.).
- a statement of how many times the experiment shown was independently replicated in the laboratory.
- definitions of statistical methods and measures:

- common tests, such as t-test (please specify whether paired vs. unpaired), simple χ2 tests, Wilcoxon and Mann-Whitney tests, can be unambiguously identified by name only, but more complex techniques should be described in the methods section;

- are tests one-sided or two-sided?
- are there adjustments for multiple comparisons?
- exact statistical test results, e.g., P values = x but not P values < x;
- definition of 'center values' as median or average;
- definition of error bars as s.d. or s.e.m.

Please complete ALL of the questions below.
Select "Not Applicable" only when the requested information is not relevant for your study.

Materials

Newly Created Materials	Information included in the manuscript?	In which section is the information available? (Reagents and Tools Table, Materials and Methods, Figures, Data Availability Section)
New materials and reagents need to be available; do any restrictions apply?	Yes	Data Availability Section

Antibodies	Information included in the manuscript?	In which section is the information available? (Reagents and Tools Table, Materials and Methods, Figures, Data Availability Section)
For **antibodies** provide the following information:<br>- Commercial antibodies: RRID (if possible) or supplier name, catalogue number and or/clone number<br>- Non-commercial: RRID or citation	Yes	Reagents and Tools Table

DNA and RNA sequences	Information included in the manuscript?	In which section is the information available? (Reagents and Tools Table, Materials and Methods, Figures, Data Availability Section)
**Short novel DNA or RNA including primers, probes**: provide the sequences.	Yes	Reagents and Tools Table

Cell materials	Information included in the manuscript?	In which section is the information available? (Reagents and Tools Table, Materials and Methods, Figures, Data Availability Section)
**Cell lines:** Provide species information, strain. Provide accession number in repository **OR** supplier name, catalog number, clone number, and/**OR** RRID.	Not Applicable	
**Primary cultures:** Provide species, strain, sex of origin, genetic modification status.	Not Applicable	
Report if the cell lines were recently **authenticated** (e.g., by STR profiling) and tested for mycoplasma contamination.	Not Applicable	

Experimental animals	Information included in the manuscript?	In which section is the information available? (Reagents and Tools Table, Materials and Methods, Figures, Data Availability Section)
**Laboratory animals or Model organisms:** Provide species, strain, sex, age, genetic modification status. Provide accession number in repository **OR** supplier name, catalog number, clone number, **OR** RRID.	Not Applicable	
**Animal observed in or captured from the field:** Provide species, sex, and age where possible.	Not Applicable	
Please detail **housing and husbandry conditions**.	Not Applicable	

Plants and microbes	Information included in the manuscript?	In which section is the information available? (Reagents and Tools Table, Materials and Methods, Figures, Data Availability Section)
**Plants:** provide species and strain, ecotype and cultivar where relevant, unique accession number if available, and source (including location for collected wild specimens).	Not Applicable	
**Microbes:** provide species and strain, unique accession number if available, and source.	Yes	Reagents and Tools Table, Methods

Human research participants	Information included in the manuscript?	In which section is the information available? (Reagents and Tools Table, Materials and Methods, Figures, Data Availability Section)
If collected and within the bounds of privacy constraints report on age, sex and gender or ethnicity for all study participants.	Not Applicable	

Core facilities	Information included in the manuscript?	In which section is the information available? (Reagents and Tools Table, Materials and Methods, Figures, Data Availability Section)
If your work benefited from core facilities, was their service mentioned in the acknowledgments section?	Yes	Acknowledgements

Design

Study protocol	Information included in the manuscript?	In which section is the information available? (Reagents and Tools Table, Materials and Methods, Figures, Data Availability Section)
If study protocol has been **pre-registered, provide DOI in the manuscript**. For clinical trials, provide the trial registration number **OR** cite DOI.	Not Applicable	
Report the **clinical trial registration number** (at ClinicalTrials.gov or equivalent), where applicable.	Not Applicable	

Laboratory protocol	Information included in the manuscript?	In which section is the information available? (Reagents and Tools Table, Materials and Methods, Figures, Data Availability Section)
Provide DOI OR other citation details if **external detailed step-by-step protocols** are available.	Not Applicable	

Experimental study design and statistics	Information included in the manuscript?	In which section is the information available? (Reagents and Tools Table, Materials and Methods, Figures, Data Availability Section)
Include a statement about **sample size** estimate even if no statistical methods were used.	Yes	Methods
Were any steps taken to minimize the effects of subjective bias when allocating animals/samples to treatment (e.g. **randomization procedure**)? If yes, have they been described?	Yes	Methods
Include a statement about **blinding** even if no blinding was done.	Yes	Methods
Describe **inclusion/exclusion criteria** if samples or animals were excluded from the analysis. Were the criteria pre-established?<br><br>If sample or data points were omitted from analysis, report if this was due to attrition or intentional exclusion and provide justification.	Yes	Methods
For every figure, are **statistical tests** justified as appropriate? Do the data meet the assumptions of the tests (e.g., normal distribution)? Describe any methods used to assess it. Is there an estimate of variation within each group of data? Is the variance similar between the groups that are being statistically compared?	Yes	Methods

Sample definition and in-laboratory replication	Information included in the manuscript?	In which section is the information available? (Reagents and Tools Table, Materials and Methods, Figures, Data Availability Section)
In the figure legends: state number of times the experiment was **replicated** in laboratory.	Yes	Methods, Figures
In the figure legends: define whether data describe **technical or biological replicates**.	Yes	Methods, Figures

Ethics

Ethics	Information included in the manuscript?	In which section is the information available? (Reagents and Tools Table, Materials and Methods, Figures, Data Availability Section)
Studies involving **human participants**: State details of **authority granting ethics approval** (IRB or equivalent committee(s), provide reference number for approval.	Not Applicable	
Studies involving **human participants**: Include a statement confirming that **informed consent** was obtained from all subjects and that the experiments conformed to the principles set out in the WMA Declaration of Helsinki and the Department of Health and Human Services Belmont Report.	Not Applicable	
Studies involving **human participants:** For publication of **patient photos**, include a statement confirming that consent to publish was obtained.	Not Applicable	
Studies involving experimental **animals**: State details of **authority granting ethics approva**l (IRB or equivalent committee(s), provide reference number for approval. Include a statement of compliance with ethical regulations.	Not Applicable	
Studies involving **specimen and field samples:** State if relevant **permits** obtained, provide details of authority approving study; if none were required, explain why.	Not Applicable	

Dual Use Research of Concern (DURC)	Information included in the manuscript?	In which section is the information available? (Reagents and Tools Table, Materials and Methods, Figures, Data Availability Section)
Could your study fall under dual use research restrictions? Please check biosecurity documents and list of **select agents and toxins** (CDC): https://www.selectagents.gov/sat/list.htm	Not Applicable	
If you used a select agent, is the security level of the lab appropriate and reported in the manuscript?	Not Applicable	
If a study is subject to dual use research of concern regulations, is the name of the **authority granting approval and reference number** for the regulatory approval provided in the manuscript?	Not Applicable	

Reporting

The MDAR framework recommends adoption of discipline-specific guidelines, established and endorsed through community initiatives. Journals have their own policy about requiring specific guidelines and recommendations to complement MDAR.

Adherence to community standards	Information included in the manuscript?	In which section is the information available? (Reagents and Tools Table, Materials and Methods, Figures, Data Availability Section)
State if relevant guidelines or checklists (e.g., **ICMJE, MIBBI, ARRIVE, PRISMA**) have been followed or provided.	Not Applicable	
For **tumor marker prognostic studies,** we recommend that you follow the **REMARK** reporting guidelines (see link list at top right). See author guidelines, under 'Reporting Guidelines'. Please confirm you have followed these guidelines.	Not Applicable	
For **phase II and III randomized controlled trials**, please refer to the **CONSORT** flow diagram (see link list at top right) and submit the CONSORT checklist (see link list at top right) with your submission. See author guidelines, under 'Reporting Guidelines'. Please confirm you have submitted this list.	Not Applicable	

Data Availability

Data availability	Information included in the manuscript?	In which section is the information available? (Reagents and Tools Table, Materials and Methods, Figures, Data Availability Section)
Have **primary datasets** been deposited according to the journal's guidelines (see 'Data Deposition' section) and the respective accession numbers provided in the Data Availability Section?	Not Applicable	
Were **human clinical and genomic datasets** deposited in a public access-controlled repository in accordance to ethical obligations to the patients and to the applicable consent agreement?	Not Applicable	
Are **computational models** that are central and integral to a study available without restrictions in a machine-readable form? Were the relevant accession numbers or links provided?	Not Applicable	
If publicly available data were reused, provide the respective **data citations in the reference list**.	Not Applicable	
