# Supplementary material for: uORF-mediated translational control of CAK and Cdk activation in response to low nutrients in yeast
Source: EMBO Rep. 2026 Sep 5;27(19):5582–605. doi: 10.1038/s44319-026-00916-z (PMC13649776; doi:10.1038/s44319-026-00916-z)
Supplement: Supplementary file 5 [file 44319_2026_916_MOESM12_ESM.zip › FIGURE EV5/FigureEV5_RawBlots.pdf]

FigureEV5_RawBlots. Immunoblots used in the quantification of the indicated targets in Figure EV5.
The images to the right are cropped and background subtracted and were used for quantification.

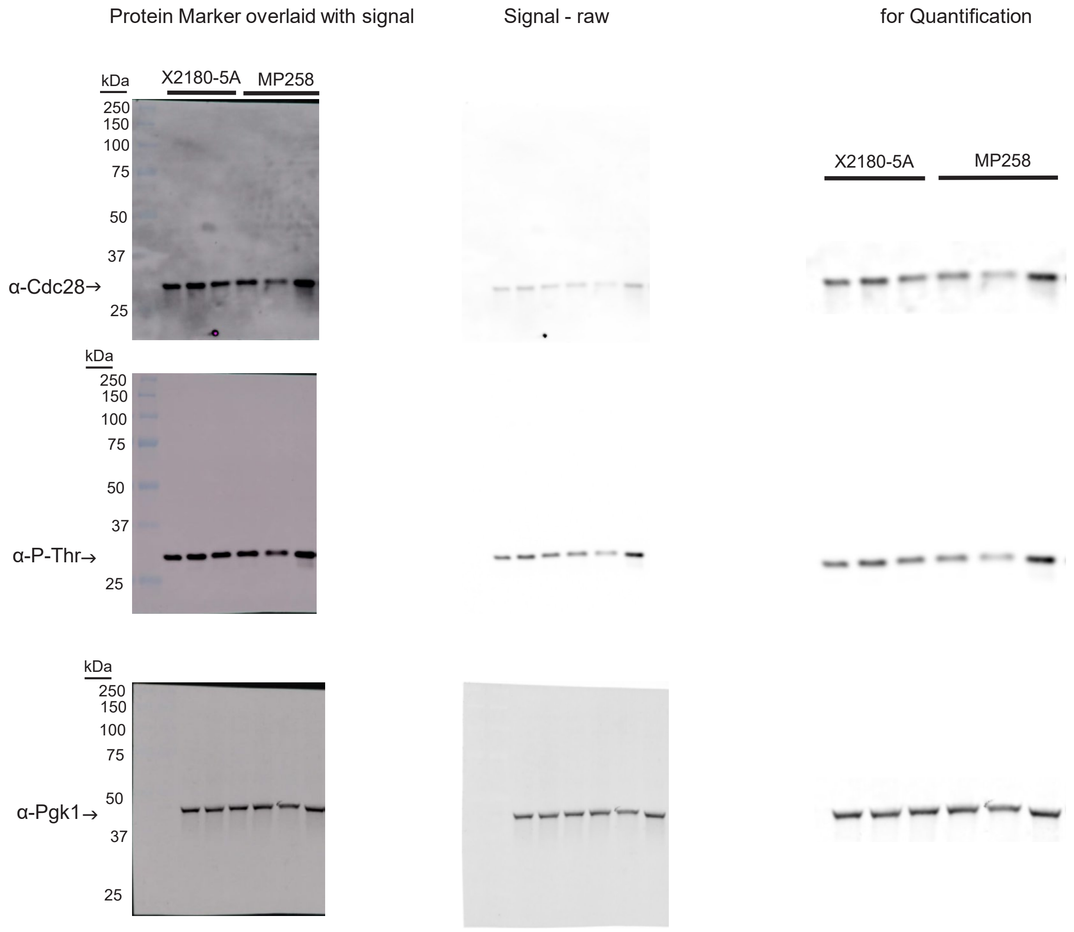

Samples from YPD cultures second set 5/30/26, harvested early log phase
NaOH protein prep from samples frozen in -80°C. Bio-Rad pre-cast gradient gel.
Primary antibodies:
- Pgk1 (1:4000) Invitrogen 22CSD8. (**Fix--this is the new Abcam AB, I think.)
- P-Thr Cdc28 (custom 1:2000)
- Cdc28 (G-7) Sc-515762 (1:1,500)

Secondary antibodies:
- goat α-mouse HRP (1:5,000) for Cdc28 total
- goat α-mouse Alexa 488 (1:5,000) for Pgk1
- goat α-rabbit HRP (1:5,000)

Images quantified and background subtracted using ImageJ software.
