# Supplementary material for: uORF-mediated translational control of CAK and Cdk activation in response to low nutrients in yeast
Source: EMBO Rep. 2026 Sep 5;27(19):5582–605. doi: 10.1038/s44319-026-00916-z (PMC13649776; doi:10.1038/s44319-026-00916-z)

Strain MP258 (uORF-35): 2/24/2024 Glucose limited chemostat

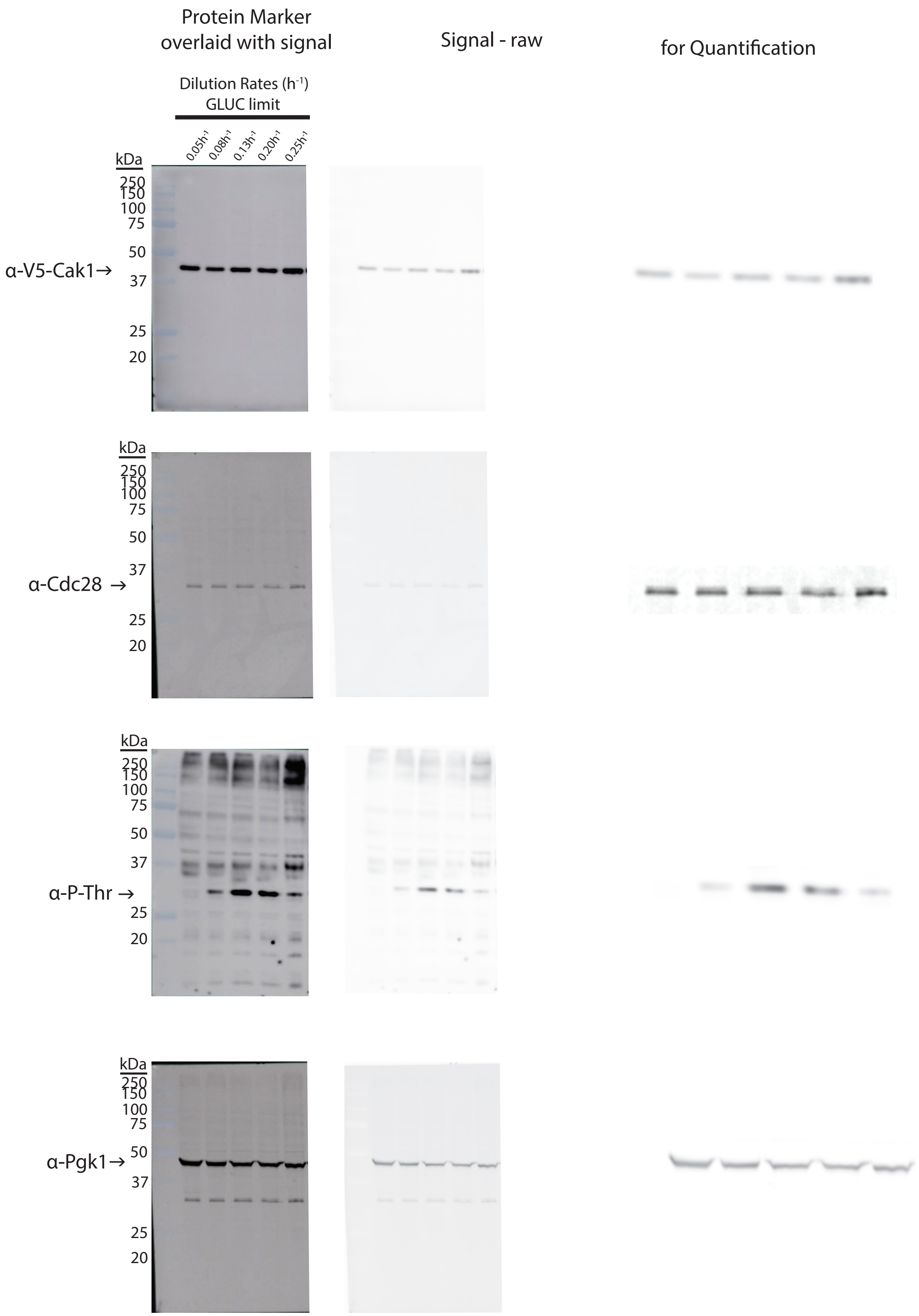

Strain MP258 (uORF-35): 4/15/2024 Glucose limited chemostat

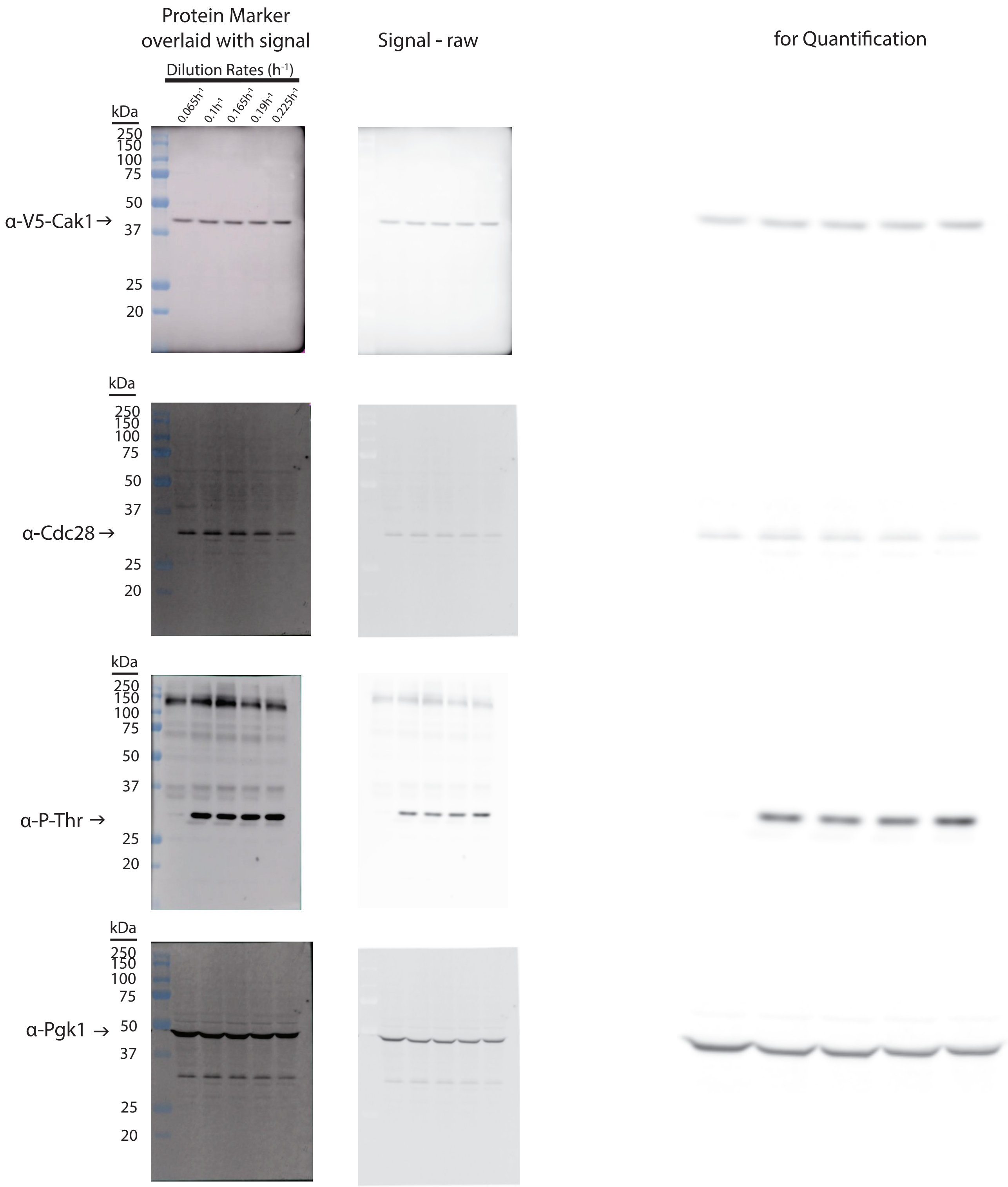

Strain MP258 (uORF-35): 5/17/2024 Glucose limited chemostat

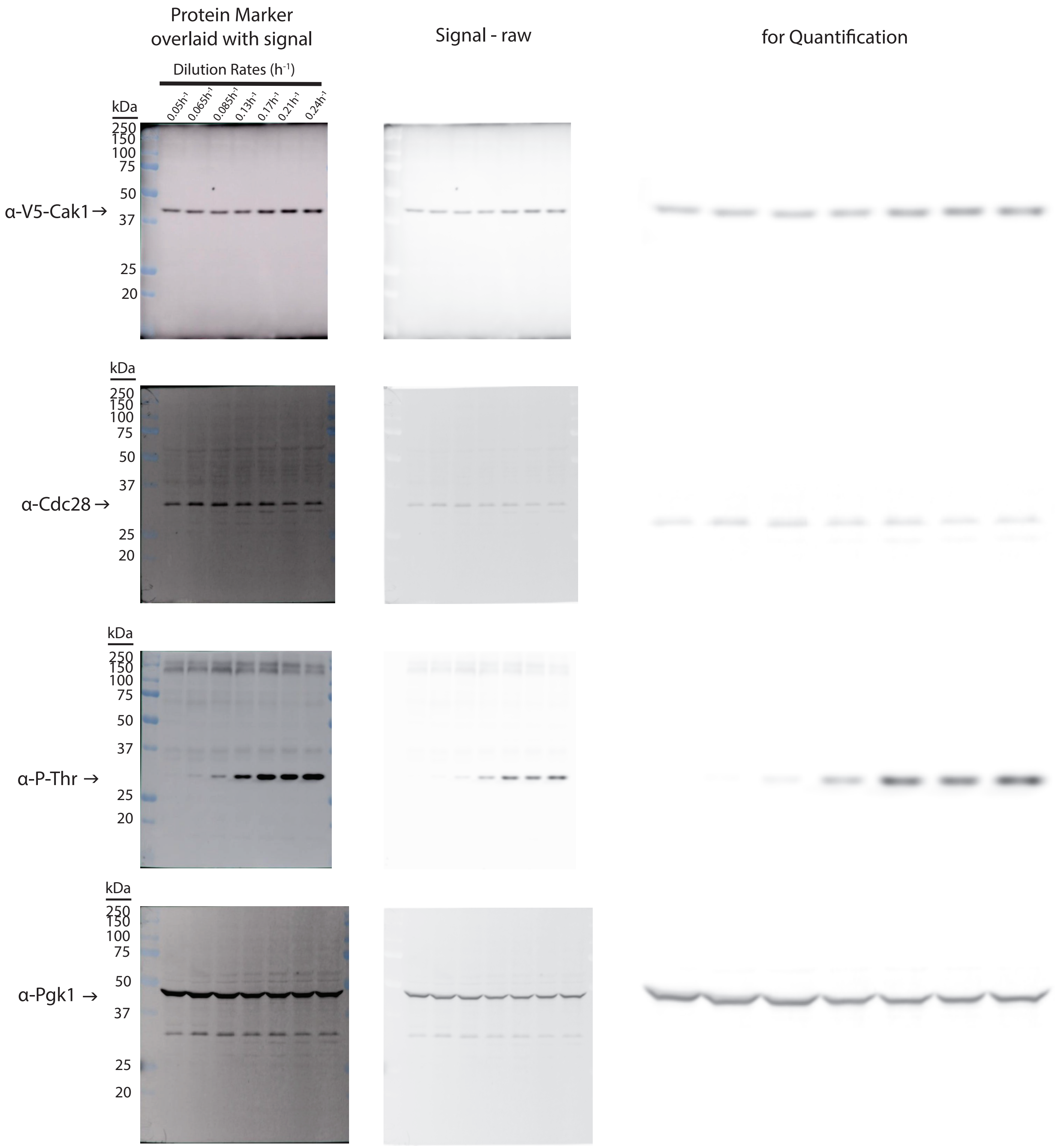

Strain MP258 (uORF-35): 3/1/2024 Nitrogen limited chemostat

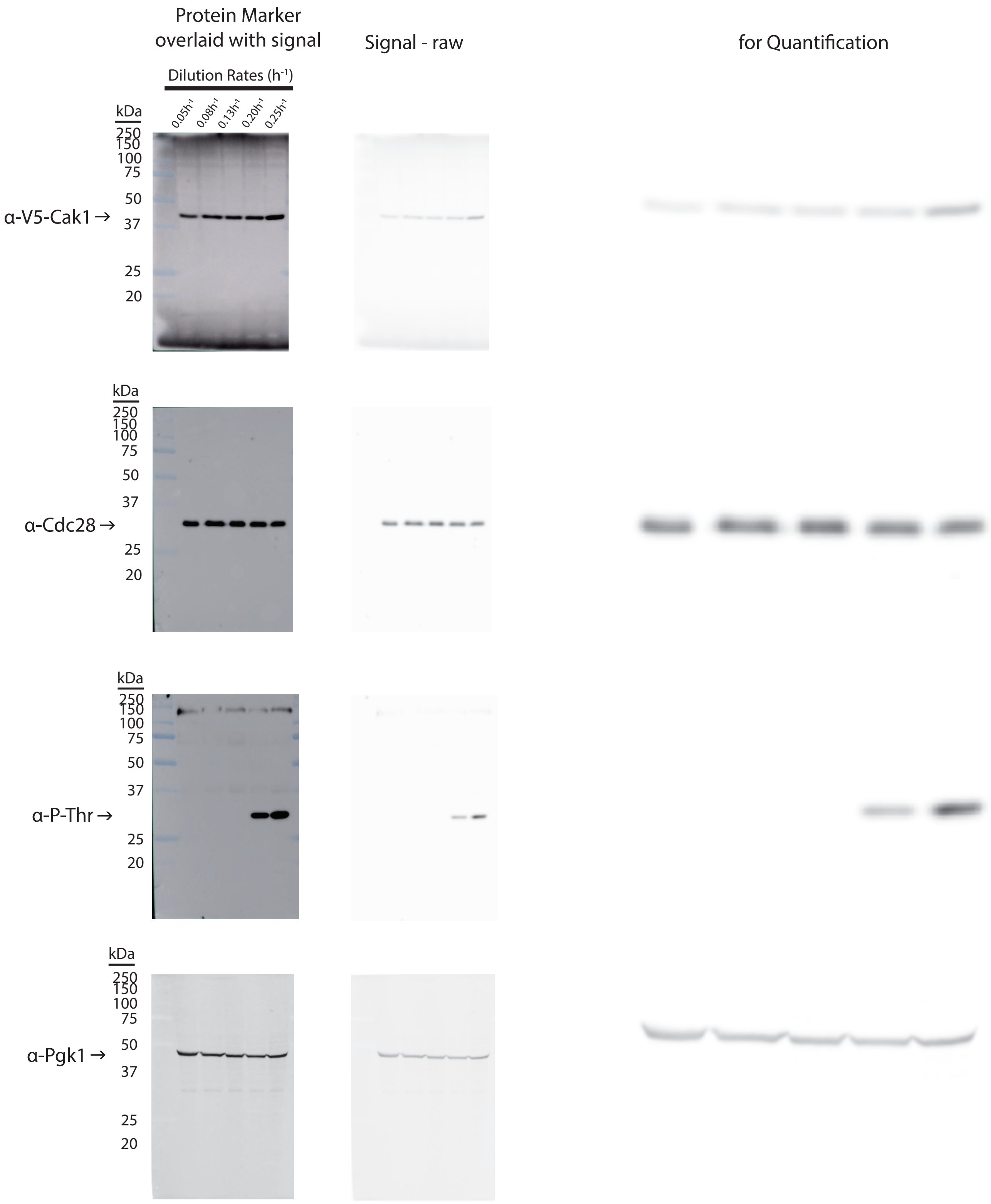

Strain MP258 (uORF-35): 4/1/2024 Nitrogen limited chemostat

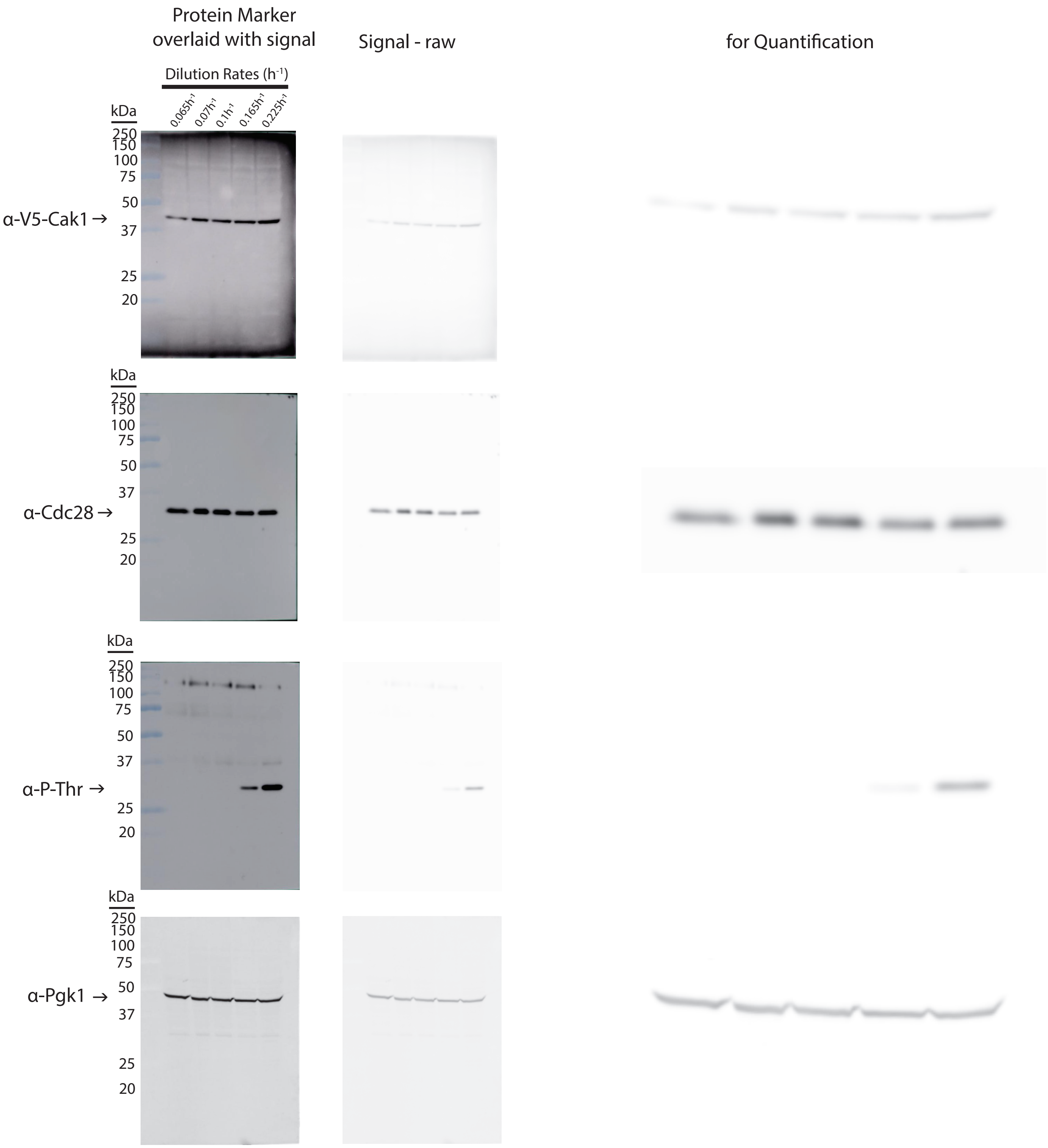

MP258 (uORF-35): 5/31/2024 Nitrogen limited chemostat

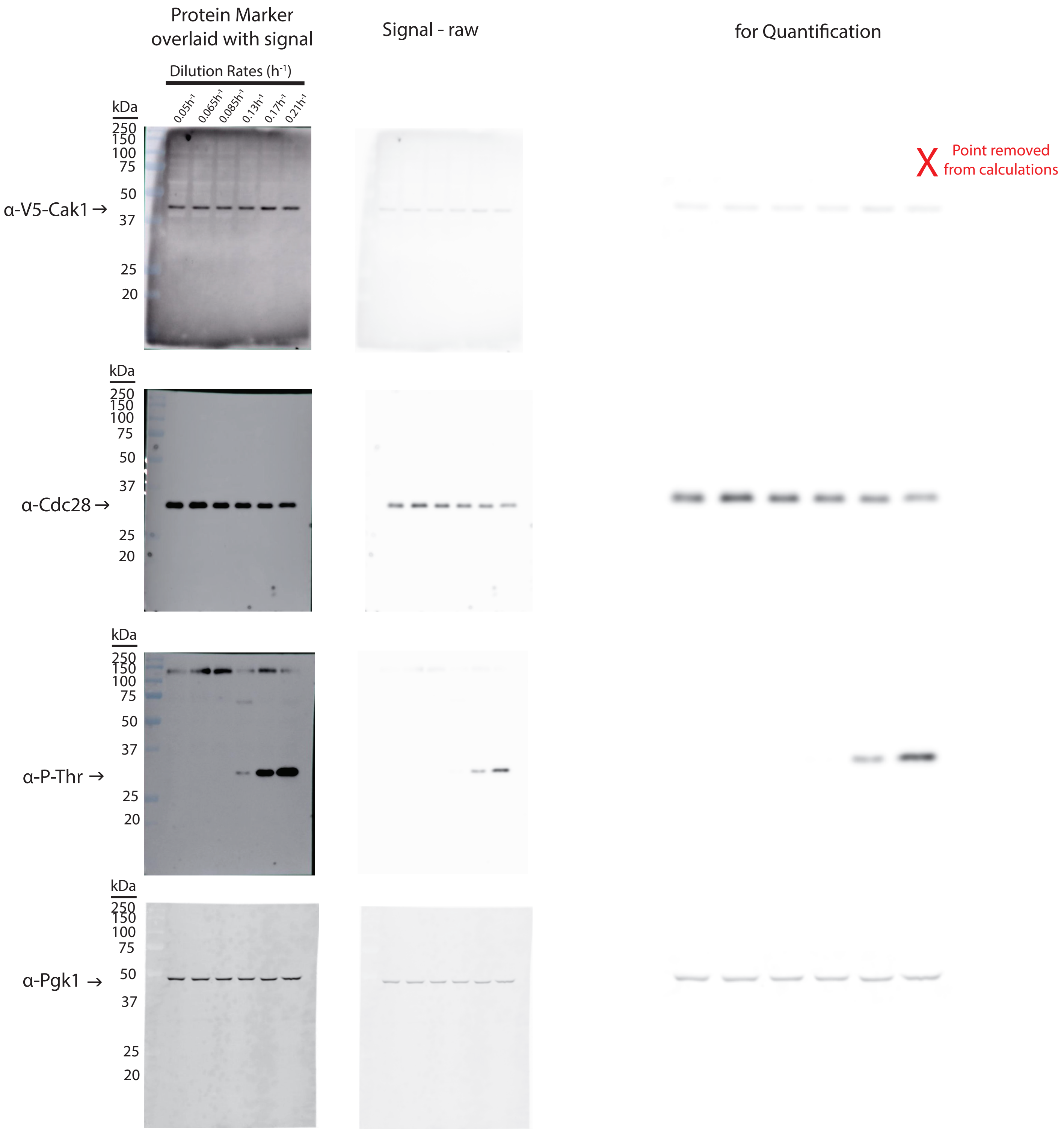

Strain MP259 (uORF-0): 1-16-2025 Glucose limited chemostat

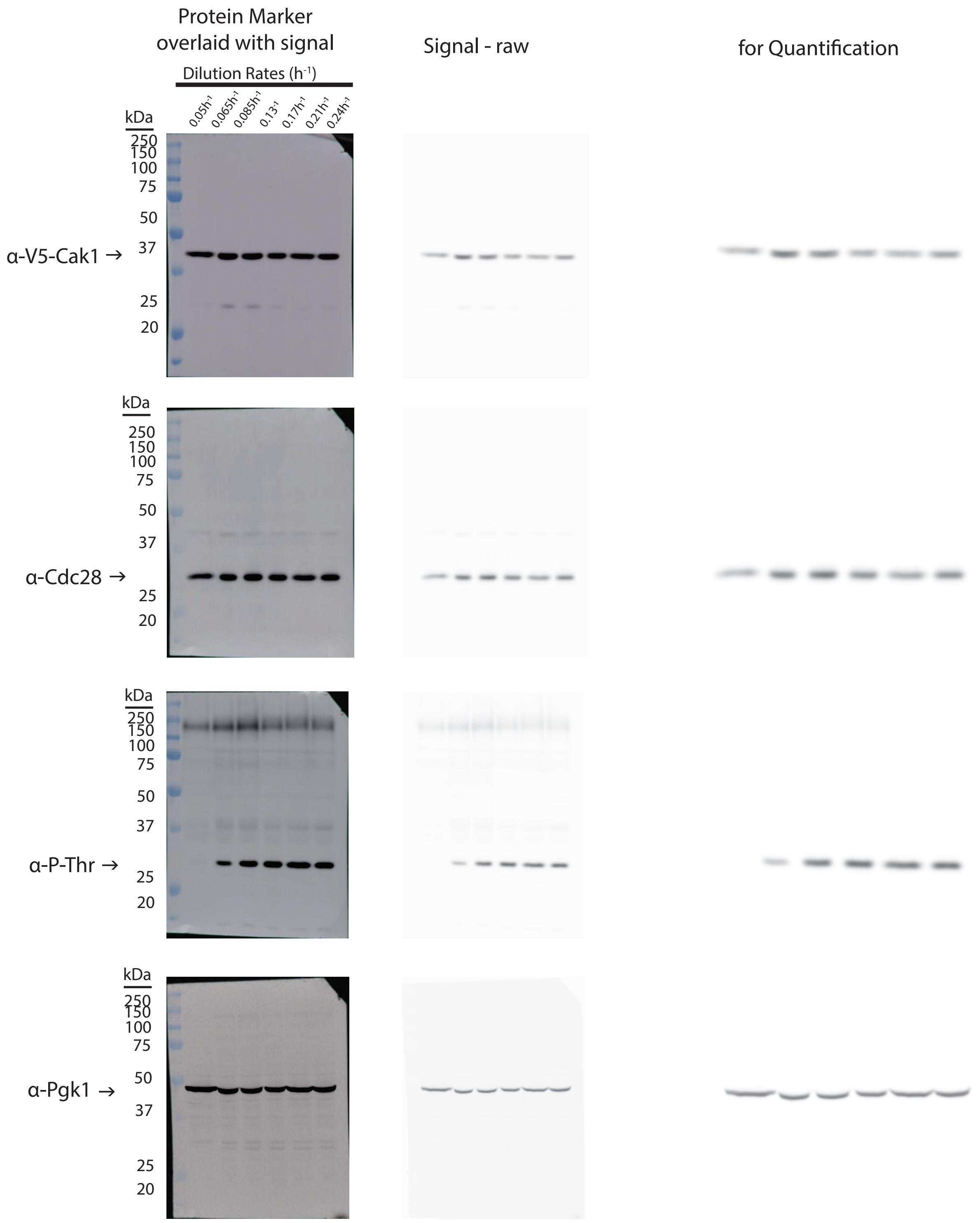

Strain MP259 (uORF-0): 10/2/2024 Glucose limited chemostat

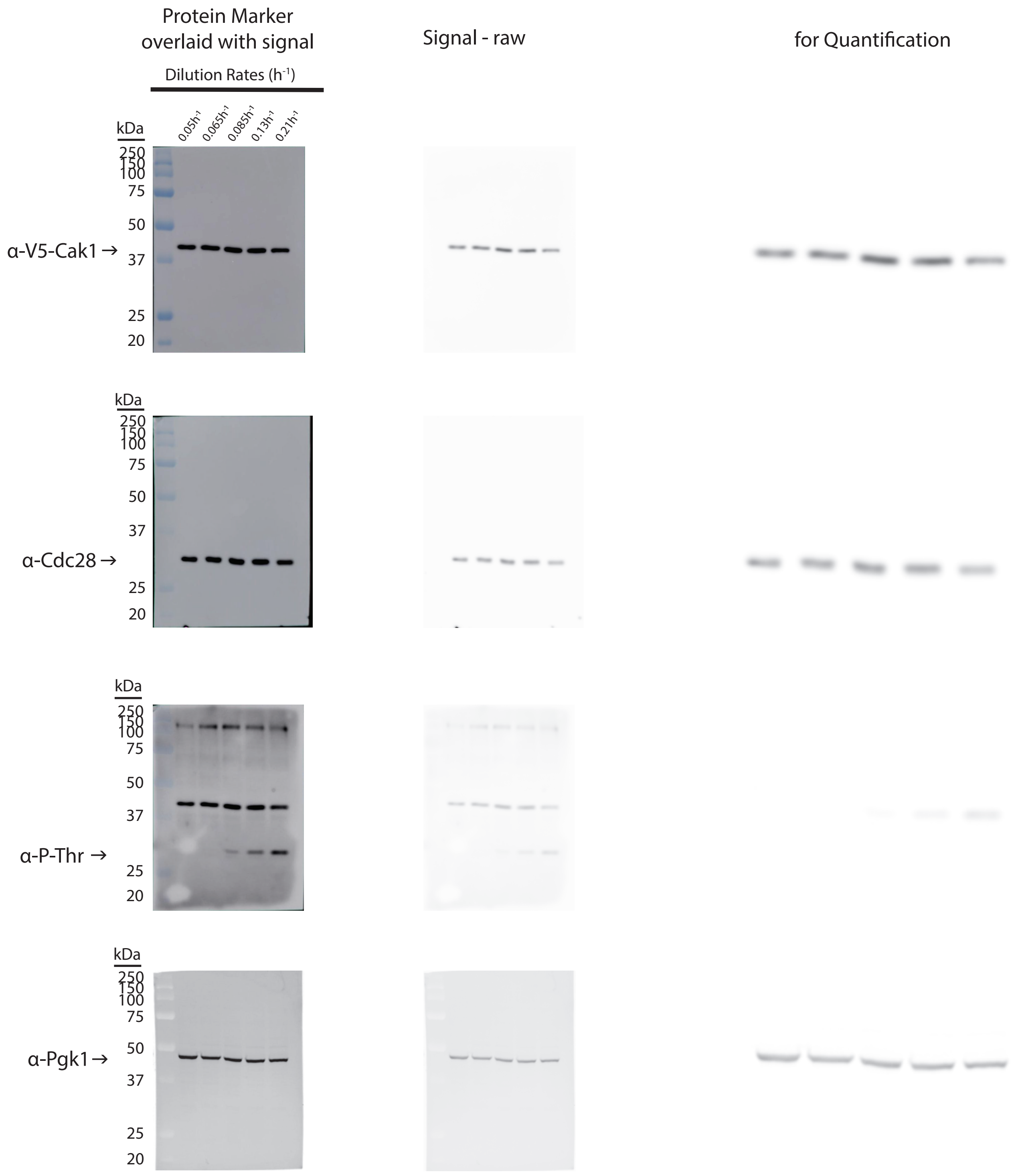

Strain MP259 (uORF-0): 12/4/2024 Glucose limited chemostat

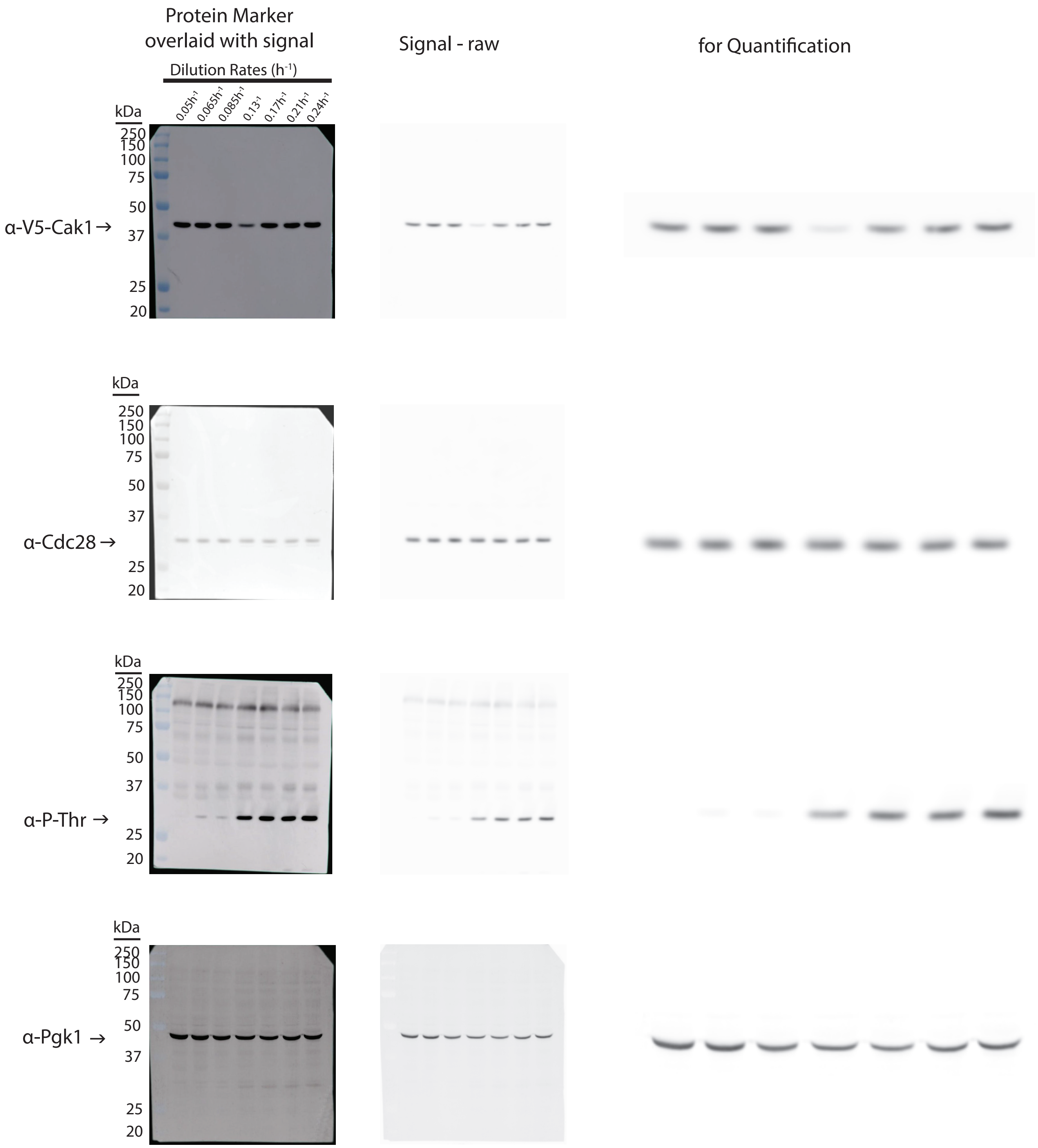

Strain MP259 (uORF-0): 2/19/2025 Nitrogen limited chemostat

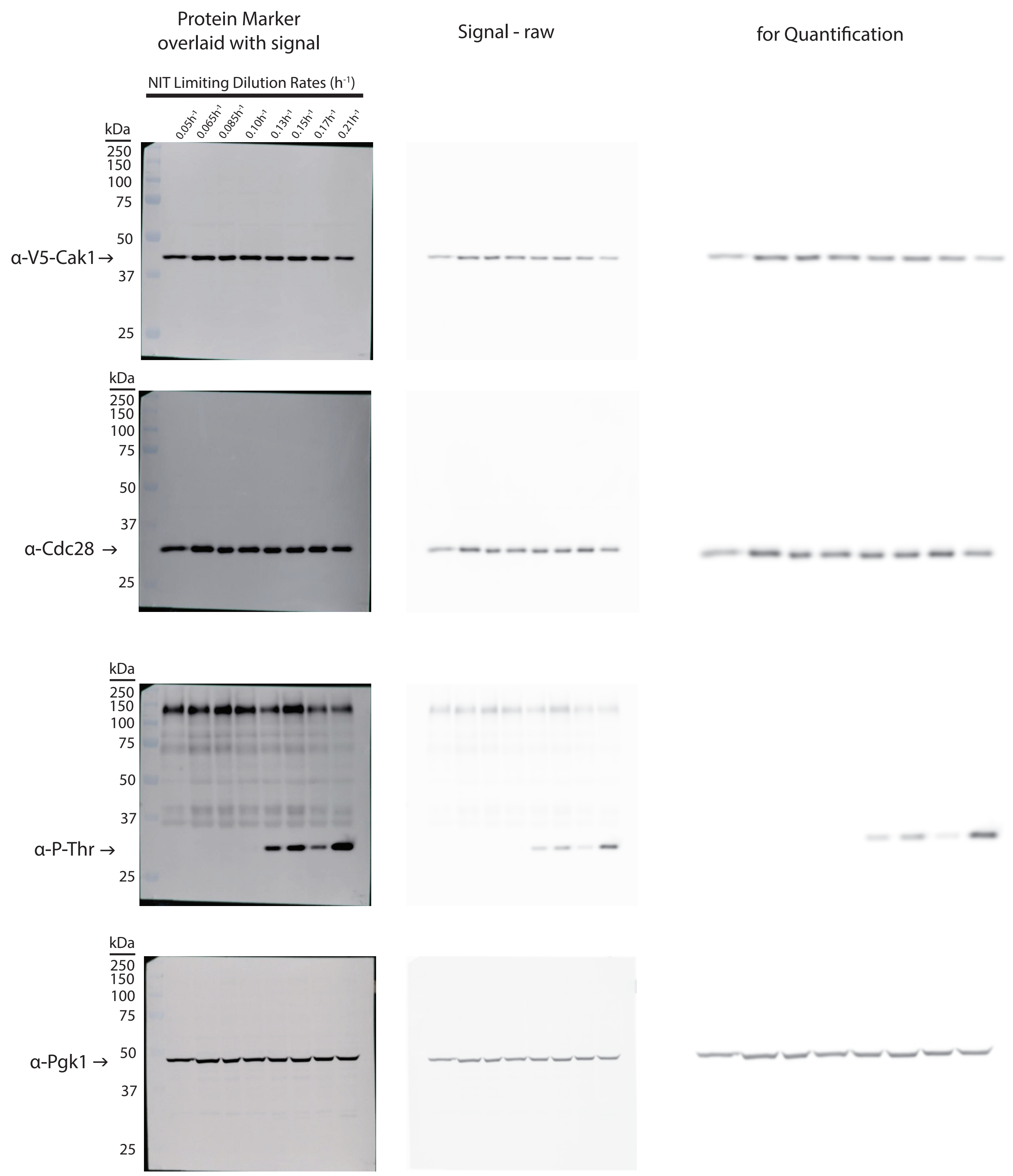

Strain MP259 (uORF-0): 7/20/2025 Nitrogen limited chemostat

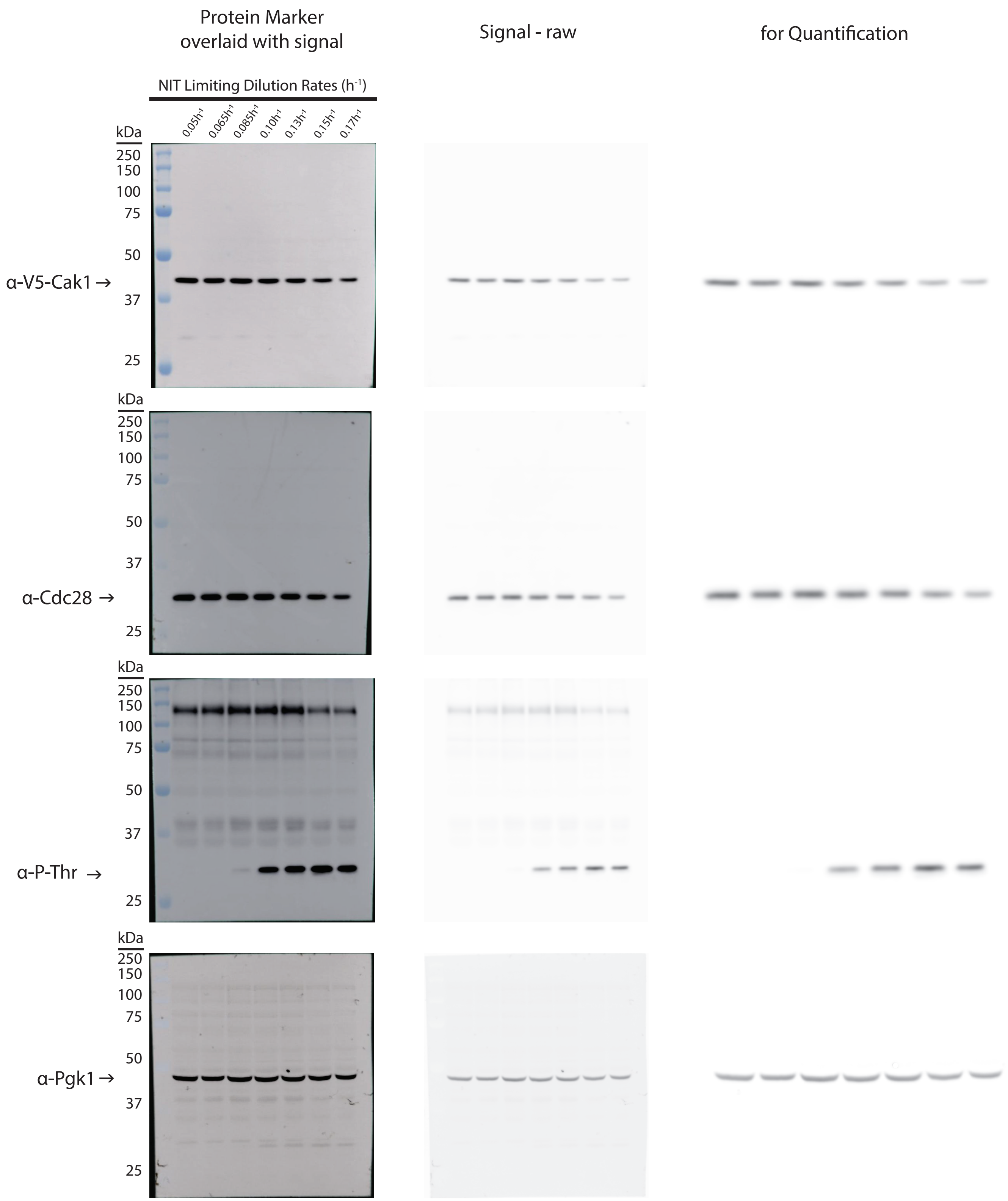

Supplement: Supplementary file 9 [file 44319_2026_916_MOESM5_ESM.zip › FIGURE 4/Figure4_RawBlots.pdf]
