# Supplementary material for: uORF-mediated translational control of CAK and Cdk activation in response to low nutrients in yeast
Source: EMBO Rep. 2026 Sep 5;27(19):5582–605. doi: 10.1038/s44319-026-00916-z (PMC13649776; doi:10.1038/s44319-026-00916-z)

Figure5F_RawBlots. Immunoblots used in the quantification of the indicated targets in Figure 5F.

Experiment 1 (20260414):

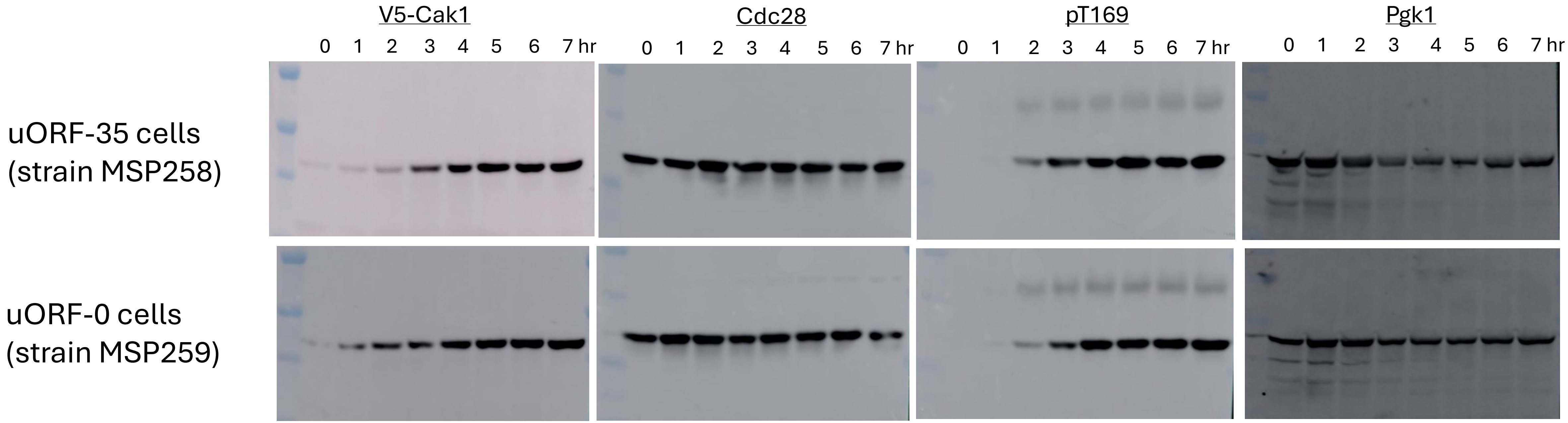

Figure5F_RawBlots. Immunoblots used in the quantification of the indicated targets in Figure 5F.

Experiment 2 (20260420):

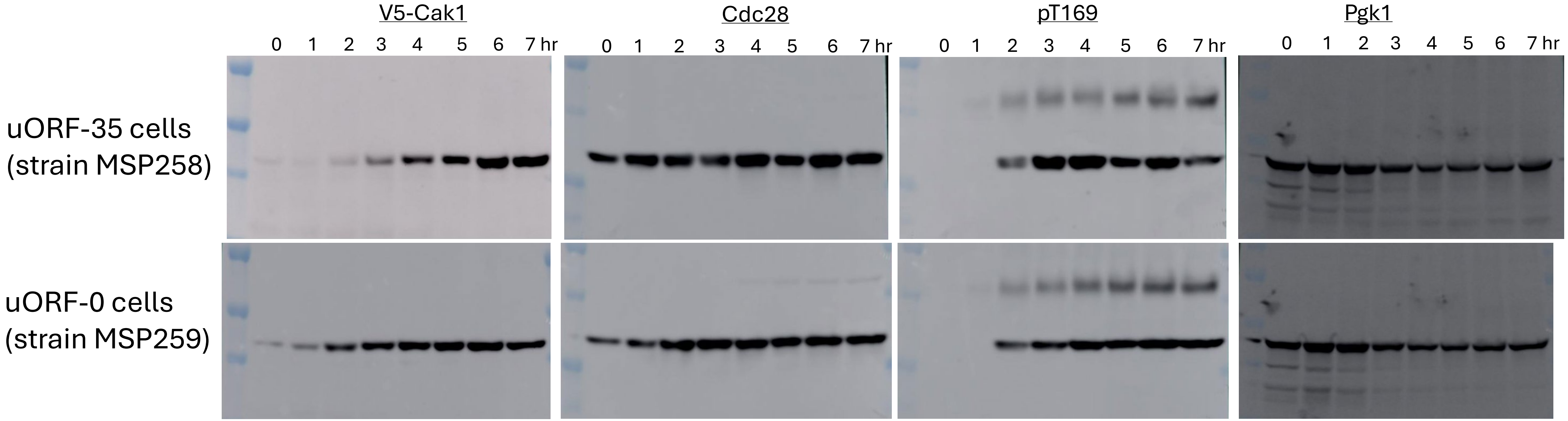

Figure5F_RawBlots. Immunoblots used in the quantification of the indicated targets in Figure 5F.

Experiment 3 (20260504):

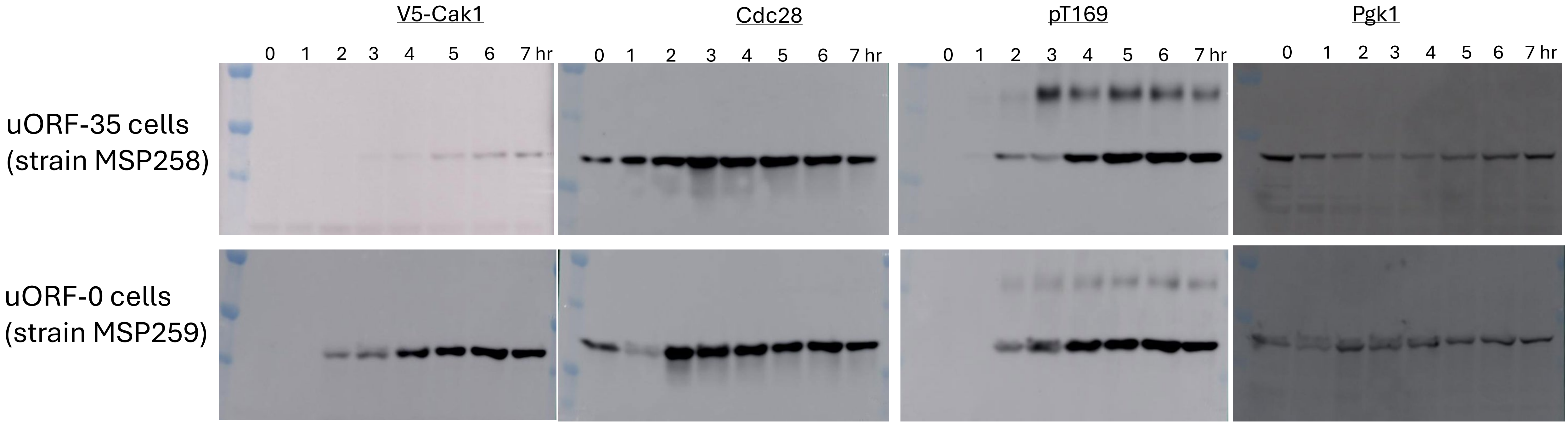

Supplement: Supplementary file 10 [file 44319_2026_916_MOESM6_ESM.zip › FIGURE 5/Figure5F_RawBlots.pdf]
