# Supplementary material for: uORF-mediated translational control of CAK and Cdk activation in response to low nutrients in yeast
Source: EMBO Rep. 2026 Sep 5;27(19):5582–605. doi: 10.1038/s44319-026-00916-z (PMC13649776; doi:10.1038/s44319-026-00916-z)

Figure6_RawBlots. Immunoblots used in the quantification of the indicated targets in Figure 6C. The images to the right are cropped and background subtracted and were used for quantification.

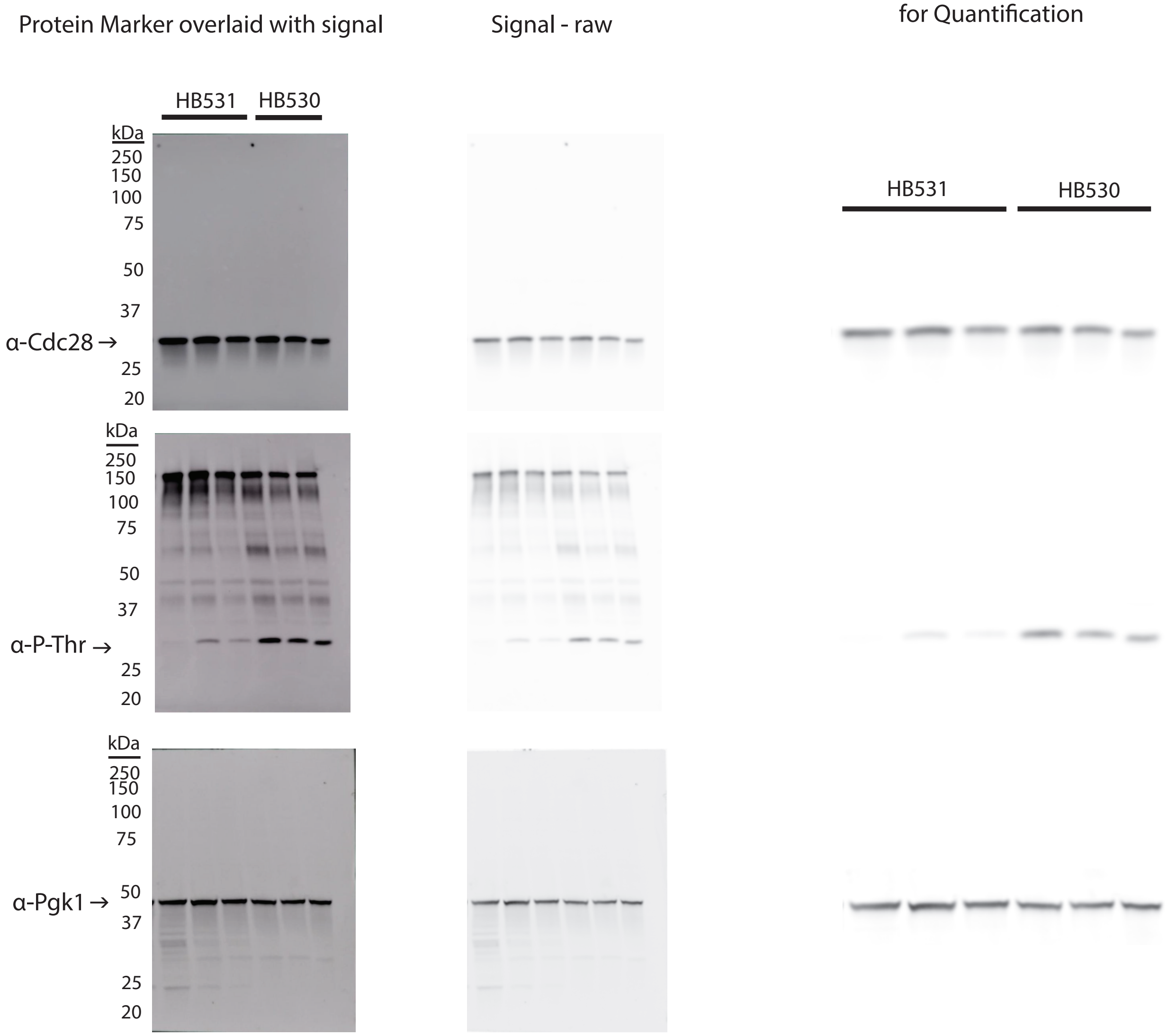

Supplement: Supplementary file 11 [file 44319_2026_916_MOESM7_ESM.zip › FIGURE 6/Figure6_RawBlots.pdf]
